# LD_2_SNPing: linkage disequilibrium plotter and RFLP enzyme mining for tag SNPs

**DOI:** 10.1186/1471-2156-10-26

**Published:** 2009-06-06

**Authors:** Hsueh-Wei Chang, Li-Yeh Chuang, Yan-Jhu Chang, Yu-Huei Cheng, Yu-Chen Hung, Hsiang-Chi Chen, Cheng-Hong Yang

**Affiliations:** 1 Department of Biomedical Science and Environmental Biology, Kaohsiung Medical University, Kaohsiung, Taiwan; 2 Graduate Institute of Natural Products, College of Pharmacy, Kaohsiung Medical University, Kaohsiung, Taiwan; 3 Center of Excellence for Environmental Medicine, Kaohsiung Medical University, Kaohsiung, Taiwan; 4 Department of Chemical Engineering, I-Shou University, Kaohsiung, Taiwan; 5 Institute of Molecular and Cellular Biology, National Tsing Hua University, Hsinchu, Taiwan; 6 Department of Electronic Engineering, National Kaohsiung University of Applied Sciences, Kaohsiung, Taiwan

## Abstract

**Background:**

Linkage disequilibrium (LD) mapping is commonly used to evaluate markers for genome-wide association studies. Most types of LD software focus strictly on LD analysis and visualization, but lack supporting services for genotyping.

**Results:**

We developed a freeware called LD_2_SNPing, which provides a complete package of mining tools for genotyping and LD analysis environments. The software provides SNP ID- and gene-centric online retrievals for SNP information and tag SNP selection from dbSNP/NCBI and HapMap, respectively. Restriction fragment length polymorphism (RFLP) enzyme information for SNP genotype is available to all SNP IDs and tag SNPs. Single and multiple SNP inputs are possible in order to perform LD analysis by online retrieval from HapMap and NCBI. An LD statistics section provides *D*, *D'*, *r*^2^, *δ*_*Q*_, *ρ*, and the *P *values of the Hardy-Weinberg Equilibrium for each SNP marker, and Chi-square and likelihood-ratio tests for the pair-wise association of two SNPs in LD calculation. Finally, 2D and 3D plots, as well as plain-text output of the results, can be selected.

**Conclusion:**

LD_2_SNPing thus provides a novel visualization environment for multiple SNP input, which facilitates SNP association studies. The software, user manual, and tutorial are freely available at .

## Background

Single nucleotide polymorphisms (SNPs) are very important markers for disease [1] and cancer [2] association studies. The number of identified SNPs is currently estimated to be about 3.1 million [3]. Identification of associations by statistical analyses of SNP data is challenging due to the large number of SNPs involved.

Linkage disequilibrium (LD) is one of the most commonly used methods when choosing informative SNPs that represent the original SNP distribution in a genome for genome-wide association studies. LD mappings are commonly used to evaluate markers across large data sets. Given the vast amount of data in association studies, visualization of the LD results in graphical form rather than text form facilitates the interpretation of the results considerably [4].

Many types of visualization software for LD have been developed, e.g. LDA [5], Haploview [6], and JLIN [7]. Although these tools have made valuable contributions to LD visualization and analysis, they lack many services and tools for users to generate genotype data for LD analysis. Without the actual data set itself, users are unable to perform LD analysis. However, many types of software exist which provide information for genotyping, e.g. the SNPlex genotyping system [8], SNP cutter [9], SNP-RFLPing [10], and V-MitoSNP [11]. These programs do not include an LD function though. It is thus still difficult for researchers to narrow down the number of SNPs for performing SNP genotyping. A common way of identifying tag SNPs of the genes of interest is to check the HapMap website [12]. Currently available tools, however, are not well integrated, but rather are independent programs.

We have thus integrated an SNP genotyping service and LD visualization/analysis tool in a single program to provide a single platform for tag SNP selection, SNP genotyping, and LD analysis. This platform, LD_2_SNPing, furthermore provides a novel function for multiple SNP inputs in order to directly plot the LD. The user can input SNPs of interest and calculate the LD measurement for SNP selection before the genotyping process. This stand-alone JAVA-based visualisation tool greatly facilitates preparation of the genotype data and increases the performance of LD analyses.

## Implementation

LD_2_SNPing is a Java-based software, which is implemented under the Java Runtime Environment (JRE) and Java 3D. The LD statistics program calculates *D*, *D'*, *r*^2^, *δ*_*Q*_, and *ρ *values, as well as the *P *value of Hardy-Weinberg Equilibrium (HWE-P) calculations for each SNP marker. LD_2_SNPing provides the *P *value of the Chi-square test and *P *value of the likelihood-ratio test for the pair-wise association of two SNPs are also provided in the LD calculation. LD_2_SNPing processes genotype data and estimates pair-wise loci haplotype frequencies of the sample using an expectation-maximization algorithm (EM) [13]. Except the exact tests of HWE [14] is implemented in LD_2_SNPing, the equations used in these calculations are listed in the appendix of the user manual as described by LDA [5].

In visualization of LD plot, the LD_2_SNPing software provides SNPs with a minor allele frequency (MAF) value greater than 0.01. All the MAF and HWE-*P *values for these SNPs are provided in the text window.

The SNP genotype information and the tag SNPs are retrieved online from dbSNP version BUILD 129 of NCBI [15][16] and HapMap  version HapMap Data Rel 23a/phaseII Mar08, on the NCBI B36 assembly, dbSNP b126 [12], respectively. Online retrieval for SNP genotype information from NCBI using SNP ID and gene input is similar to the function described in the SNP-Flankplus [17] and SNP ID-info [18]. The default setting for the minor allele frequency (MAF) cut-off in tag SNP from HapMap is 0.2. Four populations, CEU, CHB, JPT, and YRI (Caucasian, Han-Chinese, Japanese and Sub-Saharan African, respectively) are selectable during tag SNP retrieval from HapMap. The retrieved data are the most up-to-date data available. The RFLP database structure is based on REBASE [19] version 610. The RFLP mining function for the selected SNP is provided by the SNP-RFLPing [10], which is integrated in the LD_2_SNPing.

A demonstration and user manual of the LD_2_SNPing software are available as a free download from . Many animations explaining how to use the LD_2_SNPing software are provided on the homepage and embedded in the user manual (see Additional file 1) as tutorials.

## Results

### Data import formats: File input

LD_2_SNPing accepts four different input file formats, namely two Excel (.xls and .cvs), Word (.doc) and NotePad (.txt) formats. The first and second rows for each file are reserved for the user-defined SNP name and the distance between SNPs (optional), respectively. Individual genotypes accept the following formats: NN, N_N, and N/N (N is one of four possible nucleotides). If the input file is missing a genotype, it is automatically bypassed in LD_2_SNPing processing without interference. Some example files for testing are available in the example file folder of the LD_2_SNPing software package.

### Data import formats: rsID input

**Figure 1:**
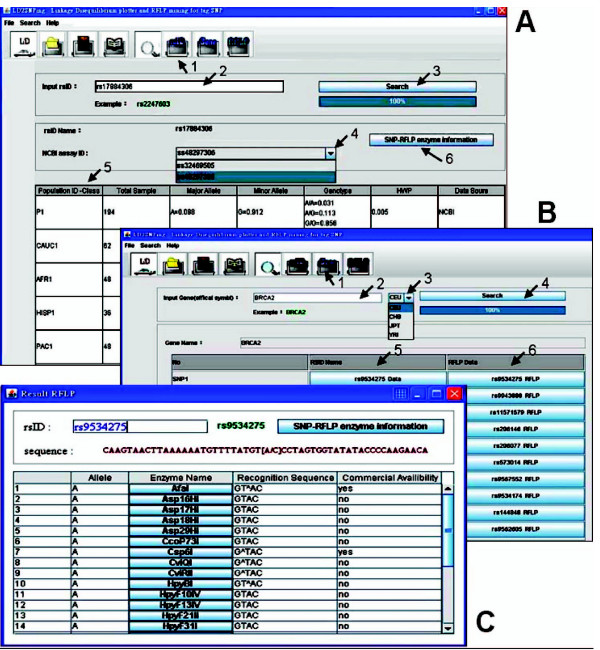
**Retrieval of SNP information by LD_2 _SNPing**. (A) rsID# input to retrieve SNP genotype information from dbSNP in NCBI. Arrows 1 to 5 indicate the steps to retrieve SNP information. The different ssID#s for the same rsID# can be selected as indicated by the arrow at line 4. Population class, total sample, major allele, minor allele, genotype frequencies, *P *value of Hardy-Weinberg equilibrium (HWE-*P*) and data source are provided as indicated by arrow 5. The RFLP information for inputting the SNP is provided (arrow line 6). (B) Gene input to retrieve tag SNPs from HapMap [12]. Four populations can be selected. The RFLP information for each tag SNP is also provided. (C) Restriction enzyme mining for RFLP in SNP genotype. Results similar to the RFLP information described in Figures 1B and 1C are shown.

LD_2_SNPing provides the rsID# input for online retrieval of individual SNP information from the dbSNP of the NCBI (Figure 1A).

### Data import formats: Gene input

LD_2_SNPing accepts gene name (HUGO, Human Genome Organization) input to provide tag SNPs through online retrieval from HapMap (Figure 1B).

### LD-free function: Retrieval of individual SNP information from NCBI

In Figure 1A, the SNP (rs17884306) information for all populations of the dbSNP is provided (P1, CAUC1, AFR1, HISP1, and PAC1). The ssID#s (ss32469505 and ss48297306) for the corresponding rsID# (rs17884306) can be selected by using the pull-down window.

### LD-free function: Gene input for finding rsID data of tag SNP

In Figure 1B, LD_2_SNPing provides the tag SNP information through HapMap by gene input. The example shown is BRCA2. The tag SNP candidates provided by LD_2_SNPing are completely matched with those of HapMap (shown in the user manual). HapMap-CEU, HCB, JPT and YRI are acceptable for selection.

### LD-free function: RFLP enzyme mining tool

Before performing LD analysis, it is necessary to collect SNP genotype data for genes of interest, such as the SNP ID input (Figure 1A) and tag SNPs (Figure 1B).

LD_2_SNPing executes RFLP restriction enzyme mining upon clicking of the RFLP box indicated by arrow 6 of Figure 1A and arrow 5 of Figure 1B. RFLP results are shown in the format pictured in Figure 1C, in which restriction enzyme information for SNPs of interest (here, rs9534275) are shown. Information about alleles, enzyme name, the recognition sequence and commercial availability is provided.

### LD function: Input formats for 2D analysis

**Figure 2:**
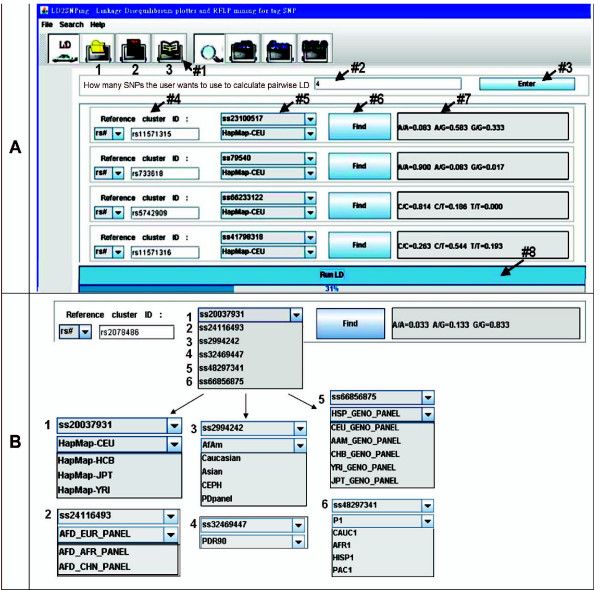
**Online retrieval of multiple SNP input for LD measurement, prediction, and visualization**. (A) Three LD functions are available in LD_2_SNPing: 1) user-defined files, 2) sample files, and 3) multiple SNPs. The input steps for multiple SNPs are demonstrated by arrows #1 to #8. Four SNPs, rs11571315, rs733618, rs5742909, and rs11571316, are used as examples. All the ssID#s with HapMap-CEU are selected. All selected SNP genotypes are provided after manually clicking the "Find" box. Similar results are shown in Figures 3 and 4. (B) Multiple choices for LD analysis of single rsID# SNP. In the example, rs2078486, six ssID#s are available. Each ssID# possesses different SNP data sets as indicated by numbers 1 to 6. The data is retrieved from NCBI and HapMap.

**Figure 3:**
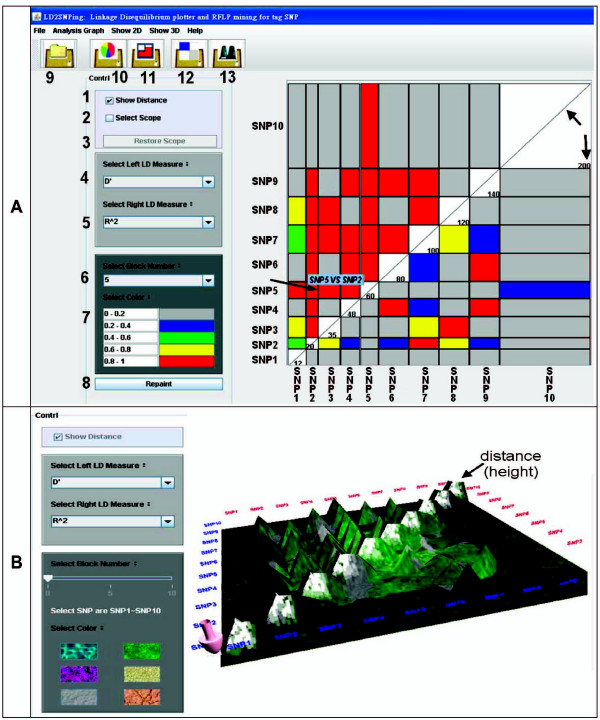
**Available functions in the control panel of 2D-LD and 3D-LD plots**. (A) The screen view after performing the LD calculation. The control panel contains eight functions 1) Select display of distance. 2) Scope selection of the 2D-LD plot. 3) Restore scope selection. 4) Pull-down selection of the left (vertebrate axis) LD measure. 5) Pull-down selection of the right (horizontal axis) LD measure. 6) The different colours of the blocks in the diagram in Figure 3 indicate a different degree of interaction of the SNPs. 7) User-defined colour selection. 8) Repainting of 2D-LD profile. The arrows indicate the distance between SNPs. The functions marked 9 to 13 represent indicate closing a file, pie3D graph, bar graph, homing in on 2D-LD plot, and 3D-LD visualization, respectively. The arrow pointing to the box of SNP5 vs. SNP2 is displayed by mousing-over. (B) 3D-LD visualization. The distances between SNPs are indicated by the height of the diagonal line.

LD_2_SNPing provides for file input and sample file input to perform LD analysis and visualization (numbers 1 and 2 of Figure 2A, respectively). Moreover, LD_2_SNPing provides for online retrieval of multiple SNP inputs for LD measurement, prediction and visualization (numbers #1 to #8 of Figures 2A and 2B). For convenience, the LD for any SNPs located on the same chromosome can be directly analyzed. Figure 2B shows the single SNP rsID# (rs2078486), which has six different ssID#s from different data sources. For example, ss20037931 has HapMap-CEU, HCB, JPT and YRI as data sources. Different data sources have different genotype frequencies for the same SNP rsID# due to the different data sets. The data was retrieved online from dbSNP of NCBI and confirmed to match (shown in user manual). Both file input and multiple SNP input lead to results similar with those shown in Figure 3, although the color pattern is different (described later).

### LD function: 2D-LD graph

The distance between SNPs supplied in the input file can be optionally displayed or hidden (number 1 of Figure 3A). This distance is shown next to the diagonal line as a numerical value. By clicking on the "select scope" (number 2 of Figure 3A) and "repaint" (number 8 of Figure 3A) buttons, a user can limit the number of SNPs shown to only those of interest. This view can be reversed by clicking on the "restore scope" (number 3 of Figure 3A) button. The parameters for LD measurement are selected by the two axes named "left and right LD measure" (numbers 4 and 5 of Figure 3A, respectively). Different color schemes for each of the statistics can be selected (numbers 6 and 7 of Figure 3A). Moreover, LD_2_SNPing provides a window for the minor allele frequency (MAF) value and HWE-*P *values for each analyzed SNP when LD analysis is performed (not shown). A more detailed description is given in the user manual.

### LD function: Data analysis of LD information

**Figure 4:**
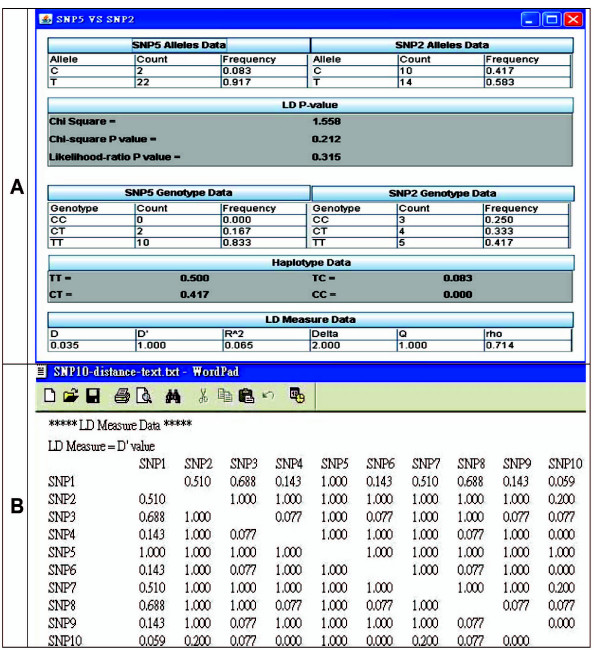
**Output sample of LD measurement information**. (A) Clicking on the box of the 2D-LD plot shows all LD-related information. SNP10-distance.xls in example file folder of LD_2_SNPing software is used as an example. Upon clicking the box of interest, such as the Box (SNP5 *vs*. SNP2) in Figure 3A (indicated by arrow line), the text data of this pair of SNPs for LD-related information is provided such as *D*, *D'*, *r*^2^, *δ*_*Q*_, *ρ*, Chi-square and likelihood-ratio *P *values, and the SNP and haplotype frequencies. (B) Output for LD-related values. Here, the *D' *value is shown as a representative example; other values are shown in the user manual. This file belongs to a WordPad file which is saved for output LD measurement data. It can be opened by WordPad and Microsoft Word.

LD_2_SNPing provides spontaneous analysis of the LD measurements for each pair-wise SNPs by clicking. For example, a text window (Figure 4A) will open when the arrow located in the box of SNP5 *vs*. SNP2 (Figure 3A) is clicked. In Figure 4A, the allele/haplotype frequencies, Chi-square *P *value, likelihood-ratio *P *value and all LD statistics (*D*, *D'*, *r*^2^, *δ*_*Q*_, and *ρ*) of paired SNPs are provided. These values are matched to the LDA software [5] (not shown).

In addition, LD_2_SNPing provides graphic analyses, such as grids and pie3D graphs, to supplement the 2D-LD visualization and analysis (numbers of 10 and 11 of Figure 3A). The results are shown in the user manual.

### LD function: 3D-LD graph

The 3D visualization of LD is performed by clicking on the icon for number 13 in Figure 3A. It is the same as in the 2D-LD plot except for the color patterns and the color ranges. In LD-3D, the distance and LD measurement values are indicated by the height in the diagonal line (Figure 3B). Users can toggle between the 2D-LD view or close the analysis by clicking on the icon for numbers 12 and 9 of Figure 3A, respectively.

### Data export

All the analyzed results can be saved as tab-delimited text files (.txt) and graphic files (.jpg) for convenience. The LD parameters are exported to a single file. Figure 4B shows a sample test result for "LD measure data", *D'*. All the *D' *values for each SNP are listed pairwise, a common publishing format. Other LD parameters are not shown here, but are available in the user manual.

## Discussion

### Comparison of some LD software

Many kinds of software for LD visualisation are freely available. LDA [5], Haploview [6], and JLIN [7] were written in Java to implement LD analyses. A comparison of the different LD software is shown in Table 1. LDA and JLIN provide many LD measurements, but LDA offers only limited options for visualization of the results. Some LD parameters are not provided by Haploview, e.g. *δ*_*Q *_and *ρ *values.

**Table 1:** Comparison of some LD software platforms

	LDA	JLIN	Haploview	LD_2_SNPing
Input file formats	txt	csv	ped, info, txt	xls, csv, txt, doc
Output file formats	print	pdf, eps, png, txt	png, txt	jpg, txt
*D*, *D'*, *r*^2^, *P*	✓	✓	✓	✓
*δ*_*Q*_, *ρ*	✓	✓		✓
Hardy-Weinberg equilibrium	✓	✓	✓	✓
Genotype/haplotype frequency		✓	✓	✓
Tag SNP mining by gene input				✓
SNP information retrieval by rsID input				✓
Enzyme mining for RFLP genotyping				✓
Online retrieval of multiple SNPs for LD plot				✓
2D distance		✓	✓	✓
2D graph visualization	✓	✓	✓	✓
2D graph/text data output		✓	✓	✓
3D distance/graph visualization				✓

Generally, SNP genotyping has to be performed to generate the SNP genotypes needed for LD analysis. Before performing LD analysis, however, all of the available LD software platforms only provide LD measurements without providing supporting functions, such as tag SNP mining by gene input, retrieval of SNP information, or RFLP enzyme mining for genotype. These supporting functions are provided in LD_2_SNPing (Table 1). Moreover, LD_2_SNPing allows for input of multiple SNPs for LD analysis (Figure 2). The genotype information of input SNPs are retrieved online from NCBI and HapMap. Therefore, users have an overview of the LD analysis for the input SNPs without performing prior SNP genotyping or inputting the genotype file. In contrast, Haploview provides many SNPs and users must manually select SNPs of interest. If the SNPs of interest are distributed widely over the chromosome, the SNP panel contains a large number of SNPs. Haploview thus only indirectly provides LD analysis for multiple SNPs.

### Tag SNP selection

Tag SNP selection candidates from different operation times in HapMap may not be consistent due to changes made in the built-in greedy algorithm. Some tag SNPs may or may not be found again in subsequent tests. For example, tag SNP selection by inputting gene BRCA2 to HapMap under MAF = 0.2 yields two tag SNP sets: 1) rs9534342, rs9943888, rs11571662, rs206120, rs206342, rs542551, rs9567552, rs206079, rs9562605, and rs14448 and 2) rs9534275, rs9943888, rs11571579, rs206146, rs206077, rs573014, rs9567552, rs9534174, rs144848, and rs9562605.

### Restriction enzyme mining for RFLP

The LD_2_SNPing provides the SNP ID searching to online retrieval to dbSNP in NCBI for RFLP analysis. However, the RFLP analysis for SNP ID input may be unable to provide the restriction enzyme information due to the nature of SNP itself. For example, the sequence information for rs9943888 and rs11571579 are retrieved successfully in LD_2_SNPing but only rs11571579 has the suitable restriction enzymes to mine (not shown). This is the nature for the SNP itself but not the RFLP analysis system error. For the wet experiment of PCR-RFLP, the users need the primer design software such as the "Prim-SNPing" [20] for primer design for SNP-RFLP and "SNP-Flankplus" [17] for the retrieval of SNP flanking sequence for primer design.

## Conclusion

LD_2_SNPing has the following characteristics: 1) it provides a search function for online retrieval of SNP information from dbSNP of NCBI; 2) it provides gene-centric tag SNP selection through online retrieval from HapMap; 3) all the SNP IDs and tag SNPs are processed to mine RFLP restriction enzymes for SNP genotype; 4) it provides LD measurements for *D*, *D'*, *r*^2^, *δ*_*Q*_, and *ρ*, along with the *P *value of the Hardy-Weinberg Equilibrium for each SNP marker and the *P *values of the Chi-square and likelihood-ratio tests for the pair-wise association of two SNPs in LD calculation; 5) it accepts multiple SNP inputs to perform LD analysis by online retrieval from HapMap and NCBI; 6) it presents both 2D and 3D visualization with LD-related measurements shown on the graphs; 7) it provides both graphic and plain-text outputs for LD analysis. In conclusion, LD_2_SNPing is a novel and integrated visualisation software designed to provide the user with the tools necessary for genotyping and LD analysis. It provides a simple and user-friendly interface with integrated functions for retrieval of SNP information, LD statistical calculation, analysis and visualization.

## Availability and requirements

**Project name**: LD2SNPing: Linkage disequilibrium plotter and RFLP enzyme mining for tag SNPs

**Project home page**:  with software and user manual for download.

**Operating system(s)**: Platform-independent

**Programming language**: Java

**Other requirements**: Java 1.5.0 or higher

**License**: Free for non-commercial use

**Any restrictions to use by non-academics**: Please contact corresponding author.

## Abbreviations

SNP: single nucleotide polymorphism; LD: linkage disequilibrium; RFLP: restriction fragment length polymorphism; HWE: Hardy-Weinberg Equilibrium; EM: expectation-maximisation algorithm; HUGO: Human Genome Organization; MAF: minor allele frequency.

## Authors' contributions

HWC and LYC wrote the manuscript. LYC provides the genomics information and LD-related statistics. YJC designed and developed the Java implementation of the underlying algorithms and GUI. YHC improved the RFLP performance and online retrieval for SNP information. HWC instructed HCH and HCC regarding software testing, improvement, and animation preparation. CHY coordinated and oversaw this study. All authors read and approved the final manuscript.

## Supplementary Material

Additional File 1 **User manual for LD_2_SNPing**. User manual for LD_2_SNPing. Click here for file
