# Supplementary material for: LD2SNPing: linkage disequilibrium plotter and RFLP enzyme mining for tag SNPs
Source: BMC Genet. 2009 Jun 6;10:26. doi: 10.1186/1471-2156-10-26 (PMC2709117; doi:10.1186/1471-2156-10-26)
Supplement: Additional File 1 — User manual for LD2SNPing. User manual for LD2SNPing. [file 1471-2156-10-26-S1.pdf]

$LD_2SNPing$ v2.0

Linkage disequilibrium plotter and RFLP mining for tag SNPs

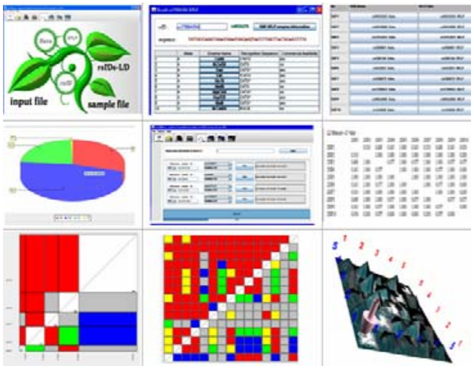

Contact:
chyang@cc.kuas.edu.tw
changhw@kmu.edu.tw
chuang@isu.edu.tw

Updated: 2009/05/07

LD$_2$SNPing v2.0 User Manual

1. Introduction … P. 4

2. Installation … P. 5

2.1. System Requirements … P. 5

2.2. Installing Java … P. 5

2.2.1. Installing JRE … P. 5

2.2.2. Installing Java3D … P. 5

2.3. Installing LD$_2$SNPING … P. 5

3. Input Format … P. 7

3.1. File input … P. 7

3.1.1. XLS and CVS Format … P. 7

3.1.2. DOC Format … P. 8

3.1.3. TXT Format … P. 8

3.2. Gene input… P. 9

3.3. rsID input … P. 10

3.3.1. Retrieval of the individual SNP information from NCBI … P. 10

3.3.2. Retrieval of genotype frequency of different populations for multiple SNPs from NCBI … P. 11

3.4. Sample file input … P. 12

4. Function of LD$_2$SNPing … P. 13

4.1. Brief review for the function of LD2SNPing … P. 13

4.2. LD-free functions … P. 14

4.2.1. Single rsID information browser and RFLP enzyme mining … P. 14

4.2.2. Gene input to find rsID data of tagSNP and RFLP enzyme mining … P. 16

4.2.3. RFLP enzyme mining tool using rsID input … P. 18

4.3. LD-available functions … P. 19

4.3.1. File input for LD calculation/visualization … P. 19

4.3.2. Sample file input for the tutorial of LD calculation /visualization … P. 20

4.3.3. Multiple rsID/ssID information browsers for LD calculation/visualization … P. 22

4.3.4. rsID# and ssID# searching for SNP frequency information … P. 25

4.3.5. LD calculation and 2D visualization … P. 28

4.3.5.1. Eight functions in control panel of 2D-LD plot … P. 28

4.3.5.2. Showing/omitting the distance between SNPs … P. 29

4.3.5.3. Scope selection from large to small area of 2D-LD plot … P. 30

4.3.5.4. Visualization of parameter changing of 2D-LD plot … P. 31

4.3.5.5. Color setting for 2D-LD display graphics … P. 32

4.3.5.6. LD measure text data for all SNPs … P. 35

4.3.5.7. Save text file for output LD measure data … P. 36
4.3.5.8. Open text file for output LD measure data … P. 37
4.3.5.9. Save and open image file for output LD measure data and graph … P. 38
4.3.5.10. Comparison of LD plotting between JLIN and $LD_2SNPing$ … P. 39
4.3.5.11. Comparison of LD plotting between LDA and $LD_2SNPing$ … P. 40
4.3.5.12. Comparison of LD plotting between Haploview and $LD_2SNPing$ … P. 41
4.3.6. Graph analysis-related functions in 2D-LD plot … P. 42
4.3.6.1. Brief review for five graph analysis-related functions in 2D-LD plot … P. 42
4.3.6.2. Close file … P. 43
4.3.6.3. Analysis graph … P. 44
4.3.6.3.1. Brief review for all functions in analysis graph … P. 44
4.3.6.3.2. Analysis graph function- Grid graph … P. 45
4.3.6.3.3. Analysis graph function- Bar graph … P. 46
4.3.6.3.4. Analysis graph function- Pie graph … P. 47
4.3.6.3.5. Return to 2D-LD plot … P. 48
4.3.7. LD in 3D visualization … P. 49
4.3.7.1. The difference between 2-D and 3-D plotting… P. 49
4.3.7.2. Performing 3D-LD and changing the parameters in 3D-LD plot … P. 49
4.3.7.3. Representative view of 3D-LD plot … P. 50
4.3.7.4. Zoom-in, zoom-out and rotation of 3D-LD plot … P. 51
4.3.7.5. Changing color for 3D-LD plot … P. 52
4.3.7.6. Selection of block number for 3D-LD plot … P. 53
5. Help … P. 54
6. Appendix… P. 55-56
7. References … P. 57

1. Introduction

Single nucleotide polymorphisms (SNPs) are the most common genetic polymorphisms in the human genome. They are increasingly important to the personalized medicine and many association studies. However, too much SNPs may make it hard to identify the interesting SNPs associated with diseases or cancers. Accordingly, it is essential to use a small representative subset of informative SNPs for the association studies.

Linkage disequilibrium (LD) is one of the common methods to identify these representative SNPs, called tag SNPs (tSNPs). Here, we developed the LD_2SNPing to compute LD measurement and visualize in 2D and 3D plots for user's input data file or on-line retrieval for multiple SNPs from HapMap and NCBI. Gene input to provide the tag SNP from HapMap is available. SNP ID rs# input for the RFLP restriction enzyme information for SNP genotype is implemented. Software, user manual, and video tutorial can be downloaded freely in http://bio.kuas.edu.tw/LD2SNPing. Many animations were provided in the end of each figure legend to help the user to practice the example.

2. Installation

$LD_2SNPing$ is implemented with java code and supported with three-dimensional display. The system needs the Java Runtime Environment (JRE) and Java 3D to implement and maintain the system works. The Java 3D have been packaged in $LD_2SNPing$. If your computer does not support any JRE software, you need to download the software in Sun's website (http://www.sun.com/) of JRE. Please see the following description for installation. Many animations were provided in the end of each figure legend to help the user to practice the example.

2.1. System Requirements

Programming language: Java Runtime Environment (JRE) needs to be installed.
Compute system: The software using in Pentium4 CPU system, 256M RAM and 15M of disk space.

2.2. Installing Java

2.2.1. Installing JRE

JRE is implementation of Java Application software by the sun. $LD_2SNPing$ is coded in Java, and users can download JRE accurate to operate. Users can download the latest version of JRE installed on their computers form http://www.sun.com/.

2.2.2. Installing Java 3D

Java 3D is implementation of a Java three-dimensional Application software by the sun. $LD_2SNPing$ can show three-dimensional in graphics. In order to complete the presentation of graphics, the Java 3D have been packaged in $LD_2SNPing$. Java 3D needs the environment in DirectX or OpenGL. $LD_2SNPing$ also needs to use software of Microsoft developed by a standard DirectX. If users are unable to implement the $LD_2SNPing$ programs, it is necessary to install the update version in http://www.microsoft.com/windows/directx/default.aspx.

2.3. Installing $LD_2SNPing$

Before the implementation, users have to make sure the Java platform application environment has been set correctly. The latest version of the $LD_2SNPing$ ($LD_2SNPing$ V2.0.exe) can be downloaded from the http://bio.kuas.edu.tw/LD2SNPing and it is set

up step by step with instruction. This software is developed and used in the Windows operating system. LD$_2$SNPing V2.0.exe can run in any Windows platform, but 3D computing needs greater demand for memory function, memory requirements proposed in more than 256 M RAM. Animation (Please click the box to demonstrate.)

Most functions need the internet on-line to retrieve all the necessary information except the LD calculation using file input.

3. Input Format

Before introducing the function of LD$_2$SNPing, we firstly list some acceptable input formats such as file, gene name, and rsID# as follows.

3.1. File input

LD$_2$SNPing accepts four kinds of input file formats, such as Excel (.xls and .cvs), Word (.doc) and NotePad (.txt) (in Fig. 1, Fig. 2, and Fig. 3 respectively). The first row for each file is for SNP name (user can type any names). The second row for each file is for distance (optional). More example files are available from example file folder of LD$_2$SNPing (described later in Fig. 15). It is available in the subfolder under the program file folder of LD$_2$SNPing.

3.1.1. XLS and CVS Formats

In Fig. 1, LD$_2$SNPing accepts some input formats (e.g., .xls and .cvs). The SNP ID name and the distance between them are optionally input if necessary. The distance value shown in SNPn is the distance between SNPn to zero point. A SNP genotype is composed of two alleles, the generally format of A, G, C, or T, but N represents the missing data.

A

B

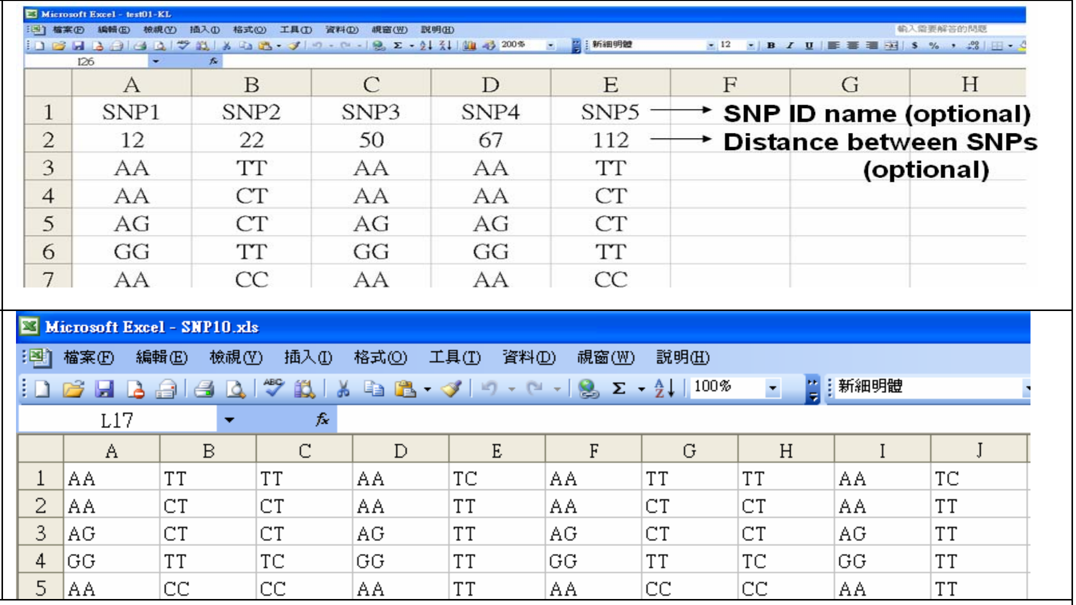

Fig. 1. Input data of XLS and CVS formats. (A) The input data format shows the SNP names, distances and genotypes. This file is available from *example file folder* of LD$_2$SNPing (e.g., test01.xls) (described later in Fig. 15). Users can view these files by inputting file. (B) File without SNP name or distance is acceptable in LD$_2$SNPing (e.g., SNP10.xls). The SNP name is given in the order of SNP1 to SNPn from left to right side in default, i.e., columns A, B, C, D, E, F, G, H, I, and J are set to SNP1, 2, 3, 4, 5, 6, 7, 8, 9, and 10, respectively.

3.1.2. DOC Format

In Fig. 2, LD$_2$SNPing accepts input format in .doc file. File without SNP name or distance is acceptable in LD$_2$SNPing. The SNP name is given in the order of SNP1 to SNPn from left to right side in default.

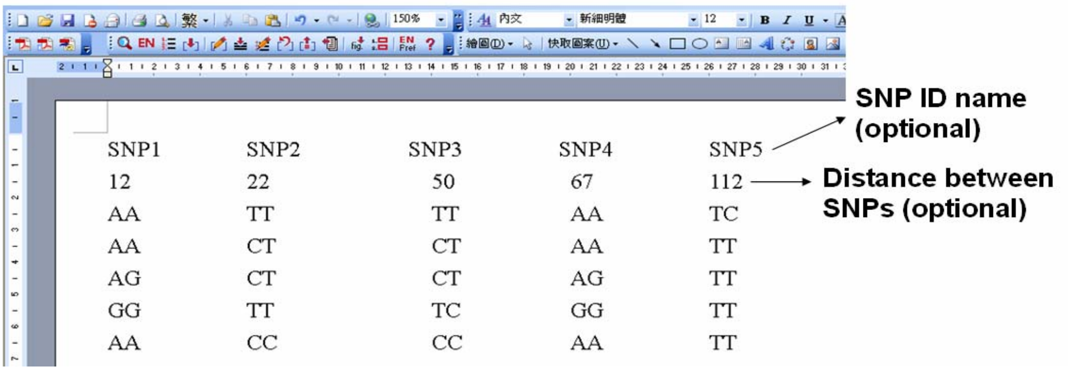

Fig. 2. Input data of Word format. The input data format shows the SNP names, distances and genotypes. This file (test02.doc) is available from *example file folder* of LD$_2$SNPing (Fig. 15). If user install the LD$_2$SNPing in C, then the path to get the example file is followed, C:\Program Files\LD2SNPing\example.

3.1.3. TXT Format

In Fig. 3, LD$_2$SNPing accepts input format in .txt file. File without SNP name or distance is acceptable in LD$_2$SNPing. The SNP name is given in the order of SNP1 to SNPn from left to right side in default.

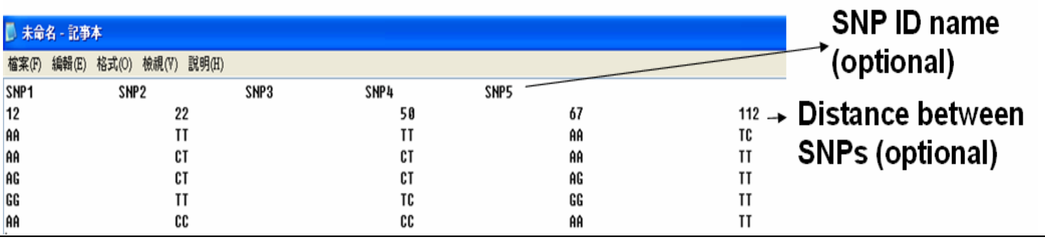

Fig. 3. Input data of Txt format. The input data format shows the SNP names, distances and genotypes. This file (test03.txt) is available from example file folder of LD$_2$SNPing (described later in Fig. 15).

3.2. Gene input

In Fig. 4, LD$_2$SNPing accepts gene name (HUGO, Human Genome Organization) input to provide the tagSNP from on-line retrieval to the HapMap. Therefore, the retrieval data are always the most updated as the same as the current version of HapMap (HapMap Data Rel 23a/phaseII Mar08, on NCBI B36 assembly, dbSNP b126.) (http://www.hapmap.org).

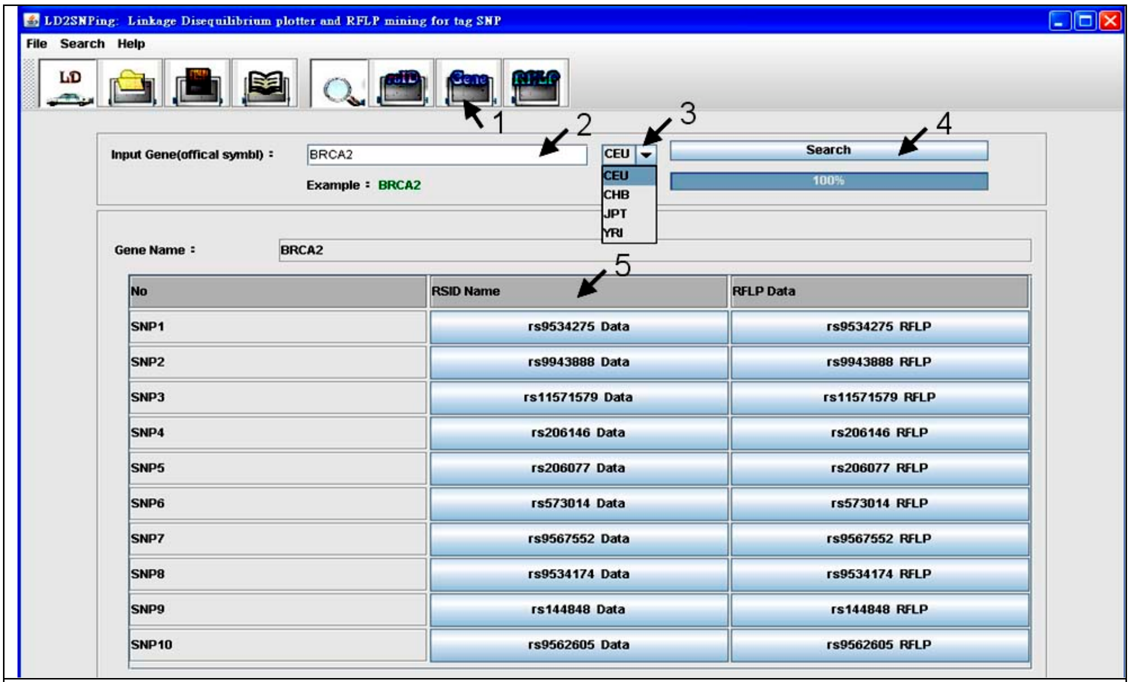

Fig. 4. Input of gene name. The input data format shows the SNP nos., SNP rsID# and their corresponding RFLP data. The procedures to retrieve the HapMap tagSNP from gene name input (e.g., BRCA2) are indicated from arrow 1 to arrow 5. In arrow 4, the original rsID information in dbSNP of NCBI and its SNP-RFLP enzyme information are provided by clicking the box. *[HapMap website: http://www.hapmap.org]* Animation

3.3. rsID input

3.3.1. Retrieval of the individual SNP information from NCBI

LD$_2$SNPing provides the rsID# input to retrieve the individual SNP information from dbSNP of NCBI on-line (Fig. 5). Therefore, the SNP information for all population existed in current version of dbSNP is provided. The ssID# for corresponding rsID# is selective using pull-down window.

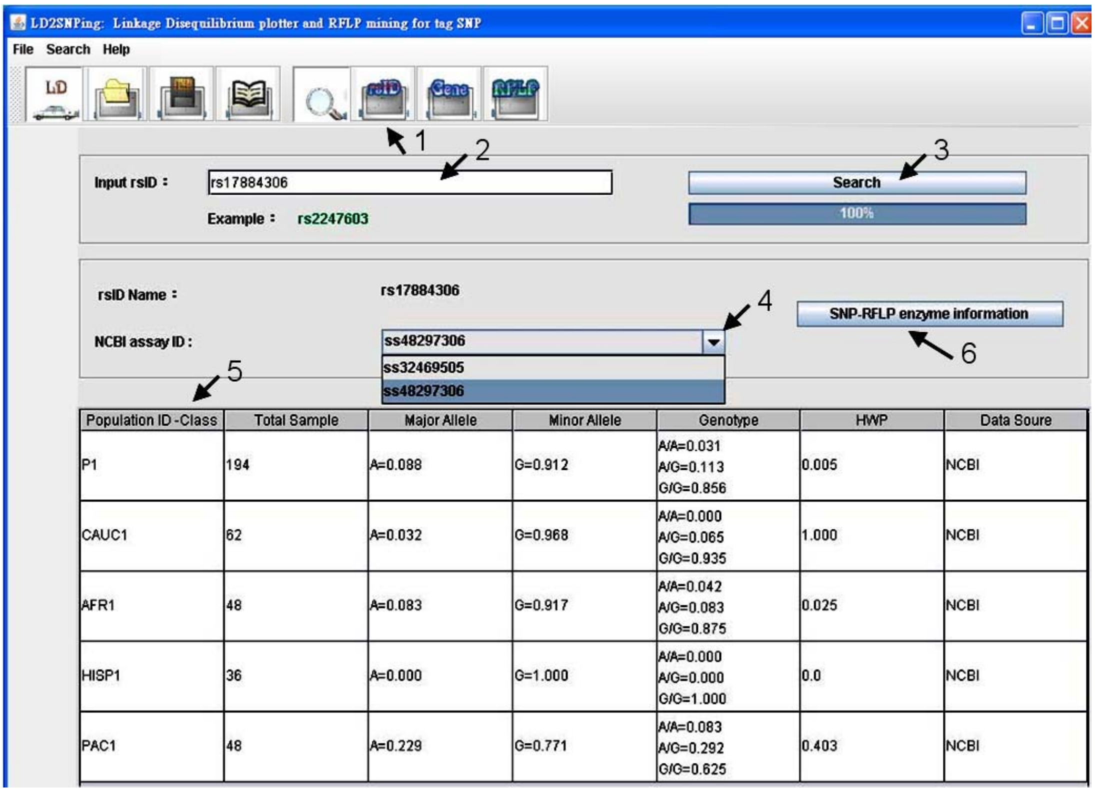

Fig. 5. rsID# Input. The input procedures to retrieve the individual SNP rsID# (e.g., rs17884306) are indicated from arrow 1 to arrow 5. The arrow 4 indicates the pull-down window for ssID# selection. Subsequently, the SNP genotype information for selected ssID# is provided as indicated by arrow 5. The arrow 6 indicates the function for SNP-RFLP enzyme information by clicking if necessary. **HWP** = P value of Hardy-Weinberg Equilibrium.

Animation

3.3.2. Retrieval of genotype frequency of different populations for multiple SNPs from NCBI

Alternatively, users may need to retrieve several interested SNPs from NCBI using rsID# and ssID# for further LD calculation and visualization (Fig. 6, only input action is shown here and the output result is shown in output section later). LD$_2$SNPing provides the method for users to input some SNP IDs and automatically retrieve the SNP frequency information from dbSNP in NCBI on-line (Fig. 6). LD measurements between these SNPs are provided without the knowledge of the SNP information for these SNP IDs (see output section later).

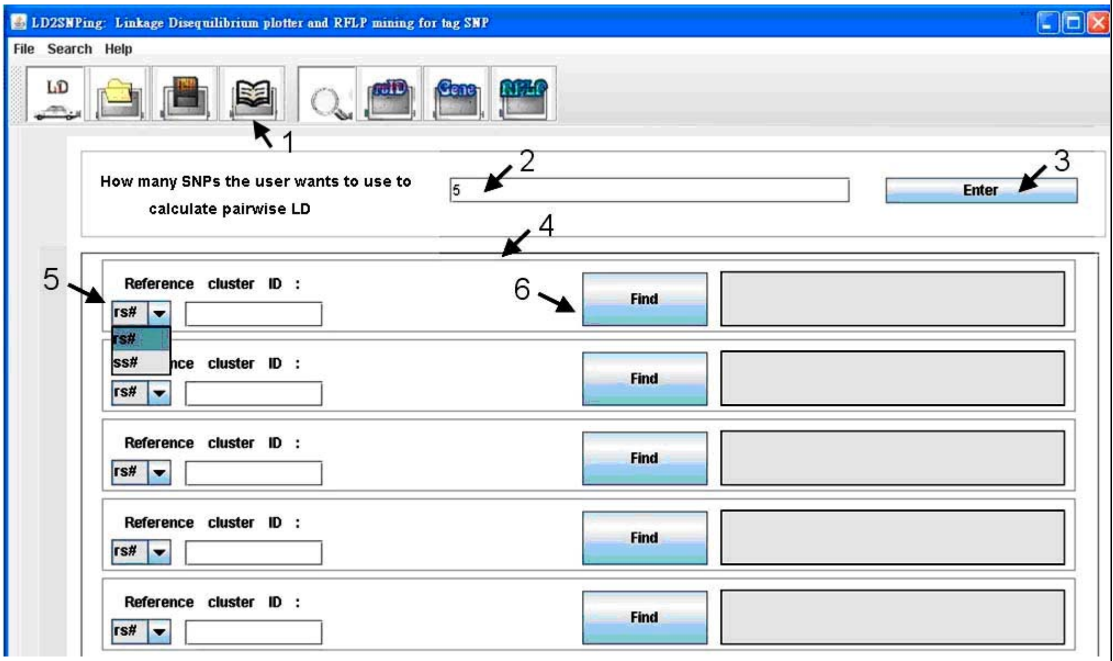

Fig. 6. Search rsID input type. The input procedures to retrieve several interested SNPs from NCBI using rsID# and ssID# inputs for LD calculation and visualization are indicated from arrow 1 to arrow 6. In arrow 2, users can key in the desired numbers for SNP ID input. Subsequently, the box numbers for requested SNP numbers are immediately generated (arrow 4). The arrow 5 indicates the pull-down window for rsID# and ssID# selections. Once the rsID# is selected, several corresponding ssID# are interchangeable if they are provided in dbSNP of NCBI (no shown here; it will described later). Clicking the box indicated by arrow 6 shows the SNP genotype frequency for selected population which is on-line retrieved from dbSNP of NCBI. Each SNP genotype frequency is retrieved one-by-one by clicking the “find” box. Each SNP rsID# or ssID# for each input window is editable. Once finished, please click the “find” box again to update the search. Animation

3.4. Sample file input

LD$_2$SNPing provides four standard sample files (Fig. 7) for user to familiar with the acceptable file format (Figs. 1-3) and to test it. In sample 4, it is a real gene dataset downloaded from the HapMap site (http://hapmap.jst.go.jp/hapmappopulations.html). The characteristics of the sample file are provided. Actually, LD$_2$SNPing accepts the genotype format in the form of NN, N_N, and N/N (N is one of the nucleotides) (not shown).

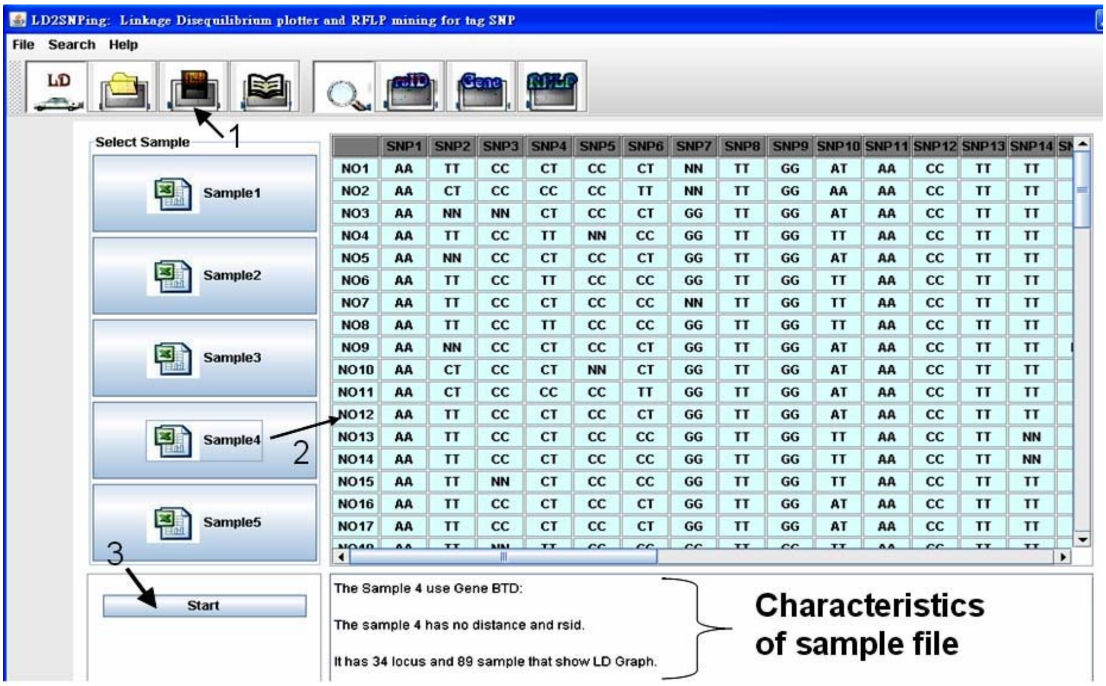

Fig. 7. Sample input type. Sample format from the HapMap gene BTD dataset. The column without SNP label in the first row is ignored by default. The procedures to show the sample file are indicated from arrow 1 to arrow 2. Animation

4. Function of LD$_2$SNPing

Performing the RunLD2SNPing.exe, users will enter the main screen of the LD$_2$SNPing system program (Fig. 8). ***Most functions need the internet on-line to retrieve all the necessary information except the LD calculation using file input.***

4.1. Brief review for the function of LD$_2$SNPing

Six functions were provided, including three LD-free functions and three LD-available functions as follows.

LD-free functions

1) Single rsID information browser and RFLP enzyme mining – On-line retrieval of individual SNP information among different populations from NCBI.

2) Gene input to find rsID data of tagSNP and RFLP enzyme mining – On-line retrieval of tagSNP in HapMap by HUGO gene name input.

3) RFLP enzyme mining tool using rsID input – RFLP enzymes are provided for SNP genotype using rsID# input.

LD-available functions

4) File input for LD calculation/visualization – Formats such as Excel (.xls and .cvs), Word (.doc) and NotePad (.txt).

5) Sample file input for the tutorial of LD calculation/visualization – These sample files show the format of SNP genotypes and provide the chance for users to practice the LD$_2$SNPing software.

6) Multiple rsID/ssID information browsers for LD calculation/visualization – On-line retrieval of genotype frequency of different populations for multiple SNPs from NCBI.

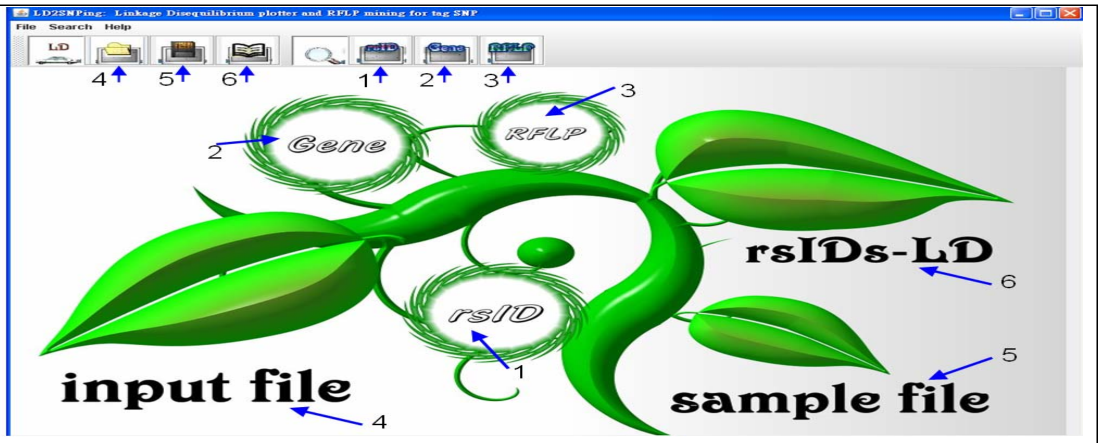

Fig. 8. LD$_2$SNPing entry screen and function. Arrows 1, 2, and 3 indicate LD calculator and visualization. Arrows 4, 5, 6 indicate SNP related search without LD function.

4.2. LD-free functions

4.2.1. Single rsID information browser and RFLP enzyme mining – On-line retrieval of individual SNP information among different populations from NCBI.

After inputting rsID# (e.g., rs17884306) and selecting ssID# (e.g., ss48297306), the population class, total sample, major allele, minor allele, genotype frequency, HWP (*P* value of the Hardy-Weinberg equilibrium) and data source are provided as shown in Fig. 9A. These data are completely matched to that of NCBI (http://www.ncbi.nlm.nih.gov/SNP/snp_ref.cgi?rs=17884306) as shown in Fig. 9B. Please note that different ssID# may change their corresponding genotype frequency. This is the nature characteristic of dbSNP in NCBI because these data were reported from different laboratories into different ssID# for the same rsID#. Clicking the example (rs2247603) automatically provides similar SNP ID retrieval (Fig. 14, later).

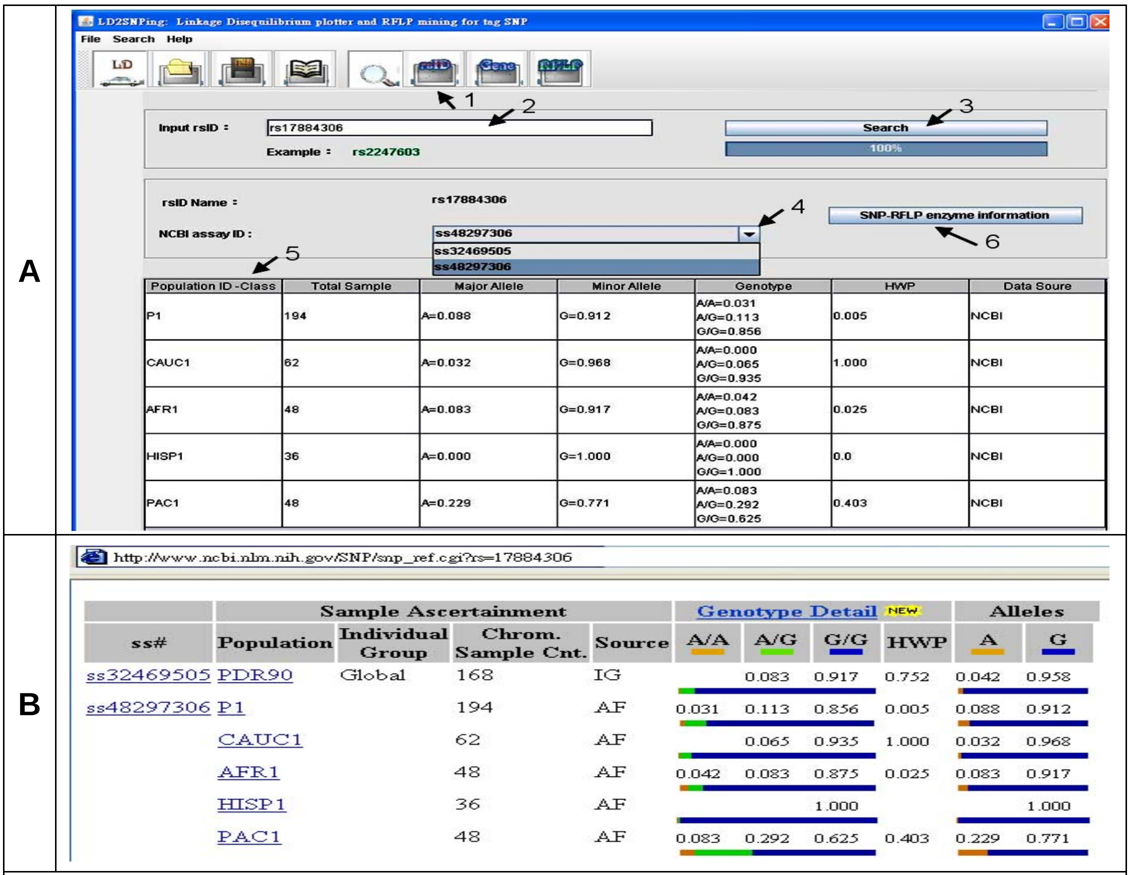

Fig. 9. rsID# input and output for LD$_2$SNPing and dbSNP of NCBI. (A) In LD$_2$SNPing, the input procedures to retrieve the individual SNP rsID# (e.g., rs17884306; ss48297306) are indicated from arrow 1 to arrow 5. The arrow 4 indicates the pull-down window for ssID# selection. The corresponding RFLP enzyme is the same for any ssID# because they share the same rsID#. The arrow 5 indicates the SNP genotype frequency for available population which is on-line retrieved from dbSNP in NCBI (*see* data source). The arrow 6 indicates the function for SNP-RFLP enzyme information by clicking and its result is shown in Fig. 10. (B) SNP information from dbSNP of NCBI.

A

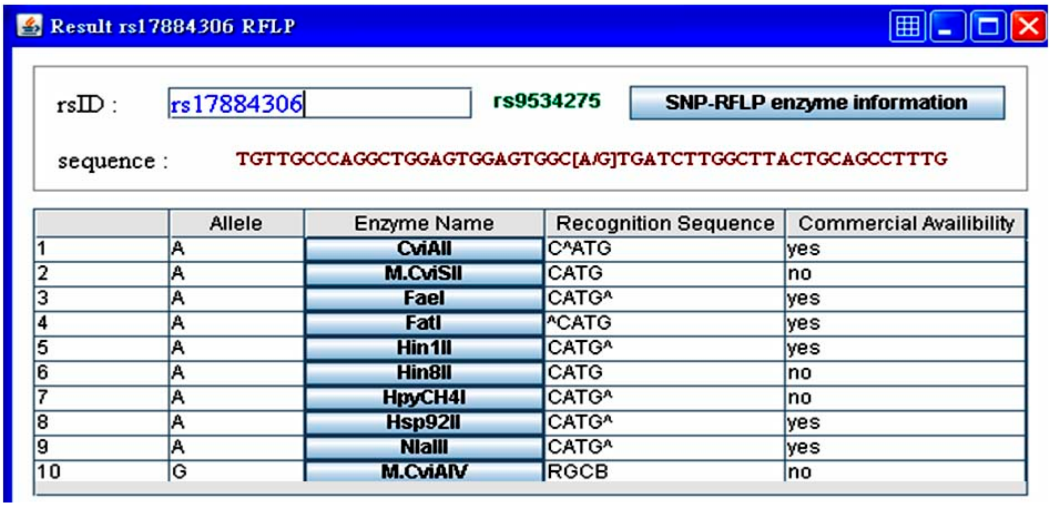

B

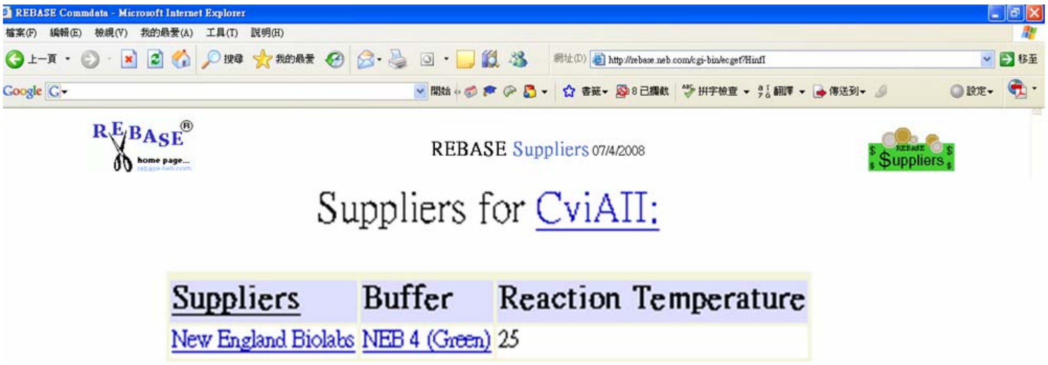

Fig. 10. SNP-RFLP enzyme information. (A) Output of SNP-RFLP enzyme mining information (Result of arrow 6 in Fig. 9.) (B) On-line link to external source (REBASE) for commercial available SNP-RFLP enzyme in the example of CviAII by click the first line in (A).

Animation

4.2.2. Gene input to find rsID data of tagSNP and RFLP enzyme mining – On-line retrieval of tagSNP in HapMap by HUGO gene name input.

In order to find output data from the gene-related information (e.g., BRCA2 and BRCA1 for Fig. 11 and Fig. 12, respectively), LD$_2$SNPing can provide the tagSNP information through the HapMap (http://hapmap.jst.go.jp/hapmappopulations.html). As demonstrated in Fig. 11 and Fig. 12, our proposed LD$_2$SNPing completely agrees with the tagSNP information provided by HapMap because LD$_2$SNPing is designed to retrieve this information on-line. Clicking each RFLP data box leads to the RFLP enzyme information as shown in Fig. 10.

A

B

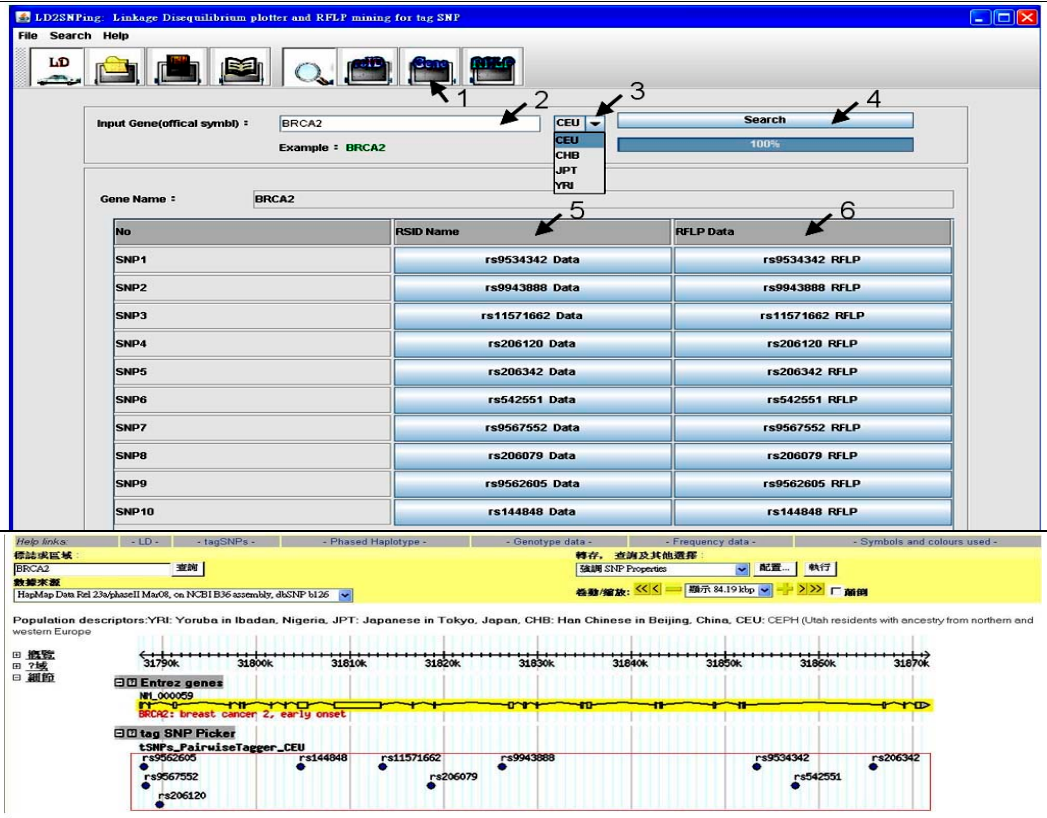

Fig. 11. Result of gene name (BRCA2) input using LD$_2$SNPing *vs.* HapMap. (A) The input data format shows the SNP nos., SNP rsID# and their corresponding RFLP data in LD$_2$SNPing. The procedures to retrieve the tagSNP picker of HapMap (Four populations such as CEU, CHB, JPT, and YRI are provided for selection, as indicated by arrow 3) as shown by red box in (B) from gene name input are indicated from arrow 1 to arrow 5 *[http://www.hapmap.org]*. The CEU population is selected for the example with minor allele frequency (MAF) cut off = 0.2. In arrow 6, the original rsID information in dbSNP of NCBI and its SNP-RFLP enzyme information is provided by clicking the box for each tag SNP one-by-one. The result of rsID# RFLP is similar to that of Fig. 10 (not shown here). (B) The tagSNP result of BRCA1 gene from HapMap (version NCBI B36 assembly, dbSNP b126). Animation ***Note!*** *Tag SNP selection candidates from different operation times in HapMap may be not be consistent completely due to the greedy algorithm built. Some tag SNPs may or may not find in next test. Likely, LD$_2$SNPing has the same performance.* Animation/Animation

A

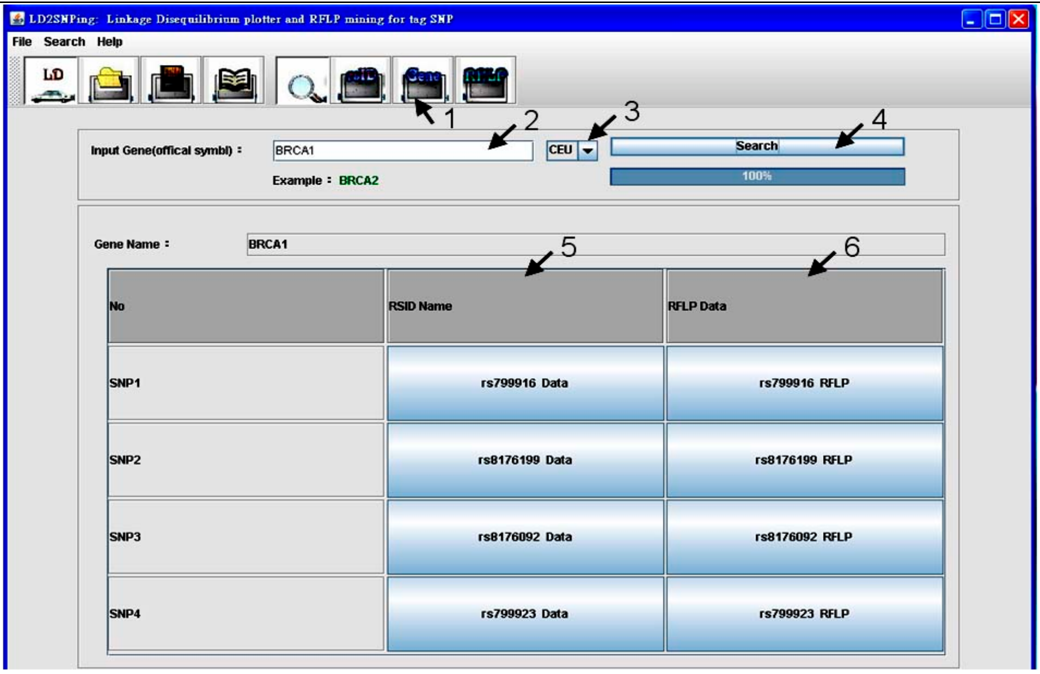

B

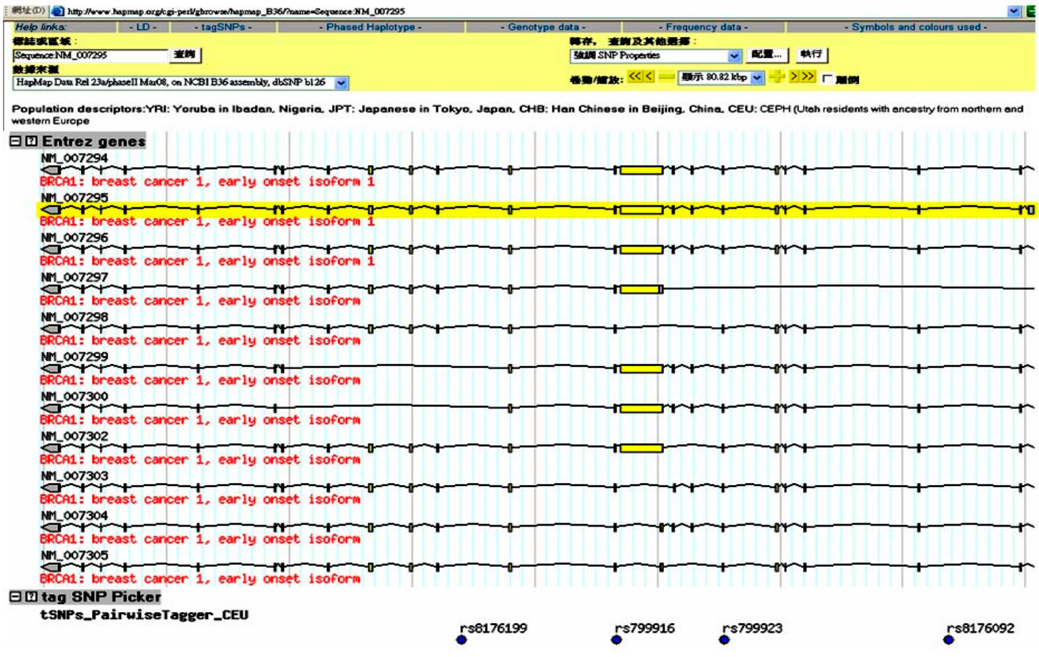

Fig. 12. Result of gene name (BRCA1) input using LD$_2$SNPing *vs.* HapMap. (A) The input data format shows the SNP nos., SNP rsID# and their corresponding RFLP data in LD$_2$SNPing. BRCA1 tagSNP is based on the record for NM_007295. The procedures to retrieve the tagSNP of HapMap [*http://www.hapmap.org*] (Four populations such as CEU, CHB, JPT, and YRI are provided for selection, as indicated by arrow 3) from gene name input are indicated from arrow 1 to arrow 5. The CEU population is selected for the example with minor allele frequency (MAF) cut off = 0.2. In arrow 5, the original rsID information in dbSNP of NCBI and its SNP-RFLP enzyme information is provided by clicking the box (arrow 6) for each tag SNP one-by-one. The result of rsID# RFLP is similar to that of Fig. 10 (not shown here). (B) The tagSNP result of BRCA1 gene from HapMap (version NCBI B36 assembly, dbSNP b126).

4.2.3. RFLP enzyme mining tool using rsID input – RFLP enzymes are provided for SNP genotype using rsID# input.

It is designed to provide RFLP enzyme information for SNP genotype before LD analysis. In the $LD_2SNPing$, restriction enzymes information for interested SNP (e.g., rs9534275) is provided as shown in Fig. 13. The restriction enzyme information is downloaded from REBASE. The implement of SNP-RFLP is similar to our previous publication, SNP-RFLPing (http://bio.kuas.edu.tw/snp-rflp).

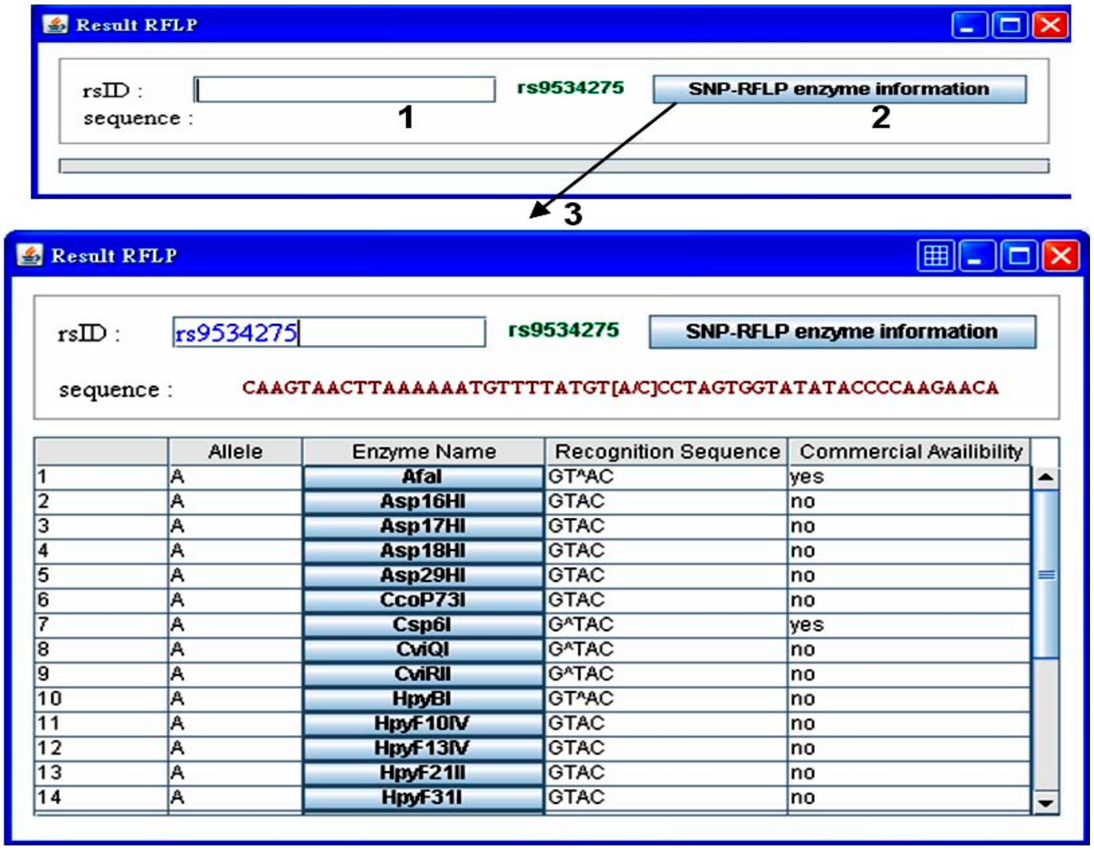

Fig. 13. Input rsID# to search RFLP enzyme data. The protocol is listed from arrows 1 to 3. 68 different of restriction enzymes are provided for the RFLP enzyme mining of rs9534275 using $LD_2SNPing$. Similar explanation is also shown in Fig. 10. Only some of the enzymes are listed here due to limited space.

4.3. LD-available functions

LD-available functions included: 4) File input, 5) Sample file, and 6) Multiple rsID/ssID information browsers for LD calculation/visualization.

Here we firstly introduce how to use these different inputs separately and briefly. Then, we show their detailed common LD calculation and 2-D visualization, graph analysis, and LD in 3-D visualization. They are described in detail as follows.

4.3.1. File input for LD calculation/visualization

$LD_2SNPing$ accepts four kinds of input file formats, such as Excel (.xls and .cvs), Word (.doc) and NotePad (.txt), as shown in Figs. 1, 2, and 3 respectively.

To start the file input function, users can select any interested files with SNP genotypes (Fig. 14). *Although the $LD_2SNPing$ can perform the LD function for several SNPs existed in different chromosomes, it is not the scope of LD definition.* In other words, several SNPs in the same chromosome with their corresponding SNP genotypes are necessary to perform LD calculation due to the definition of LD.

In Fig. 14, $LD_2SNPing$ provides several files for examples in default (as marked in 1) for users to familiar with the possible acceptable formats for .xls, .cvs, .doc, and .txt (as marked in 2 and 4). Alternatively, users can select their own file to perform this function by clicking the pull-down window as (marked in 3).

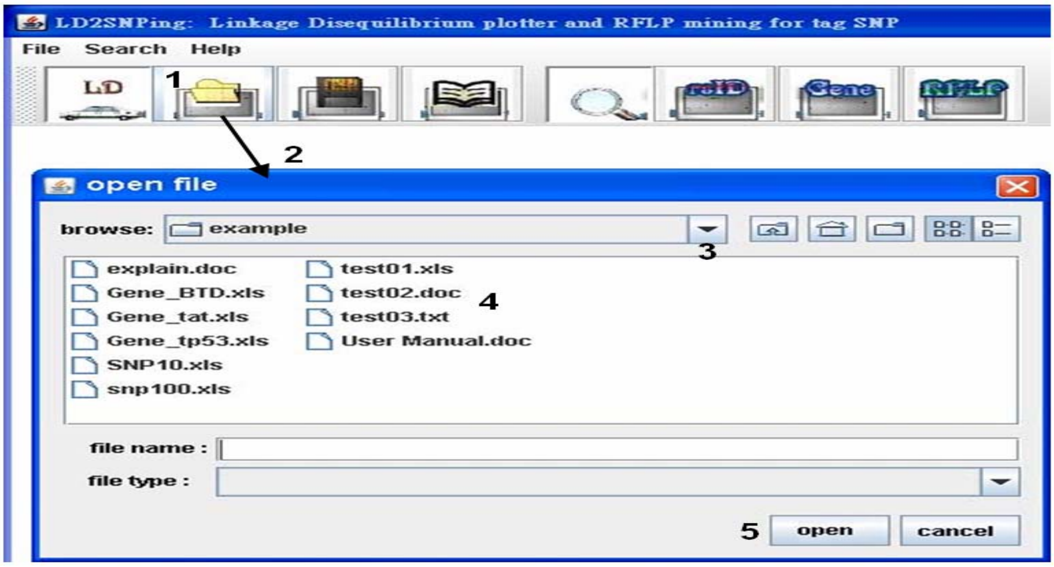

Fig. 14. File input procedure. Clicking the box 1 shows the box 2 (open file) which provides several different formats of SNP genotype data (box 4) for users to familiar with input. Users may click the box 3 (pull-down window) to select their interested file in their computer folds if necessary. Finally, users can click the box 5 to complete the open file. In the example of Gene_BTD.xls in window 4, the brief output result is shown in Fig. 15B. Similar detail result is shown in Fig. 20. Animation

4.3.2. Sample file input for the tutorial of LD calculation/visualization

These sample files provide the chance for users to practice the LD$_2$SNPing software. The SNP is defined by the nucleotide variant (allele) larger than 1% (Minimal allele frequency; MAF > 0.01) of the population. Therefore, for those allele frequencies less than 1% is not shown in the 2D-LD plot in default setting, such as the SNP1, 3, 5-9, 11-13, 15, 19, 22-26, 28, 30 and 32 of sample 4 in Fig. 15A. Those SNPs with MAF <0.01 are not visualized in the 2D-LD plot (Fig. 15B). All the MAF values for all SNPs are provided in “show SNP data” of “help” (Fig. 15C).

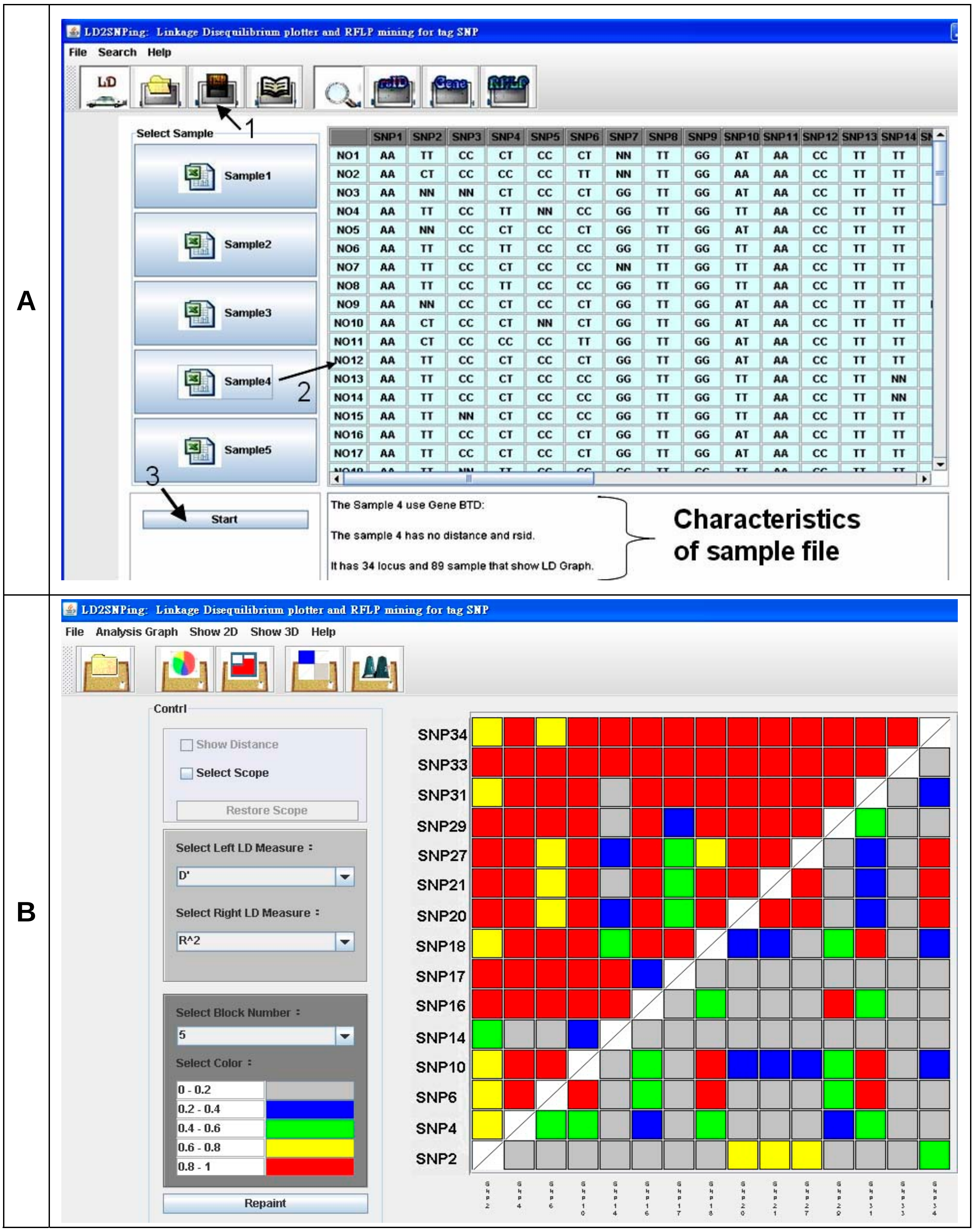

C

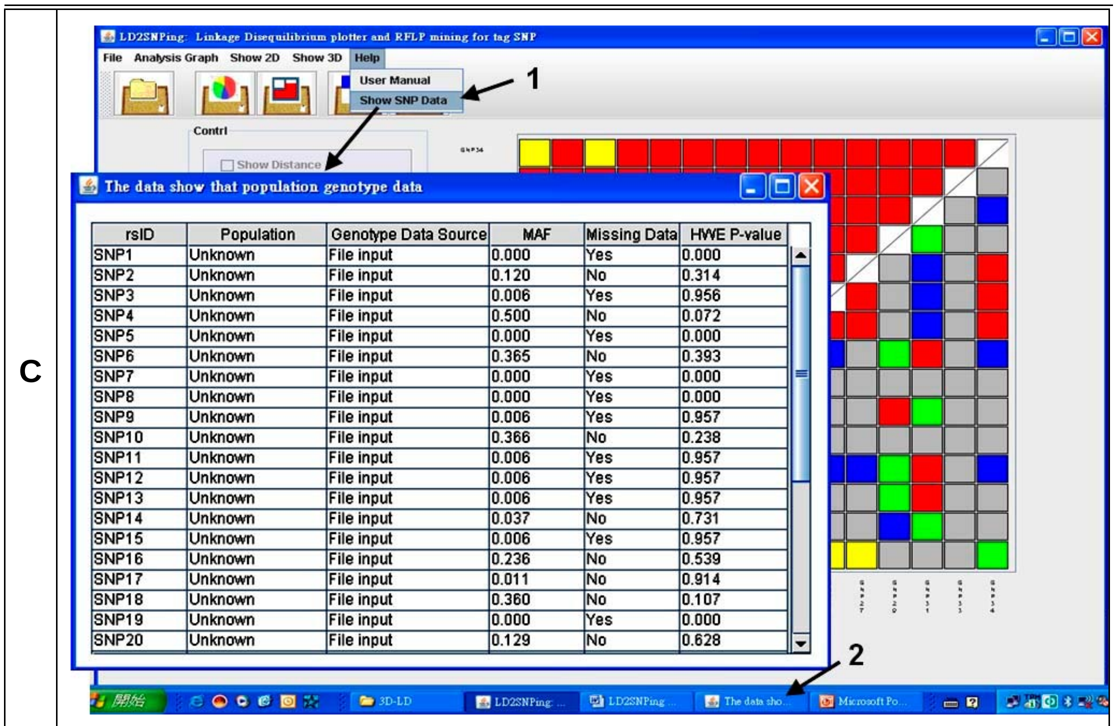

Fig. 15. Sample input type. (A) The procedure to input the sample file is indicated from arrows 1 to 3. <u>Sample 4 is used for the example</u> which is derived from the HapMap gene BTD dataset. The column without SNP label in the first row is ignored by default. NN indicates the no available SNP frequency in this SNP for some individuals, suggesting that some data missing is not interfere the LD analysis in LD$_2$SNPing. (B) The brief output result (Axis: D'/r^2). Similar detail result is shown in Fig. 20. (C) Missing data information and MAF values for input SNPs. It is provided immediately but hidden in the help function (arrow 1) or at the bottom of window system (arrow 2).

rsID item indicates the user's input names for SNPs. **Genotype data source** indicates the SNP genotype data input way by file input (Fig. 15) or NCBI retrieval (not shown here). **MAF** = Minimal allele frequency. **Missing data** indicates that SNPs with MAF < 0.01 and they are not shown in 2D-LD and 3D-LD plots in LD$_2$SNPing. **HWE P-value** indicates P value of Hardy-Weinberg equilibrium. Animation

4.3.3. Multiple rsID/ssID information browsers for LD calculation/visualization

Genotype frequency of different populations for multiple SNPs is retrieved from NCBI on-line. Therefore, the SNP information for all population exited in current version of dbSNP is provided. Five SNP rsID# with available genotype raw data of HapMap (Fig. 16) are demonstrated to perform LD visualization in Fig. 18A.

A

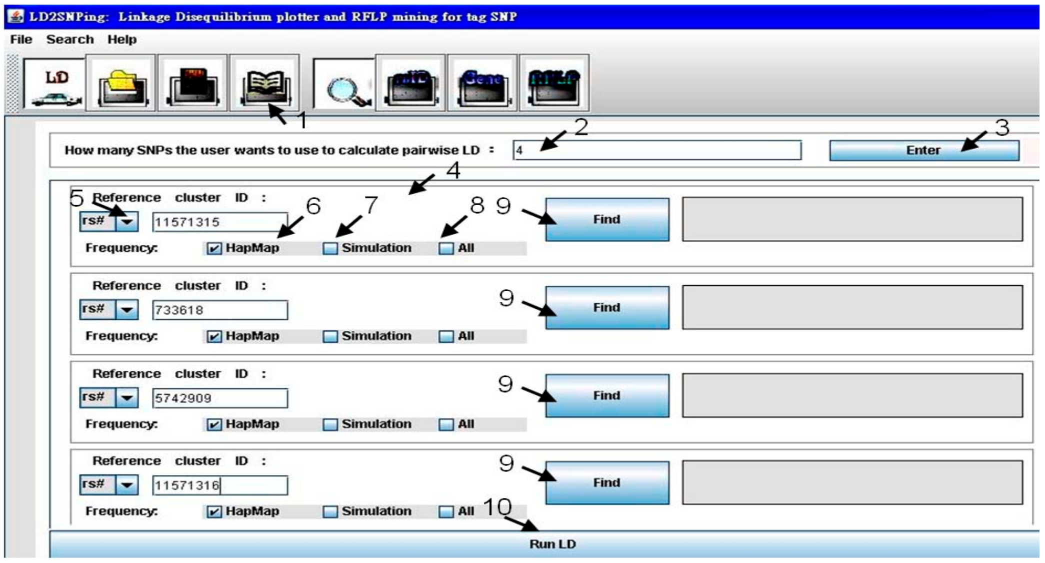

B

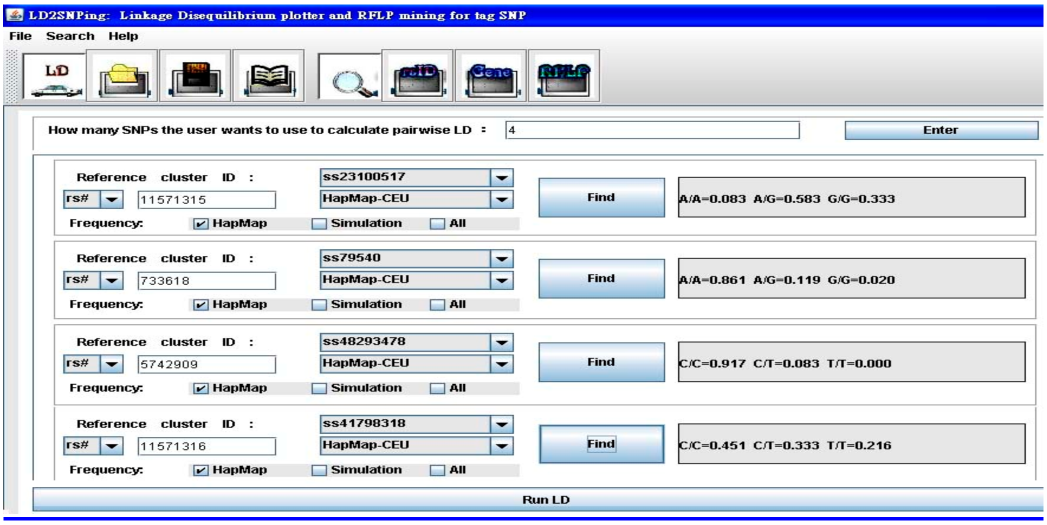

Fig. 16. Search rsID input type. First, users have to input the required SNP numbers at the top window (arrows 1 and 2). After clicking "Enter" (arrow 3), the system generates the same box numbers for SNP ID inputs. Input the rsID (e.g., rs11571315, rs733618, rs5742909, and rs11571316), select the population by pull-down window (arrow 5). Users are able to select the HapMap (arrow 6), simulation (arrow 7), or all (containing both HapMap and simulation; arrow 8). The SNP information is retrieved by clicking the "Find" box *one-by-one* (arrow 9). Then, the SNP frequency information appears. Once completing the SNP ID inputting, users can click "Run LD" at the bottom window (arrow 7) to show the LD relationship between these inputting SNPs (rs# or ss# are all acceptable). (B) The finished SNP ID input in the example of HapMap-CEU data source from HapMap. The brief output is shown in Fig. 18. Similar detail result is shown in Fig. 21.

The function for multiple rsID/ssID information browsers for LD calculation/ visualization in LD$_2$SNPing is *in direct manner* (Fig. 16). In contrast, the multiple rsID/ssID information browsers for LD calculation/visualization in Haploview are *in indirect manner* as shown in Fig. 17. The Haploview cannot accept the multiple SNP input directly. Instead, the Haploview provides all the SNPs within the user's input range and subsequently narrow down to user's interested SNPs for LD analysis by manually clicking one-by-one. In Fig. 17, the same four SNPs listed in Fig. 16 are used as example to perform LD analysis by Haploview and their 2D-LD result is shown in Fig. 18B.

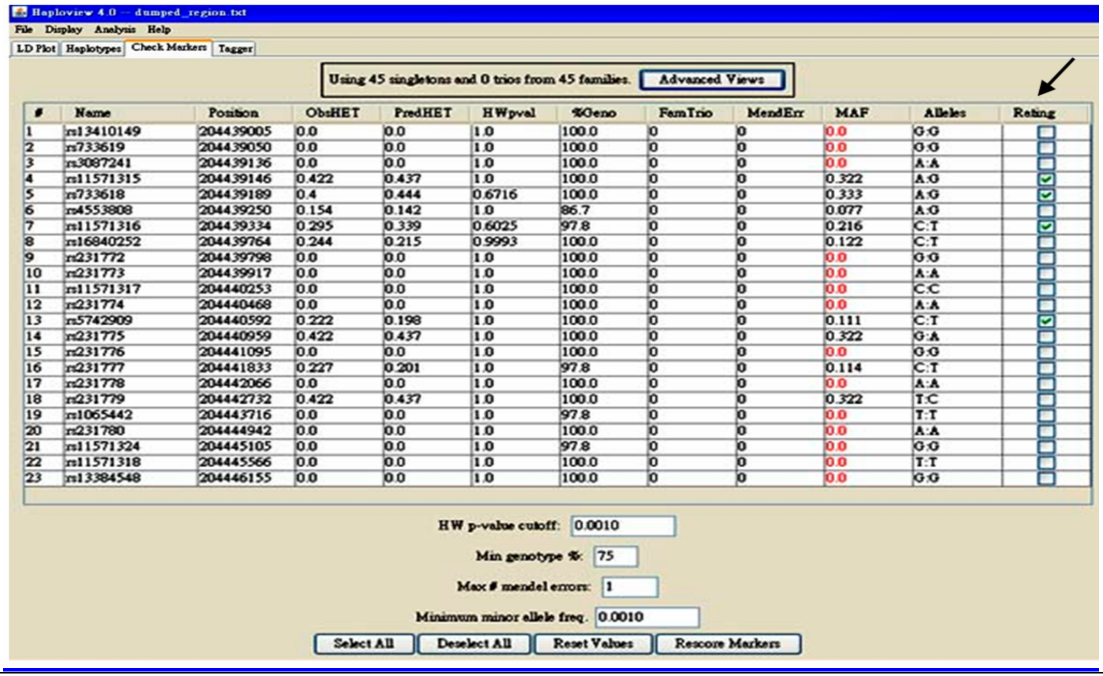

#	Name	Position	ObsHET	PredHET	HWpval	%Geno	FamTrio	MendErr	MAF	Alleles	Rating
1	rs13410149	204439005	0.0	0.0	1.0	100.0	0	0	0.0	G:G	
2	rs733619	204439050	0.0	0.0	1.0	100.0	0	0	0.0	G:G	
3	rs3087241	204439136	0.0	0.0	1.0	100.0	0	0	0.0	A:A	
4	rs11571315	204439146	0.422	0.437	1.0	100.0	0	0	0.322	A:G	✓
5	rs733618	204439189	0.4	0.444	0.6716	100.0	0	0	0.333	A:G	✓
6	rs4553808	204439250	0.154	0.142	1.0	86.7	0	0	0.077	A:G	
7	rs11571316	204439334	0.295	0.339	0.6025	97.8	0	0	0.216	C:T	✓
8	rs16840252	204439764	0.244	0.215	0.9993	100.0	0	0	0.122	C:T	
9	rs231772	204439798	0.0	0.0	1.0	100.0	0	0	0.0	G:G	
10	rs231773	204439917	0.0	0.0	1.0	100.0	0	0	0.0	A:A	
11	rs11571317	204440253	0.0	0.0	1.0	100.0	0	0	0.0	C:C	
12	rs231774	204440468	0.0	0.0	1.0	100.0	0	0	0.0	A:A	
13	rs5742909	204440592	0.222	0.198	1.0	100.0	0	0	0.111	C:T	✓
14	rs231775	204440959	0.422	0.437	1.0	100.0	0	0	0.322	G:A	
15	rs231776	204441095	0.0	0.0	1.0	100.0	0	0	0.0	G:G	
16	rs231777	204441833	0.227	0.201	1.0	97.8	0	0	0.114	C:T	
17	rs231778	204442066	0.0	0.0	1.0	100.0	0	0	0.0	A:A	
18	rs231779	204442732	0.422	0.437	1.0	100.0	0	0	0.322	T:C	
19	rs1065442	204443716	0.0	0.0	1.0	97.8	0	0	0.0	T:T	
20	rs231780	204444942	0.0	0.0	1.0	100.0	0	0	0.0	A:A	
21	rs11571324	204445105	0.0	0.0	1.0	97.8	0	0	0.0	G:G	
22	rs11571318	204445566	0.0	0.0	1.0	100.0	0	0	0.0	T:T	
23	rs13384548	204446155	0.0	0.0	1.0	100.0	0	0	0.0	G:G	

Fig. 17. Manual selection from range-based SNP retrieval of HapMap using Haploview. If the LD analysis for only some of the SNP panel is interested, it should be firstly input the range covering all SNPs, e.g., Chr2:204,438,754..204,446,928. Second, users have to manually select interested SNPs (indicated by arrow line). Finally, the LD analysis for these selected SNPs is able to perform by Haploview. Examples for selected rsIDs are followed: rs11571315, rs733618, rs5742909, and rs11571316.

The patterns of 2D-LD plot for LD$_2$SNPing (in the example of HapMap-CEU) and Haploview are completely matched as shown in Fig. 18A and Fig. 18B, respectively.

A

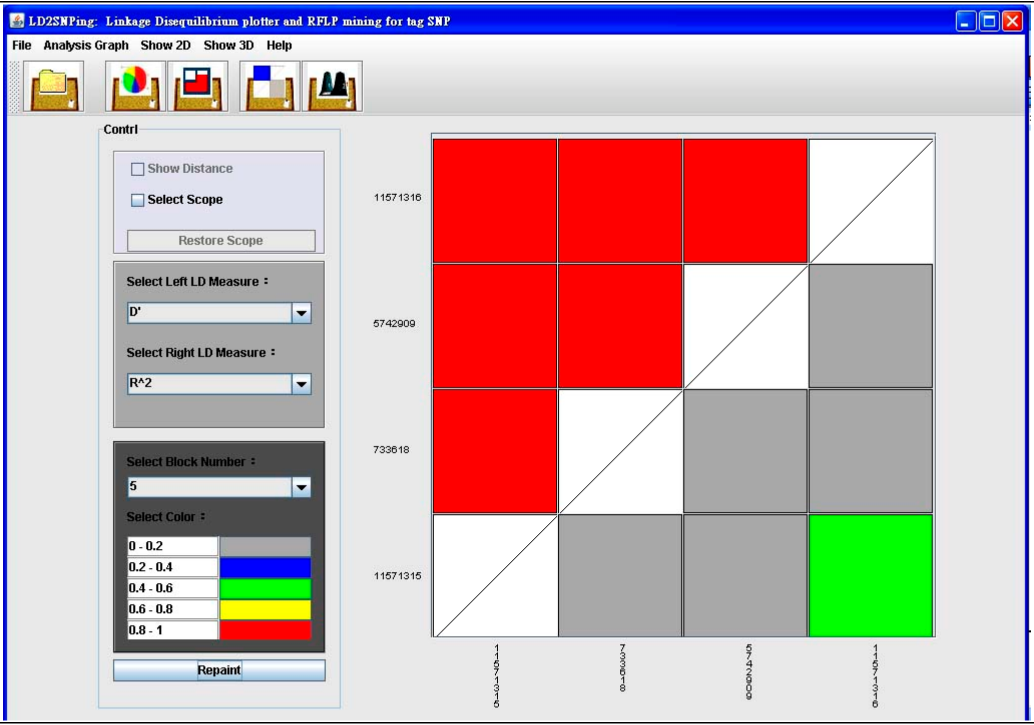

B

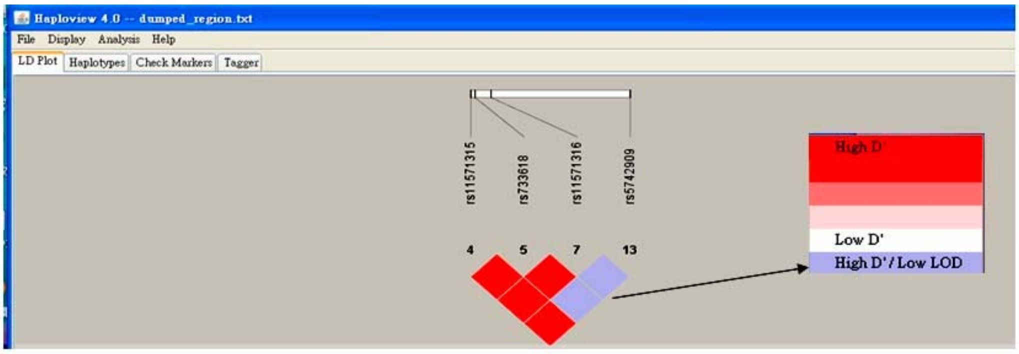

L1	L2	D'	LOD	r^2	CIlow	CIhi	Dist	T-int
rs11571315	rs733618	1	2.84	0.103	0.48	1	43	18.37
rs11571315	rs11571316	1	13.44	0.507	0.85	1	188	-
rs11571315	rs5742909	1	2.09	0.061	0.38	1	1446	-
rs733618	rs11571316	1	1.99	0.057	0.36	1	145	17.82
rs733618	rs5742909	1	0.3	0.006	0.06	0.98	1403	-
rs11571316	rs5742909	1	2.29	0.084	0.42	1	1258	4.68

Fig. 18. Comparison of four rsID# input and its 2D LD plot in LD$_2$SNPing *vs.* Haploview. Four rsID# is rs11571315, rs733618, rs5742909, and rs11571316, which is belonging to CTLA4 gene located at chromosome 2. (A) and (B) are the 2D-LD plots for LD$_2$SNPing (Fig. 16) and Haploview (Fig. 17), respectively. The *D'* for all pair-wised SNPs in Haploview is 1. The r^2 for LD$_2$SNPing and Haploview is also matched.

4.3.4. rsID# and ssID# searching for SNP frequency information

For LD calculation to specific population, the selection of specific ssID# may be helpful to analyze the population-based association studies. Different records from different submitters for the same SNP rsID# are given with the ssID# (Fig. 19, rs2078486) (http://www.ncbi.nlm.nih.gov/SNP/snp_ref.cgi?rs=2078486).

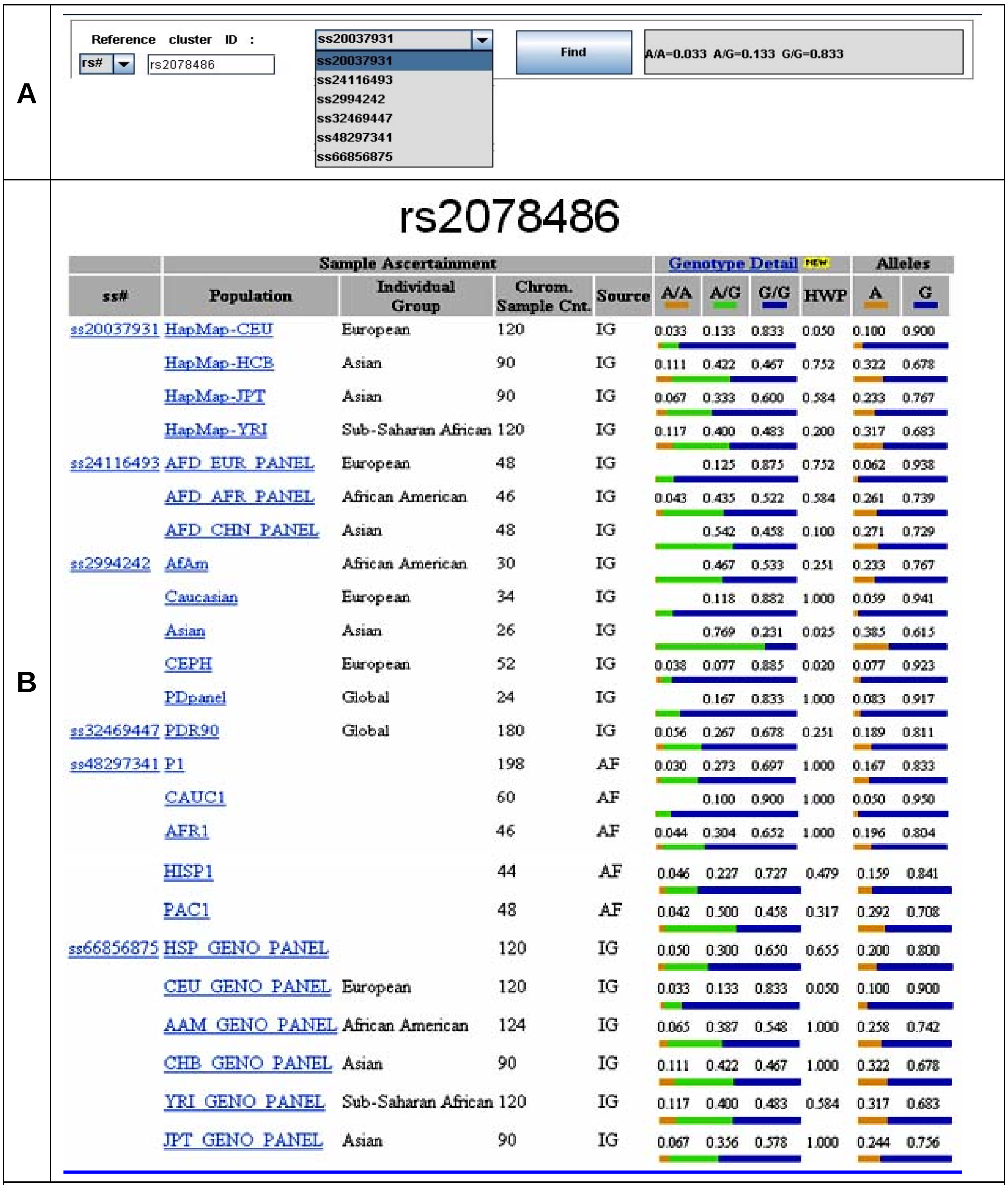

ss#	Population	Individual Group	Chrom. Sample Cnt.	Source	A/A	A/G	G/G	HWP	A	G
	Sample Ascertainment				Genotype Detail NEW				Alleles	
ss20037931	HapMap-CEU	European	120	IG	0.033	0.133	0.833	0.050	0.100	0.900
	HapMap-HCB	Asian	90	IG	0.111	0.422	0.467	0.752	0.322	0.678
	HapMap-JPT	Asian	90	IG	0.067	0.333	0.600	0.584	0.233	0.767
	HapMap-YRI	Sub-Saharan African	120	IG	0.117	0.400	0.483	0.200	0.317	0.683
ss24116493	AFD_EUR_PANEL	European	48	IG		0.125	0.875	0.752	0.062	0.938
	AFD_AFR_PANEL	African American	46	IG	0.043	0.435	0.522	0.584	0.261	0.739
	AFD_CHN_PANEL	Asian	48	IG		0.542	0.458	0.100	0.271	0.729
ss2994242	AfAm	African American	30	IG		0.467	0.533	0.251	0.233	0.767
	Caucasian	European	34	IG		0.118	0.882	1.000	0.059	0.941
	Asian	Asian	26	IG		0.769	0.231	0.025	0.385	0.615
	CEPH	European	52	IG	0.038	0.077	0.885	0.020	0.077	0.923
	PDpanel	Global	24	IG		0.167	0.833	1.000	0.083	0.917
ss32469447	PDR90	Global	180	IG	0.056	0.267	0.678	0.251	0.189	0.811
ss48297341	P1		198	AF	0.030	0.273	0.697	1.000	0.167	0.833
	CAUC1		60	AF		0.100	0.900	1.000	0.050	0.950
	AFR1		46	AF	0.044	0.304	0.652	1.000	0.196	0.804
	HISP1		44	AF	0.046	0.227	0.727	0.479	0.159	0.841
	PAC1		48	AF	0.042	0.500	0.458	0.317	0.292	0.708
ss66856875	HSP_GENO_PANEL		120	IG	0.050	0.300	0.650	0.655	0.200	0.800
	CEU_GENO_PANEL	European	120	IG	0.033	0.133	0.833	0.050	0.100	0.900
	AAM_GENO_PANEL	African American	124	IG	0.065	0.387	0.548	1.000	0.258	0.742
	CHB_GENO_PANEL	Asian	90	IG	0.111	0.422	0.467	1.000	0.322	0.678
	YRI_GENO_PANEL	Sub-Saharan African	120	IG	0.117	0.400	0.483	0.584	0.317	0.683
	JPT_GENO_PANEL	Asian	90	IG	0.067	0.356	0.578	1.000	0.244	0.756

Fig. 19. Many ssID# for single rsID#. The SNP rs2078486 is used as an example. (A) ssID# is selectable from pull-down window. (B) The SNP frequency for each ssID# in dbSNP of NCBI. The SNP frequency for each ssID# in LD$_2$SNPing is the same as dbSNP of NCBI (not shown, please check it on-line using LD$_2$SNPing). Although the genotype frequency data are available, most of them are unable to retrieve the genotype raw data for LD measurement except the dataset of HapMap series (ss# 20037931).

Each ssID# may be derived from different populations for single rsID# (like Fig. 20, rs41446050). The ssID# for corresponding rsID# is selective using pull-down window. The SNP frequency of many ssID# for rs41446050 in LD$_2$SNPing is the same as in dbSNP of NCBI (http://www.ncbi.nlm.nih.gov/SNP/snp_ref.cgi?rs=41446050).

Although the genotype frequency data are available, all of them are unable to retrieve the genotype raw data for LD measurement in this case. Using the genotype frequency, the LD$_2$SNPing randomly generates the simulated genotype data to calculate the simulated LD analysis when there is not HapMap raw data.

Since they have the genotype frequency, a simulation for 100 randomized genotypes was computationally generated to fit their genotype frequency. Different times to perform the simulation may lead to the different genotype orders in a population. To provide the more reliable LD value, we designed ten different simulations to perform their LD analysis and the average of these LD values was provided in LD$_2$SNPing. Although the possible linkage between different SNPs within the same individual is ignored, this method provides the LD evaluation for SNPs with genotype frequencies alone. <u>However, this kind of evaluation is not suggested if the HapMap is available for the selected SNP ID. SNP information from different populations was not suggested to perform the LD calculation.</u>

A

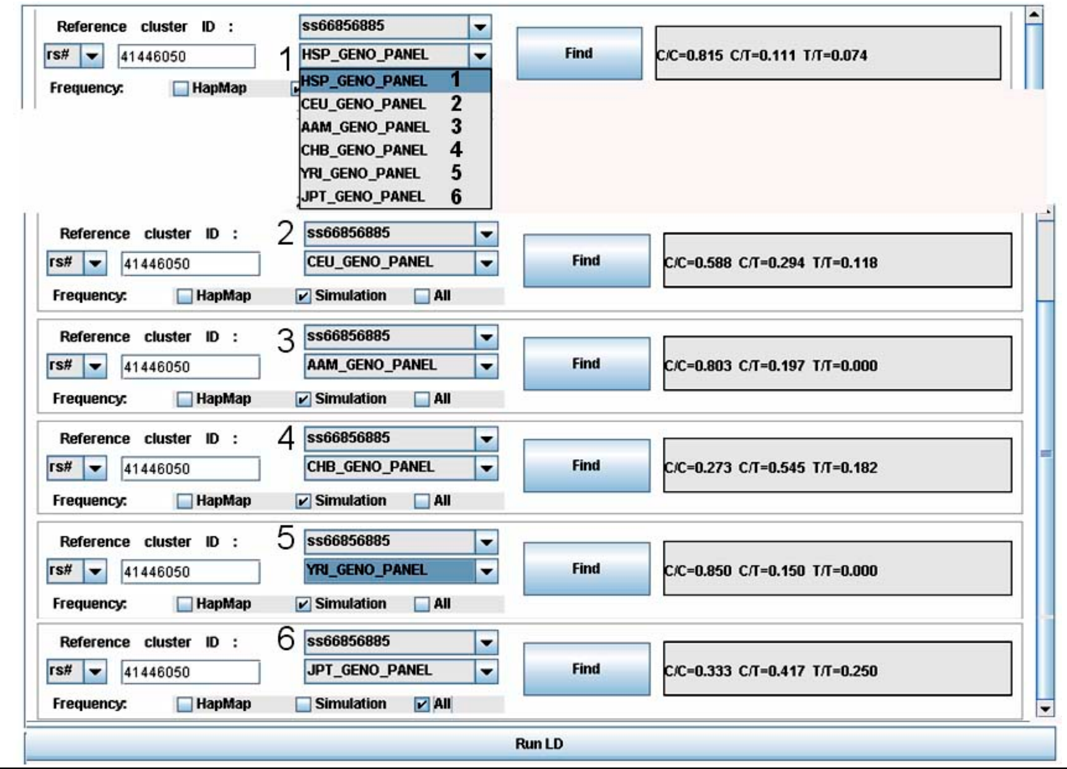

B

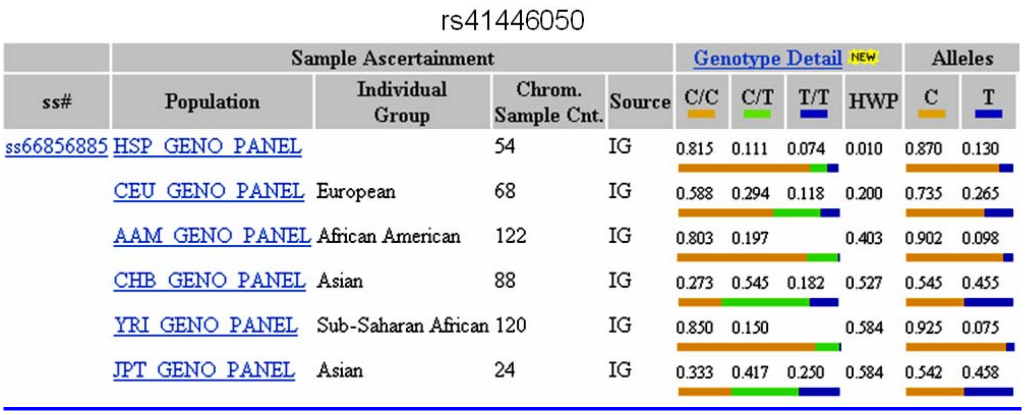

rs41446050

	Sample Ascertainment				Genotype Detail NEW				Alleles	
ss#	Population	Individual Group	Chrom. Sample Cnt.	Source	C/C	C/T	T/T	HWP	C	T
ss66856885	HSP_GENO_PANEL		54	IG	0.815	0.111	0.074	0.010	0.870	0.130
	CEU_GENO_PANEL	European	68	IG	0.588	0.294	0.118	0.200	0.735	0.265
	AAM_GENO_PANEL	African American	122	IG	0.803	0.197		0.403	0.902	0.098
	CHB_GENO_PANEL	Asian	88	IG	0.273	0.545	0.182	0.527	0.545	0.455
	YRI_GENO_PANEL	Sub-Saharan African	120	IG	0.850	0.150		0.584	0.925	0.075
	JPT_GENO_PANEL	Asian	24	IG	0.333	0.417	0.250	0.584	0.542	0.458

Fig. 20. Single ssID# from different populations for single rsID#. rs41446050 (ss66856885) is used as an example. (A) Single ssID# of different populations is selectable from the pull-down window. (B) The SNP frequency of each population of single ssID# in dbSNP of NCBI. The SNP frequency for each population for single ssID# in LD$_2$SNPing is the same as dbSNP of NCBI.

4.3.5. LD calculation and 2D visualization

4.3.5.1. Eight functions in control panel of 2D-LD plot

After dataset input, the LD$_2$SNPing performs of Hardy-Weinberg Equilibrium, Expectation Maximization (EM) algorithm and linkage disequilibrium (LD) calculation for LD-related measurement, such as *D* (the difference in proportions), *D'* (the Lewontin's *D*), r^2 (the square of the standardized measures), δ (the Delvin's population attributable factor), *Q* (the Yule's *Q*) and ρ. All these formulas are supplemented in the end of this user manual (*see* Appendix).

In LD$_2$SNPing, eight functions are provided in control panel of 2D-LD plot as shown in Fig. 21. They are described in detail later (after Fig. 22).

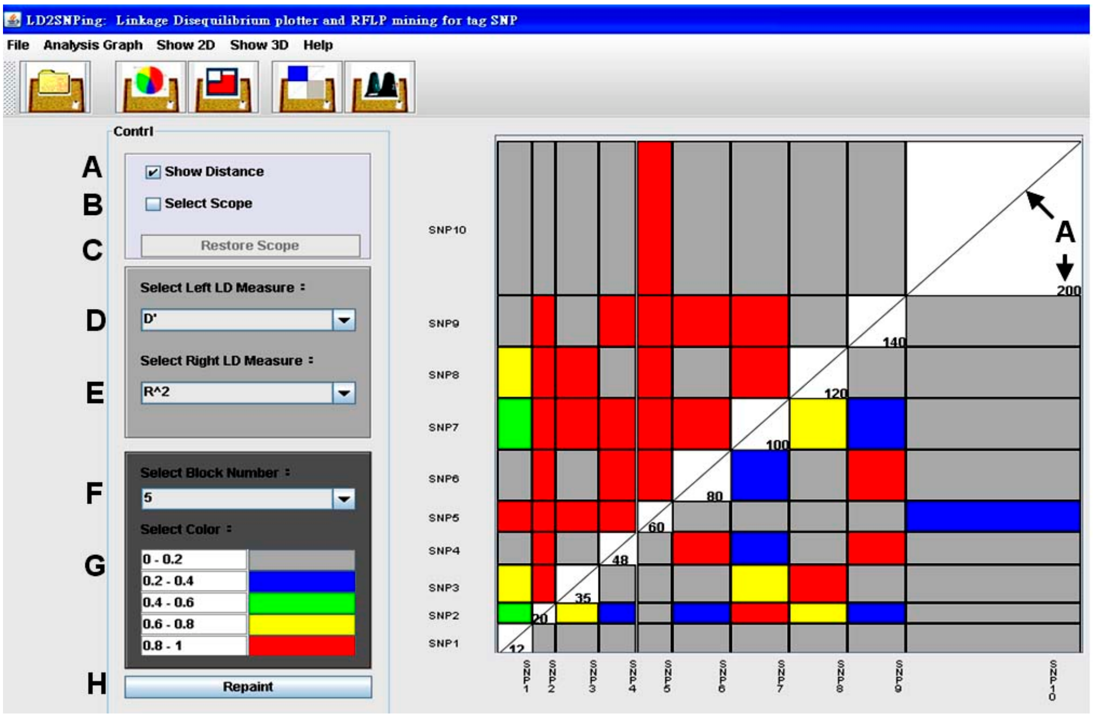

Fig. 21. Eight functions in control panel of 2D-LD plot. Data source: SNP10-distance.xls in *example file folder* of LD$_2$SNPing. (A) Option to show or hide the distance. (B) Scope selection of the 2D-LD plot. (C) Restore the action of scope selection by (B). (D) Pull-down selection of the left (vertebrate axis) LD measure. (E) Pull-down selection of the right (horizontal axis) LD measure. (F) Select the block number to be numbers of colors. (G) Color selection by user-defined colors. (H) Repaint the 2D-LD profile.

4.3.5.2. Showing/omitting the distance between SNPs

In the Fig. 22, LD$_2$SNPing demonstrates that distance between SNPs is optional to show or hide. The distance value shown in SNPn is the distance between SNPn to zero point.

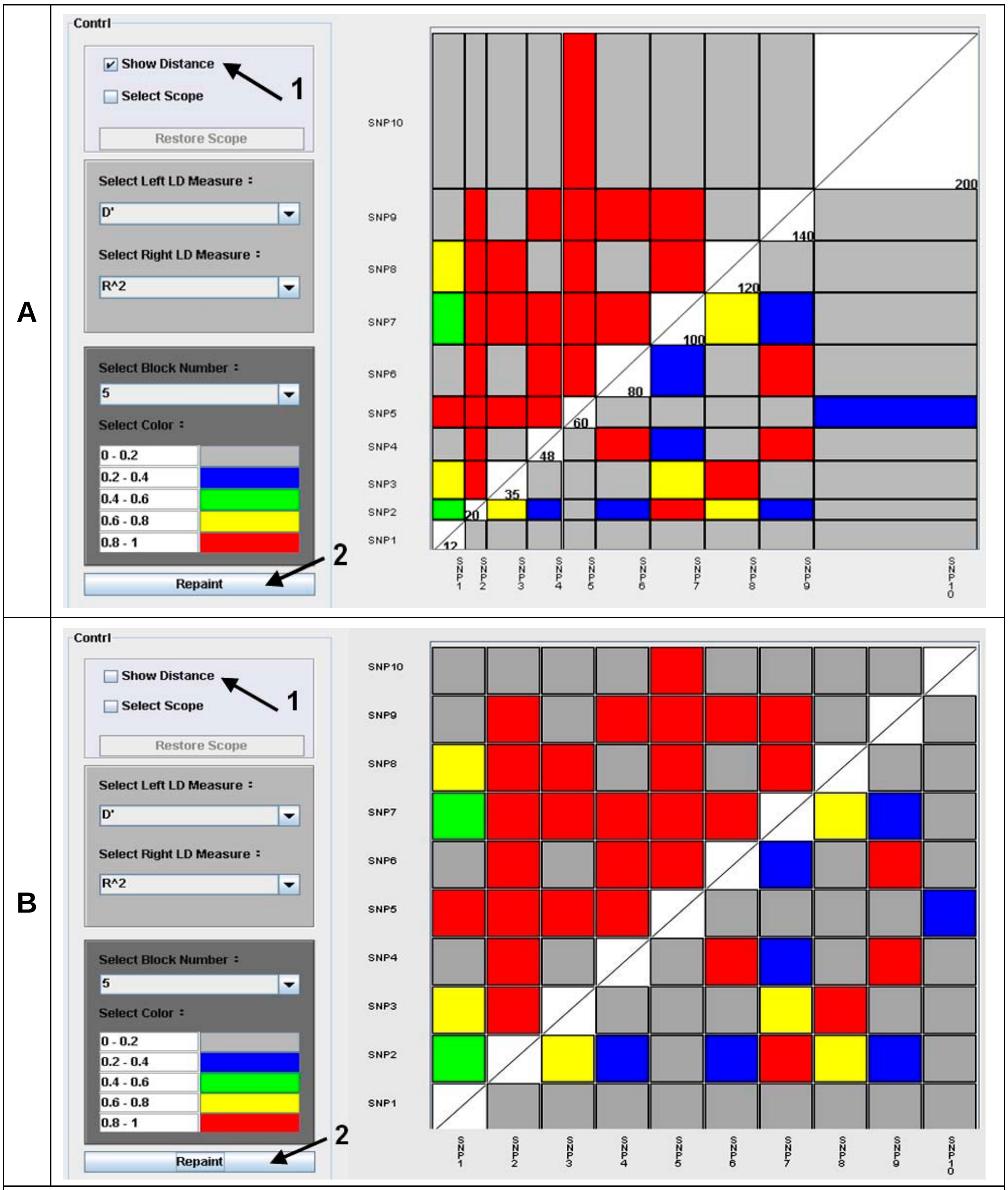

Fig. 22. Distances between SNPs are able to be visualized or omit. If the input data contain distance between SNPs (e.g., SNP10-distance.xls in *example file folder* of LD$_2$SNPing), users can chose to show (A) or omit (B) it. (A) Clicking the arrow 1 to un-select the “show distance” and clicking the arrow 2 (repaint), users will see the result as shown in Fig. 22B. (B) Clicking the arrow 1 to re-select the “show distance” and clicking the arrow 2, users will see the result as shown in Fig. 22A. Both (A) and (B) are interchangeable. Animation

4.3.5.3. Scope selection from large to small area of 2D-LD plot

Scope control is designed to narrow-down the SNP number for LD analysis if the huge SNP data is evaluated. As shown in Fig. 23, users can “zoom in” to select SNP within small region and it is reversible.

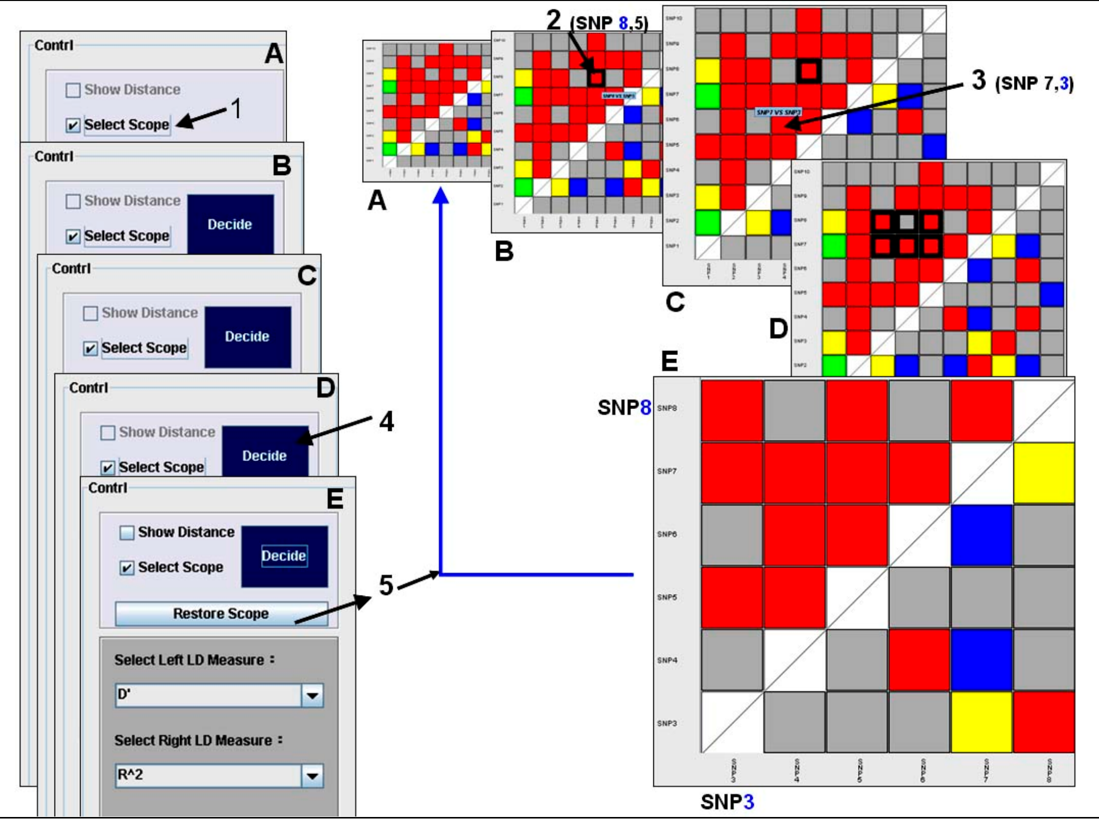

Fig. 23. Scope selection from large to small area of 2D-LD plot. This function is similar to zoom-in. 2D-LD plots (e.g., SNP10-distance.xls in *example file folder* of LD$_2$SNPing) in right side are the results for the control panels A-E in parallel, respectively. (A) Clicking the arrow 1, users can start to test the function of scope selection. (B, C) Both of B and C are clicked at the corner for selected region as indicated to arrow 2 and arrow 3 for SNP8 *vs.* SNP5 and SNP7 *vs.* SNP3, respectively. Then, the range from SNP max.# to min.# is selected. (D) The selected region is black background subsequently. Once clicking the arrow 4, the result is shown in (E) to represent the scope with narrow down manner. Users may return to the original plot by clicking the arrow 5 (restore scope) as indicated by blue arrow line. Animation

4.3.5.4. Visualization of parameter changing of 2D-LD plot

In Fig. 24, the parameter changing for drawing the 2D-LD plot is presented. Left and right LD measures are changed and successfully re-drawn. Many parameters are accepted to change as needed.

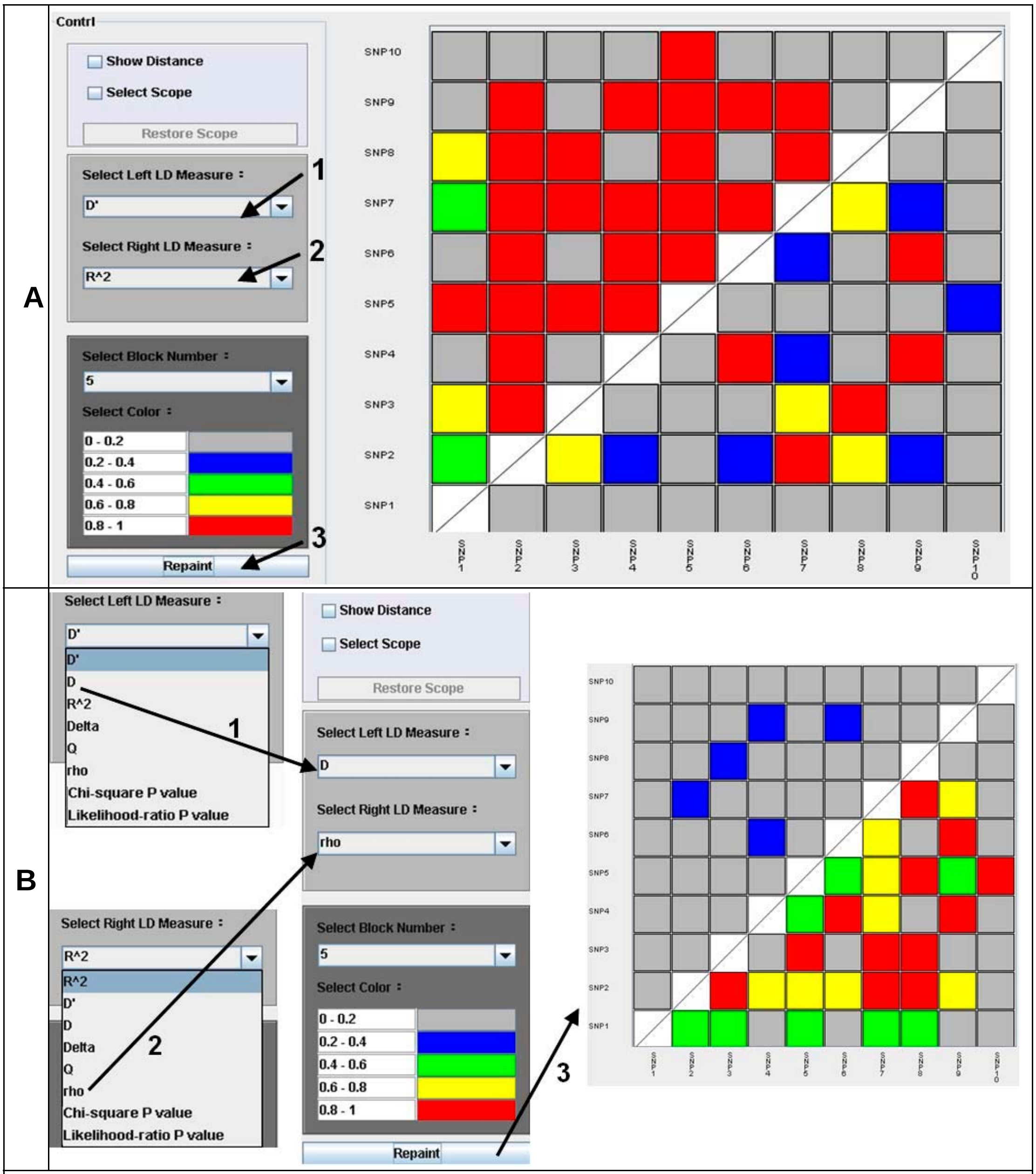

Fig. 24. Visualization of parameter changing of 2D-LD plot. SNP10-distance.xls in example file folder of LD$_2$SNPing is used as an example. (A) The parameters in 2D-LD plot. Arrows 1 and 2 indicate the vertical (*D'*) and horizontal (-logP) axes, respectively. Both of them are the pull-down windows for selection. Once selection, users can click the "repaint" (arrow 3) to view the re-drawn plot. (B) Demonstration of parameter changing of 2D-LD plot. Arrows 1 and 2 indicate the changing of *D'* to *D*, r^2 to ρ, respectively. After clicking the repaint (arrow 3), the new 2D-LD plot is shown. Animation

4.3.5.5. Color setting for 2D-LD display graphics

In the Fig. 25, arrow 1 indicates the function of select color blocks and the default number is five blocks. The color gradient for the LD measure value raining 0.0-0.2 for gray, 0.2-0.4 for blue, 0.4-0.6 for green, 0.6-0.8 for yellow, and 0.8-1.0 for red is default setting. Users can choose the block number and the color display if needed (please see detail in Figs. 26-29).

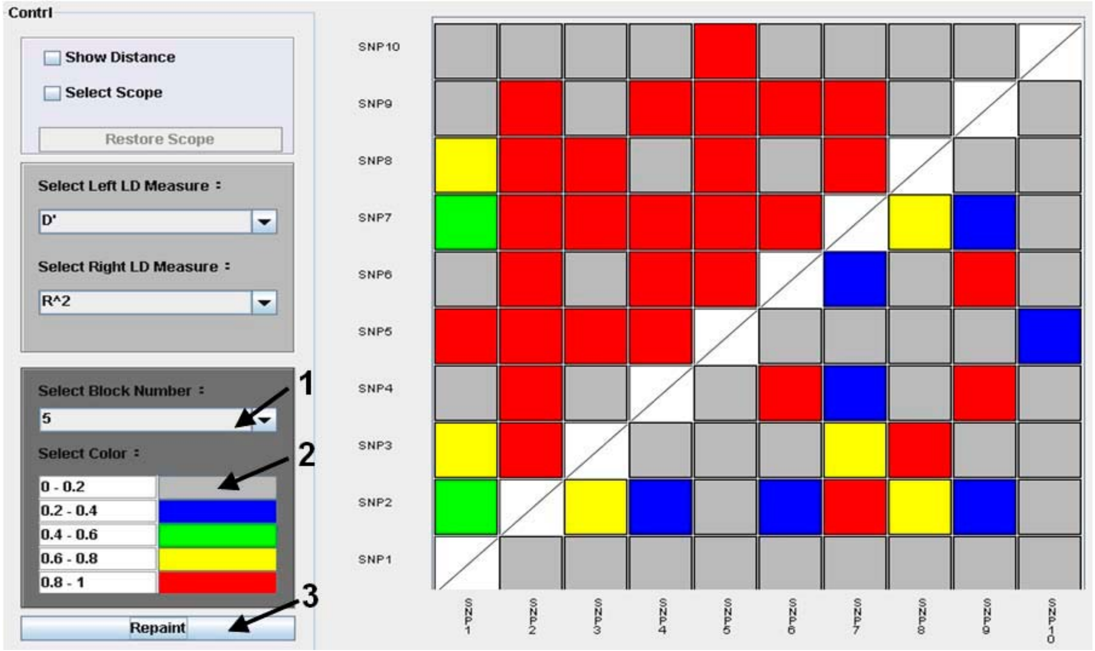

Fig. 25. Color setting for 2D-LD display graphics-1. SNP10-distance.xls in *example file folder* of LD$_2$SNPing is used as an example. Arrow 1 indicates the pull-down window to "select block number" for colors, e. g., 5 indicates the 5 colors, such as gray, blue, green, yellow and red colors. Clicking the pull-down window (arrow 1) is able to select different block number of colors. Clicking any colors in the window of "select color" (arrow 2) one-by-one, it allows the users to change color for each clicking. After color setting, users can click the "repaint" (arrow 3) to get the re-colored 2D-LD plot. *Please see next figures for detail steps (Color setting for 2D-LD display graphics-2 to 5).* Animation (Figs. 25-29 in one animation)

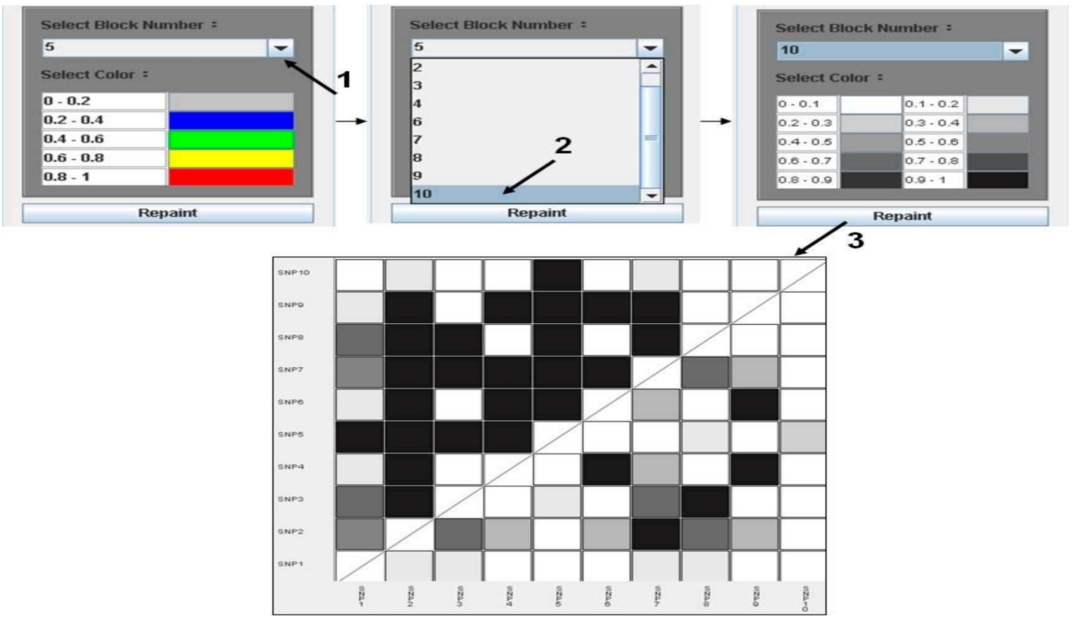

Fig. 26. Color setting for 2D-LD display graphics-2. SNP10-distance.xls in *example file folder* of LD$_2$SNPing is used as an example. Step 1: Click the pull-down window (arrow 1). Step 2: Select the needed block numbers (e.g., 10 in arrow 2). The default color setting is gray-scale. If there is not necessary to change, then go to next step. *If users want to change each color, please see Fig. 27 for detail.* Step 3: Click the "repaint" box (arrow 3). The color resetting is completely finished. Animation (Figs. 25-29 in one animation)

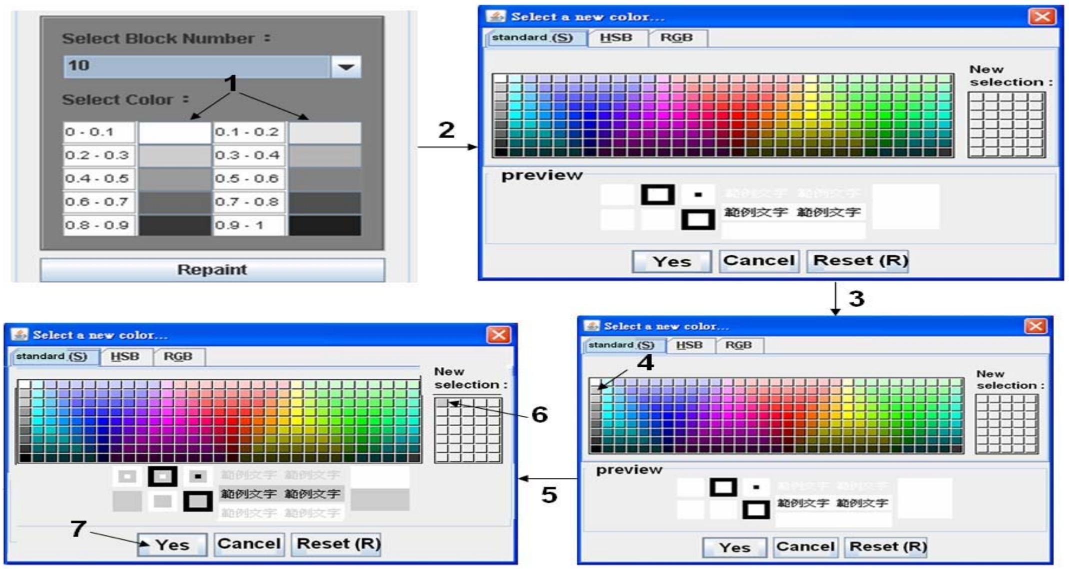

Fig. 27. Color setting for 2D-LD display graphics-3. The steps to change each color in each block are followed. Step 1: Click the needed box for color change (arrow 1). Step 2: The color panel for a new color selection appears (arrow 2). Here the standard color panel is provided. Steps 3 and 4: Clicking the desired color box (arrow 3) such as the gray color indicated by arrow 4. Step 5: The new selection color appears in the blank color panel in the right side as indicated by arrow 6. Finally, clicking the "Yes" box indicated by arrow 7 to confirm the color setting. Animation (Figs. 25-29 in one animation)

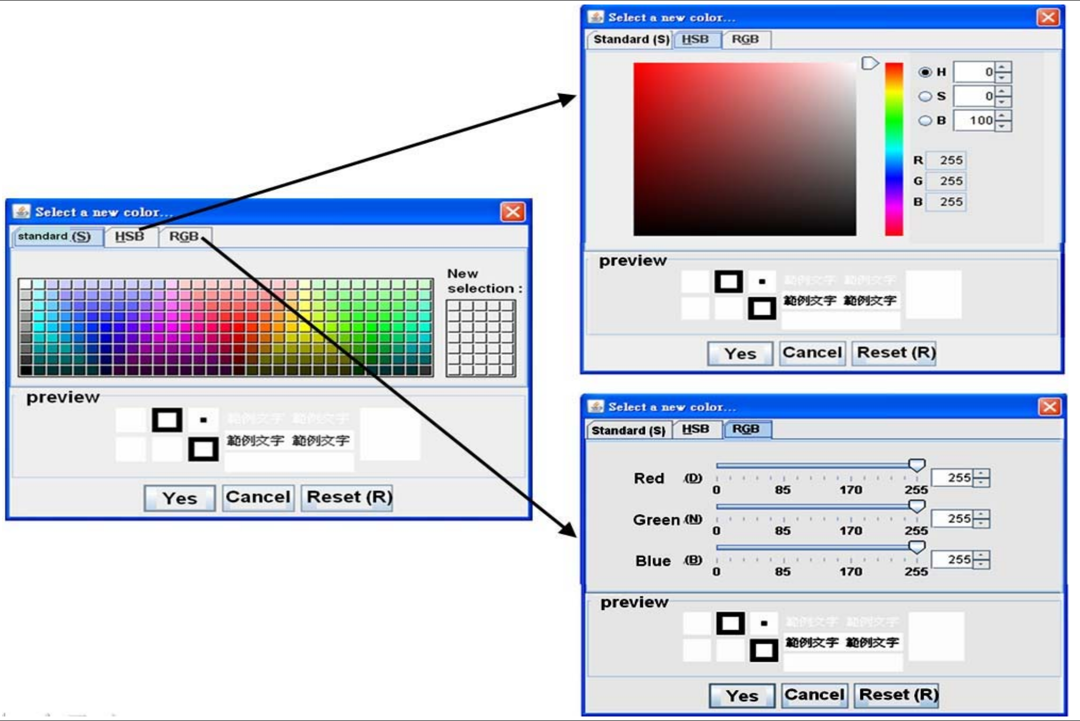

Fig. 28. Color setting for 2D-LD display graphics-4. In addition to the standard color panel as shown in Fig. 27, the LD$_2$SNPing also provides other two types of color panel such as HSB and RGB which are shown in the up and down of the right side, respectively. Animation (Figs. 25-29 in one animation)

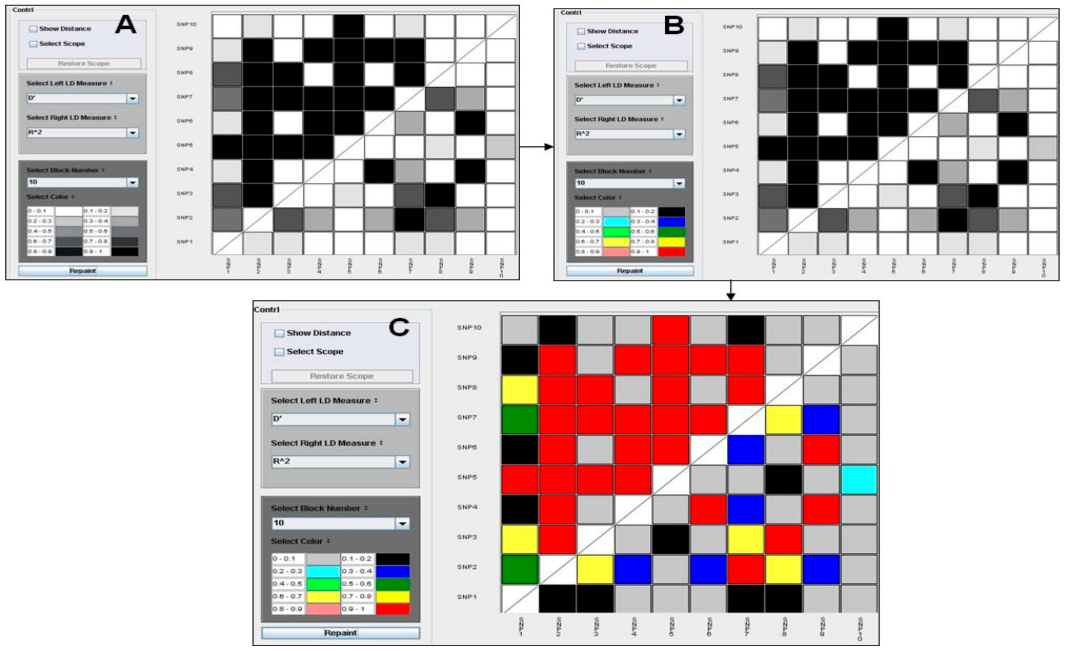

Fig. 29. Color setting for 2D-LD display graphics-5. SNP10-distance.xls in *example file folder* of LD$_2$SNPing is used as an example. Here, we provide the summary for changing colors as described in Figs. 25-28. (A) The original color setting in Fig. 26. (B) Changing the color setting one-by-one and the outcome is shown in (C) after clicking the "repaint". Animation (Figs. 25-29 in one animation)

4.3.5.6. LD measure text data for all SNPs

The LD$_2$SNPing provides the both the visualization for LD plot and the text data for LD related information. In Fig. 30, the immediate response to check the specific pair-wised SNPs is demonstrated. If users want to view the LD related information for all SNPs within this plot, please see detail in Figs. 31 and 32.

A

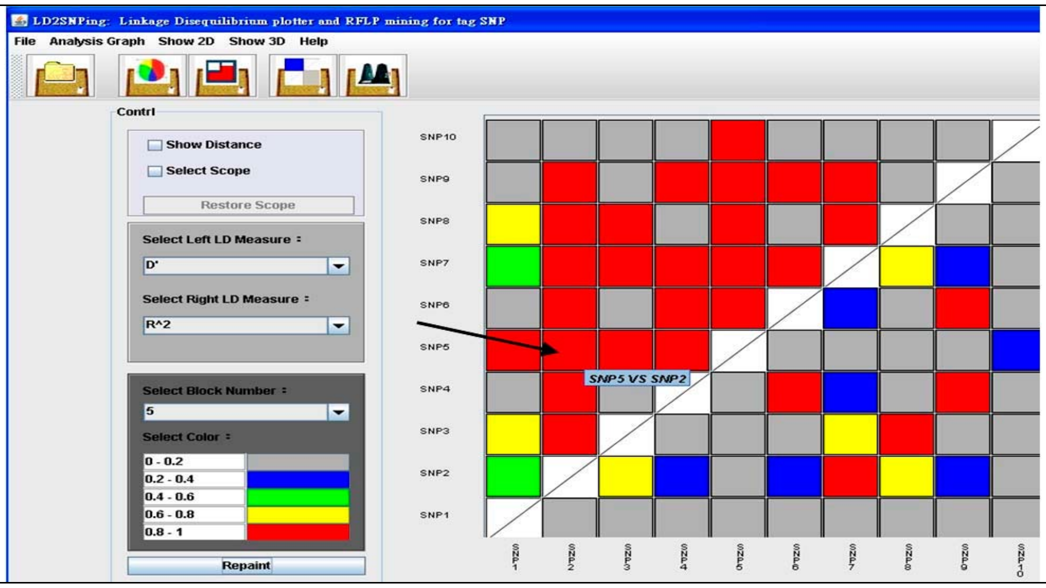

B

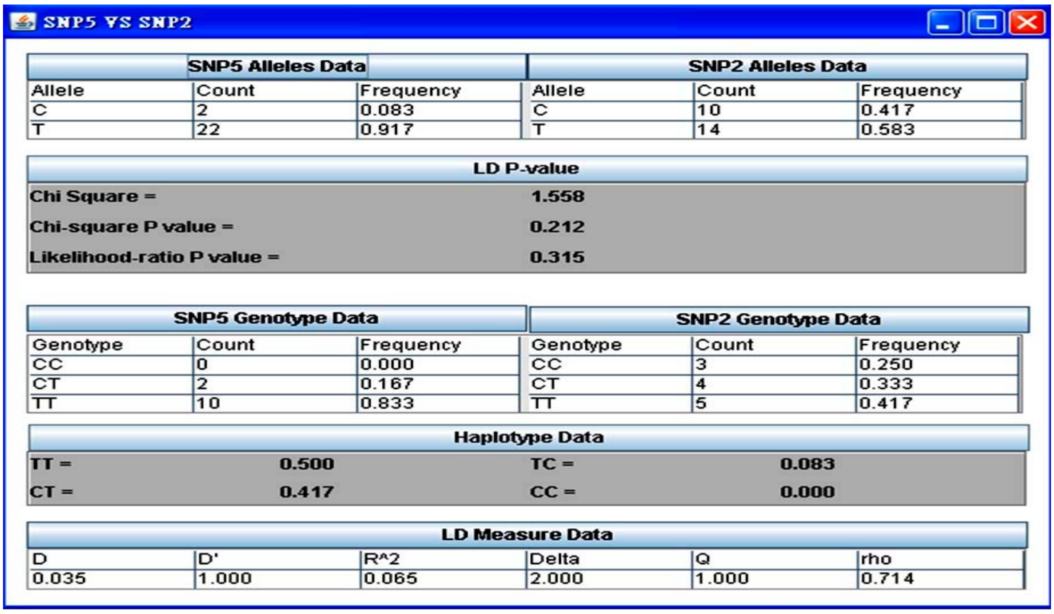

Fig. 30. LD measure text data for all pair-wised SNPs. SNP10-distance.xls in *example file folder* of LD$_2$SNPing is used as an example. (A) Visualization of 2D-LD plot spontaneously provides the text data for LD related information by clicking. For example, moving-over the box for SNP5 *vs.* SNP2 as indicated by arrow provides the information. (B) Once clicking the interested box, the text data for LD related information is provided. *Note!* The likelihood-ratio P value is dependent on the number of randomization and it shows minor variation for likelihood-ratio P value at different times. Please see the formula in the Appendix at the end of the user manual. Animation

4.3.5.7. Save text file for output LD measure data

The LD$_2$SNPing provides the output for the LD related text data of all SNPs within this plot such as Fig. 31. In Fig. 32, the steps to save the text data for LD plot are demonstrated. The output for LD graph is described later (Fig. 33).

A

B

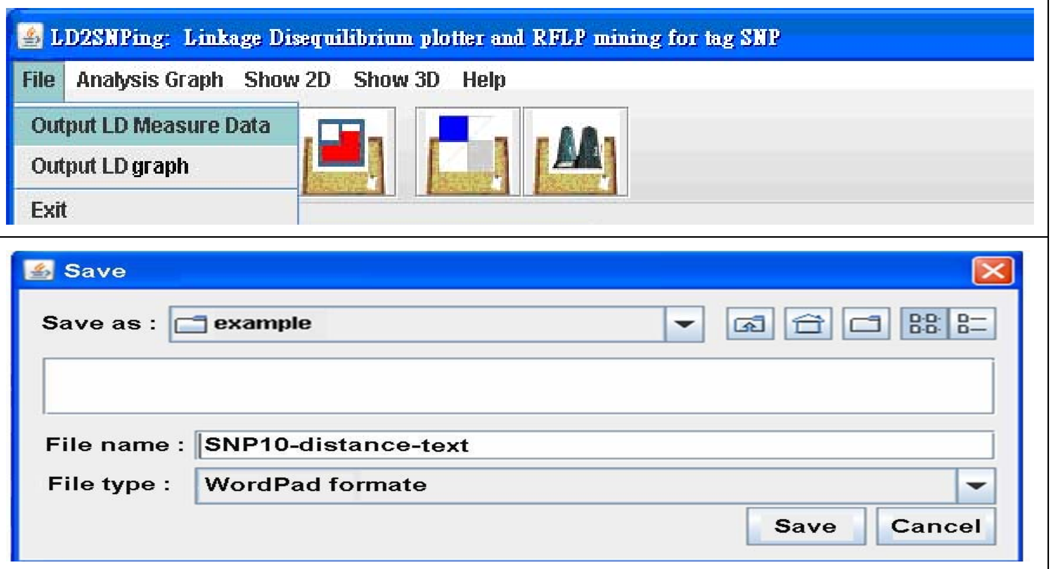

Fig. 31. Output information for saving file. SNP10-distance.xls in *example file folder* of LD$_2$SNPing is used as an example. (A) Clicking the "output LD measure Data", users are able to download and save all the text data (B). The location for saving file is changeable if needed. Animation

4.3.5.8. Open text file for output LD measure data

In Fig. 32, all text data for LD related information are re-opened and demonstrated.

A

SNP10-distance-text.txt - WordPad

檔案(F) 編輯(E) 檢視(V) 插入(I) 格式(O) 說明(H)

***** LD Measure Data *****

LD Measure = D value

	SNP1	SNP2	SNP3	SNP4	SNP5	SNP6	SNP7	SNP8	SNP9	SNP10
SNP1		0.087	0.092	0.028	0.024	0.028	0.087	0.092	0.028	0.010
SNP2	0.087		0.191	0.139	0.035	0.139	0.243	0.191	0.139	0.021
SNP3	0.092	0.191		0.014	0.045	0.014	0.191	0.248	0.014	0.010
SNP4	0.028	0.139	0.014		0.028	0.222	0.139	0.014	0.222	0.000
SNP5	0.024	0.035	0.045	0.028		0.028	0.035	0.045	0.028	0.062
SNP6	0.028	0.139	0.014	0.222	0.028		0.139	0.014	0.222	0.000
SNP7	0.087	0.243	0.191	0.139	0.035	0.139		0.191	0.139	0.021
SNP8	0.092	0.191	0.248	0.014	0.045	0.014	0.191		0.014	0.010
SNP9	0.028	0.139	0.014	0.222	0.028	0.222	0.139	0.014		0.000
SNP10	0.010	0.021	0.010	0.000	0.062	0.000	0.021	0.010	0.000	

LD Measure = D' value

	SNP1	SNP2	SNP3	SNP4	SNP5	SNP6	SNP7	SNP8	SNP9	SNP10
SNP1		0.510	0.688	0.143	1.000	0.143	0.510	0.688	0.143	0.059
SNP2	0.510		1.000	1.000	1.000	1.000	1.000	1.000	1.000	0.200
SNP3	0.688	1.000		0.077	1.000	0.077	1.000	1.000	0.077	0.077
SNP4	0.143	1.000	0.077		1.000	1.000	1.000	0.077	1.000	0.000
SNP5	1.000	1.000	1.000	1.000		1.000	1.000	1.000	1.000	1.000
SNP6	0.143	1.000	0.077	1.000	1.000		1.000	0.077	1.000	0.000
SNP7	0.510	1.000	1.000	1.000	1.000	1.000		1.000	1.000	0.200
SNP8	0.688	1.000	1.000	0.077	1.000	0.077	1.000		0.077	0.077
SNP9	0.143	1.000	0.077	1.000	1.000	1.000	1.000	0.077		0.000
SNP10	0.059	0.200	0.077	0.000	1.000	0.000	0.200	0.077	0.000	

B

LD Measure = r^2 value

	SNP1	SNP2	SNP3	SNP4	SNP5	SNP6	SNP7	SNP8	SNP9	SNP10
SNP1		0.150	0.165	0.017	0.037	0.017	0.150	0.165	0.017	0.003
SNP2	0.150		0.604	0.357	0.065	0.357	1.000	0.604	0.357	0.010
SNP3	0.165	0.604		0.003	0.107	0.003	0.604	1.000	0.003	0.002
SNP4	0.017	0.357	0.003		0.045	1.000	0.357	0.003	1.000	0.000
SNP5	0.037	0.065	0.107	0.045		0.045	0.065	0.107	0.045	0.273
SNP6	0.017	0.357	0.003	1.000	0.045		0.357	0.003	1.000	0.000
SNP7	0.150	1.000	0.604	0.357	0.065	0.357		0.604	0.357	0.010
SNP8	0.165	0.604	1.000	0.003	0.107	0.003	0.604		0.003	0.002
SNP9	0.017	0.357	0.003	1.000	0.045	1.000	0.357	0.003		0.000
SNP10	0.003	0.010	0.002	0.000	0.273	0.000	0.010	0.002	0.000	

LD Measure = Delta value

	SNP1	SNP2	SNP3	SNP4	SNP5	SNP6	SNP7	SNP8	SNP9	SNP10
SNP1		0.588	3.118	0.314	2.000	0.314	0.588	3.118	0.314	0.167
SNP2	0.588		2.000	2.000	2.000	2.000	1.000	2.000	2.000	0.333
SNP3	3.118	2.000		0.125	0.591	0.125	2.000	1.000	0.125	0.111
SNP4	0.314	2.000	0.125		2.000	1.000	2.000	0.125	1.000	0.000
SNP5	2.000	2.000	0.591	2.000		2.000	2.000	0.591	2.000	0.818
SNP6	0.314	2.000	0.125	1.000	2.000		2.000	0.125	1.000	0.000
SNP7	0.588	1.000	2.000	2.000	2.000	2.000		2.000	2.000	0.333
SNP8	3.118	2.000	1.000	0.125	0.591	0.125	2.000		0.125	0.111
SNP9	0.314	2.000	0.125	1.000	2.000	1.000	2.000	0.125		0.000
SNP10	0.167	0.333	0.111	0.000	0.818	0.000	0.333	0.111	0.000	

C

LD Measure = Q value

	SNP1	SNP2	SNP3	SNP4	SNP5	SNP6	SNP7	SNP8	SNP9	SNP10
SNP1		0.714	0.791	0.286	1.000	0.286	0.714	0.791	0.286	0.130
SNP2	0.714		1.000	1.000	1.000	1.000	1.000	1.000	1.000	0.231
SNP3	0.791	1.000		0.125	1.000	0.125	1.000	1.000	0.125	0.111
SNP4	0.286	1.000	0.125		1.000	1.000	1.000	0.125	1.000	0.000
SNP5	1.000	1.000	1.000	1.000		1.000	1.000	1.000	1.000	1.000
SNP6	0.286	1.000	0.125	1.000	1.000		1.000	0.125	1.000	0.000
SNP7	0.714	1.000	1.000	1.000	1.000	1.000		1.000	1.000	0.231
SNP8	0.791	1.000	1.000	0.125	1.000	0.125	1.000		0.125	0.111
SNP9	0.286	1.000	0.125	1.000	1.000	1.000	1.000	0.125		0.000
SNP10	0.130	0.231	0.111	0.000	1.000	0.000	0.231	0.111	0.000	

LD Measure = rho value

	SNP1	SNP2	SNP3	SNP4	SNP5	SNP6	SNP7	SNP8	SNP9	SNP10
SNP1		0.510	0.582	0.143	0.412	0.143	0.510	0.582	0.143	0.059
SNP2	0.510		0.846	0.714	0.714	0.714	1.000	0.846	0.714	0.143
SNP3	0.582	0.846		0.077	1.000	0.077	0.846	1.000	0.077	0.077
SNP4	0.143	0.714	0.077		0.500	1.000	0.714	0.077	1.000	0.000
SNP5	0.412	0.714	1.000	0.500		0.500	0.714	1.000	0.500	1.000
SNP6	0.143	0.714	0.077	1.000	0.500		0.714	0.077	1.000	0.000
SNP7	0.510	1.000	0.846	0.714	0.714	0.714		0.846	0.714	0.143
SNP8	0.582	0.846	1.000	0.077	1.000	0.077	0.846		0.077	0.077
SNP9	0.143	0.714	0.077	1.000	0.500	1.000	0.714	0.077		0.000
SNP10	0.059	0.143	0.077	0.000	1.000	0.000	0.143	0.077	0.000	

D

LD Measure = Chi-square P value

	SNP1	SNP2	SNP3	SNP4	SNP5	SNP6	SNP7	SNP8	SNP9	SNP10
SNP1		0.058	0.047	0.525	0.343	0.525	0.058	0.047	0.525	0.795
SNP2	0.058		0.000	0.003	0.212	0.003	0.000	0.000	0.003	0.633
SNP3	0.047	0.000		0.772	0.108	0.772	0.000	0.000	0.772	0.813
SNP4	0.525	0.003	0.772		0.296	0.000	0.003	0.772	0.000	1.000
SNP5	0.343	0.212	0.108	0.296		0.296	0.212	0.108	0.296	0.011
SNP6	0.525	0.003	0.772	0.000	0.296		0.003	0.772	0.000	1.000
SNP7	0.058	0.000	0.000	0.003	0.212	0.003		0.000	0.003	0.633
SNP8	0.047	0.000	0.000	0.772	0.108	0.772	0.000		0.772	0.813
SNP9	0.525	0.003	0.772	0.000	0.296	0.000	0.003	0.772		1.000
SNP10	0.795	0.633	0.813	1.000	0.011	1.000	0.633	0.813	1.000	

LD Measure = Likelihood-ratio P value

	SNP1	SNP2	SNP3	SNP4	SNP5	SNP6	SNP7	SNP8	SNP9	SNP10
SNP1		0.145	0.140	1.000	0.510	1.000	0.150	0.145	1.000	1.000
SNP2	0.145		0.000	0.000	0.280	0.000	0.000	0.000	0.010	0.715
SNP3	0.140	0.000		0.800	0.045	0.735	0.000	0.000	0.780	1.000
SNP4	1.000	0.000	0.800		0.490	0.000	0.010	0.795	0.000	1.000
SNP5	0.510	0.280	0.045	0.490		0.455	0.325	0.060	0.455	0.085
SNP6	1.000	0.000	0.735	0.000	0.455		0.005	0.760	0.000	1.000
SNP7	0.150	0.000	0.000	0.010	0.325	0.005		0.000	0.000	0.695
SNP8	0.145	0.000	0.000	0.795	0.060	0.760	0.000		0.745	1.000
SNP9	1.000	0.010	0.780	0.000	0.455	0.000	0.000	0.745		1.000
SNP10	1.000	0.715	1.000	1.000	0.085	1.000	0.695	1.000	1.000	

Fig. 32. Open file for all information of LD measurement. SNP10-distance.xls in *example file folder* of $LD_2SNPing$ is used as an example. (A) *D* and *D'* values. (B) r^2 and δ value. (C) Q and ρ values. (D) Chi-square P value and likelihood-ratio P value. *Note!* The likelihood-ratio P value is dependent on the number of randomization and it shows minor variation for likelihood-ratio P value at different times. Please see the formula in the Appendix at the end of the user manual. Figs. A-D belongs to one WordPad file which is saved for output LD measure data. It is readable by *WordPad* and *Microsoft Word*. Here is the example of WordPad opening. **For Microsoft Word, the letter size needs to less than 9.**

4.3.5.9. Save and open image file for output LD measure data and graph

The LD$_2$SNPing provides the output for the LD graph such as Fig. 33. The steps to save and re-open the LD plot are demonstrated in Fig. 33B and Fig. 33C, respectively.

A
B
C

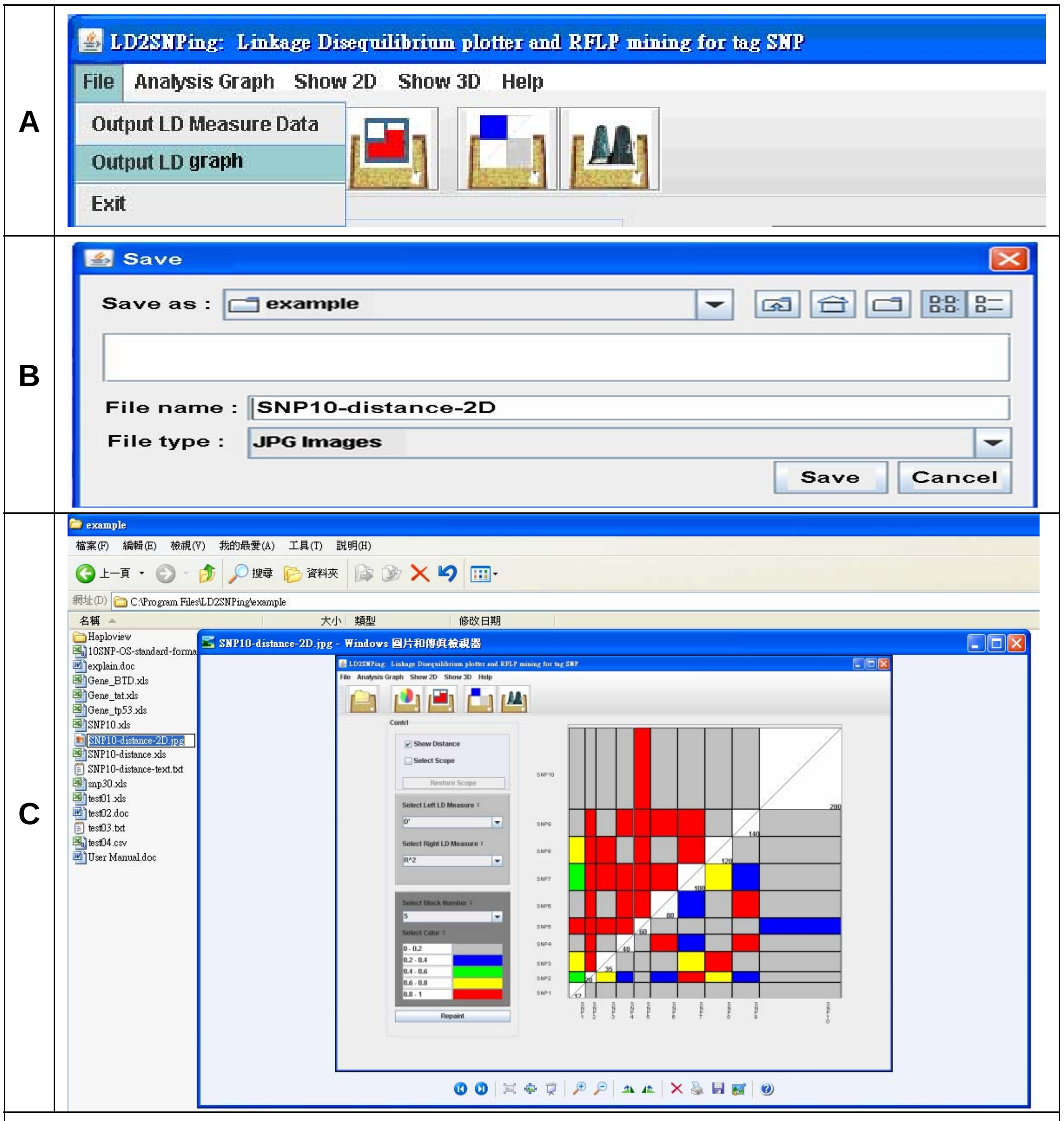

Fig. 33. Output graph information for saving and opening file. SNP10-distance.xls in *example file folder* of LD$_2$SNPing is used as an example. (A) Clicking the "output LD graph leads to the save function (B). Users are able to change the file location. (C) Users can re-open the graph by clicking the file under "file manager" of computer. Animation

4.3.5.10. <u>Comparison of LD plotting between JLIN and LD$_2$SNPing</u>

The performance and accuracy for LD$_2$SNPing are demonstrated by comparing to three common LD softwares, such as JLIN (Fig. 34), LDA (Fig. 35), and Haploview (Fig. 36). Since the JLIN only accept the .csv file, three example files (.csv) are used to test the visualization between LD$_2$SNPing and JLIN as shown in Fig. 33. Haploview needs the .ped and .info formats, therefore, the same data have to change format. Their 2D-LD plotting is completely matched.

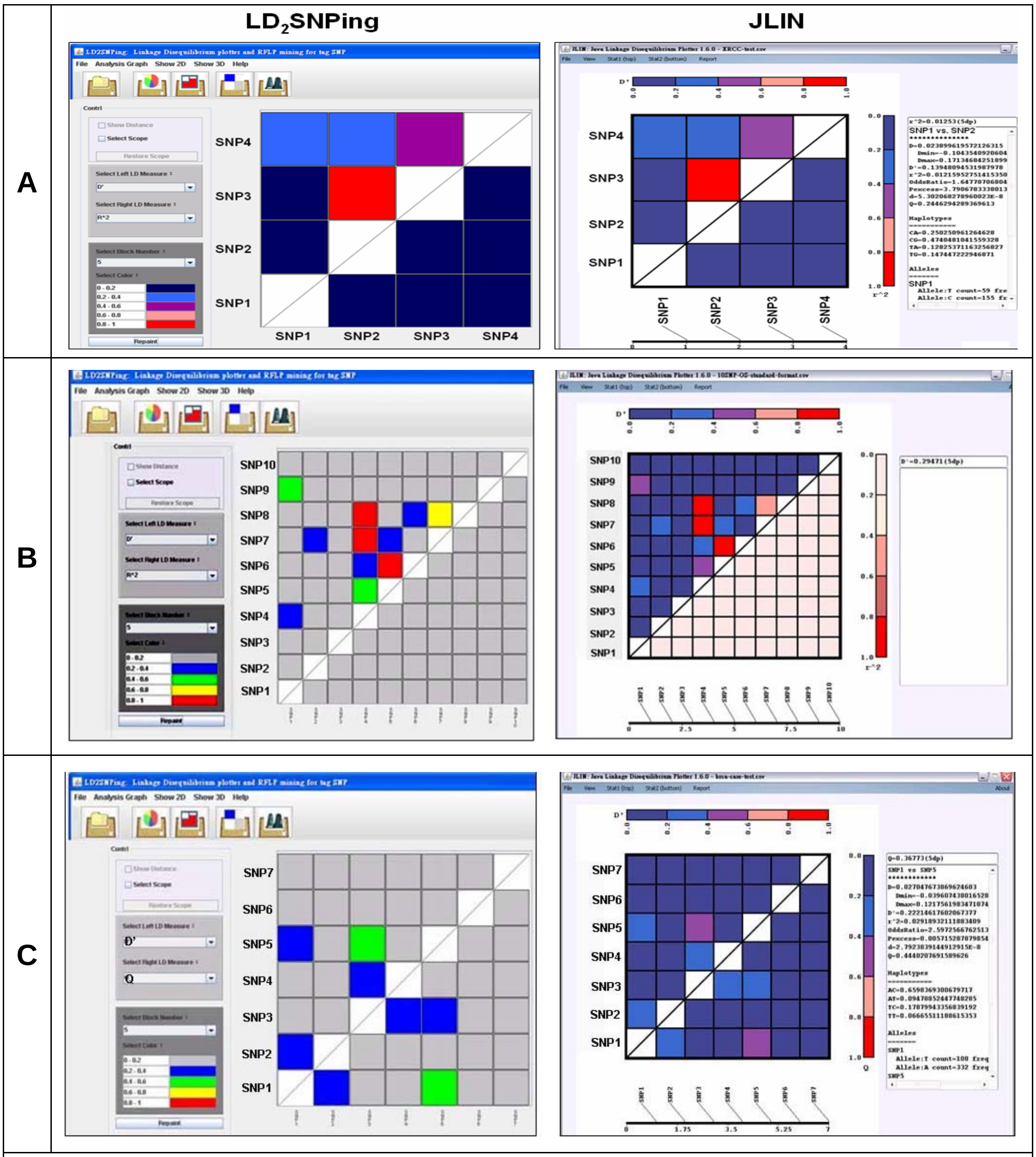

Fig. 34. Comparison of LD$_2$SNPing *vs.* JLIN 1.60. Test file sources: (A) <u>XRCC-test.csv</u> (*D'*/*r*^{2} for vertical/horizontal axis), (B) <u>10SNP-OS-standard-format.csv</u> (*D'*/*r*^{2}), and (C) <u>brca-case-test.csv</u> (*D'*/*Q*) in the *example file folder* of LD$_2$SNPing, respectively. The pattern between LD$_2$SNPing and JLIN is completely matched (same value with different color gradient between them).

4.3.5.11. Comparison of LD plotting between LDA and LD2SNPing

The visualization between LD2SNPing and LDA is compared as shown in Fig. 35. Their 2D-LD plotting is completely matched. Because the formula of calculating LD related information of LD2SNPing is derived from help system of LDA, the values for all LD-related information are confirmed to be the same for each other.

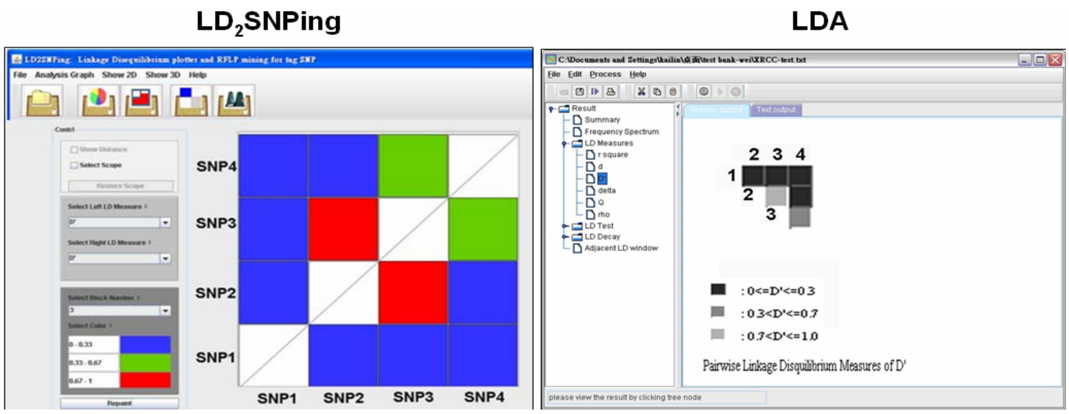

Fig. 35. Comparison of LD2SNPing *vs.* LDA. Test file source: XRCC-test.csv (*D'/D'*) in the *example file folder* of LD2SNPing.

4.3.5.12. Comparison of LD plotting between Haploview and LD$_2$SNPing

The visualization between LD$_2$SNPing and Haploview is compared as shown in Fig. 36. Their 2D-LD plotting is completely matched. Two example files are used to demonstrate the successful LD plotting.

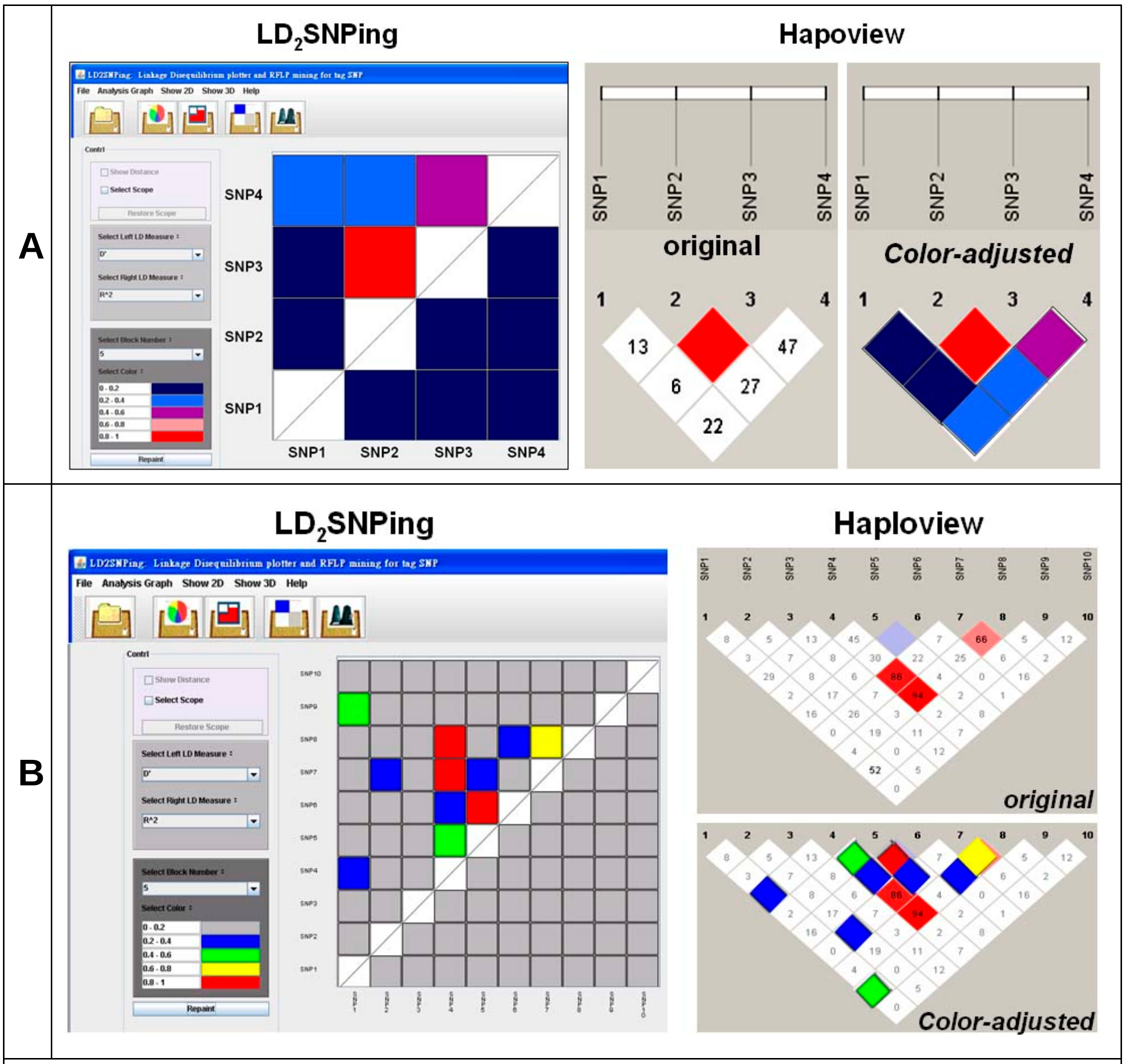

Fig. 36. Comparison of LD$_2$SNPing *vs.* Haploview. Color-adjusted plot is our manually modified according to the color gradient in LD$_2$SNPing. The number in the box of Haploview is the percentage of *D'* value. Test file sources: (A) XRCC-test.csv (*D'*/r^2 for vertical/horizontal axis) for LD$_2$SNPing and XRCC-test.ped & XRCC-test.info for Haploview in the *example file folder* of LD$_2$SNPing. (B) 10SNP-OS-standard-format.csv for LD$_2$SNPing and 10SNP-OS-standard-format.ped and 10SNP-OS-standard-format.info for Haploview in the *example file folder* of LD$_2$SNPing. White color in color-adjusted plot is the same pattern as the gray color in LD$_2$SNPing.

4.3.6. Graph analysis-related functions in 2D-LD plot

In addition to LD analysis and visualization, $LD_2SNPing$ also provides the some alternative function for LD analysis. The brief review for five graph analysis-related functions in 2D-LD plot is mentioned below.

4.3.6.1. Brief review for five graph analysis-related functions in 2D-LD plot

Since the function of the control panel is introduced above, we focus on the function with icons as shown in Fig. 37. They are described in detail later (after Fig. 38).

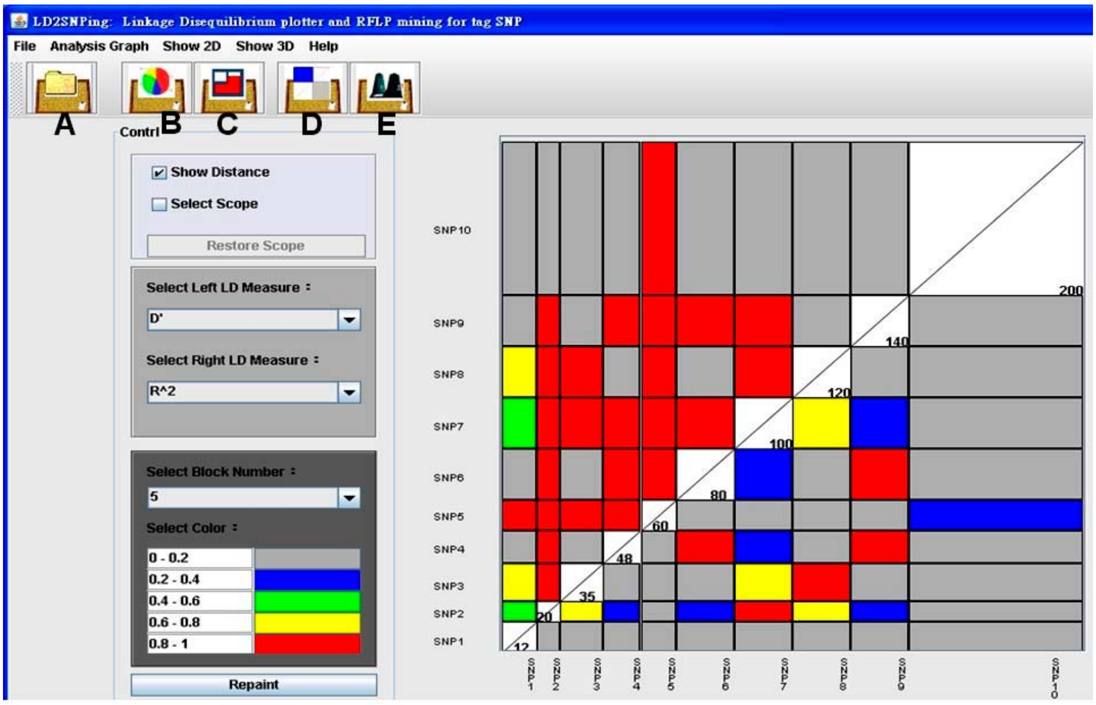

Fig. 37. Five graph analysis-related functions in 2D-LD plot. SNP10-distance.xls in *example file folder* of $LD_2SNPing$ is used as an example. (A) Close the file. (B) Show pie chart. (C) Show bar graph. (D) Show 2D-LD plot. (E) Show 3D-LD plot.

4.3.6.2. Close file

Sometimes, users may lose the way to go to the home screen of LD$_2$SNPing for another analysis. In stead of closing and restarting the LD$_2$SNPing, the first icon provides the “close file” function for homing (Fig. 38).

A

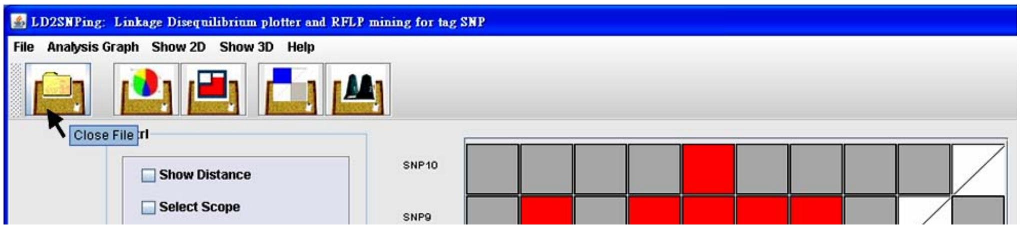

B

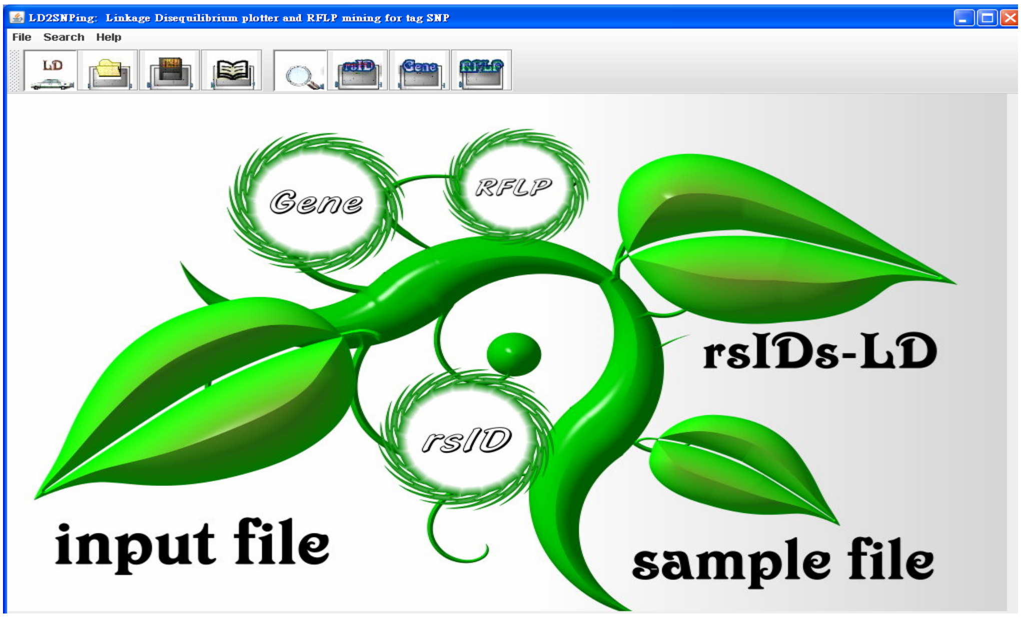

Fig. 38. Output information for saving file. The function of Fig. 37A is demonstrated. (A) Under any situations, users can click the “close file” indicated by arrow to stop the current LD analysis and return to the home screen for another new analysis (B). Animation

4.3.6.3. Analysis graph

4.3.6.3.1. Brief review for all functions in analysis graph

$LD_2SNPing$ provided some graphic analysis such as grid, bar and pie3D graph to supplement LD analysis to 2D-LD visualization and analysis (Fig. 39). Detail function is described in Figs. 40-44.

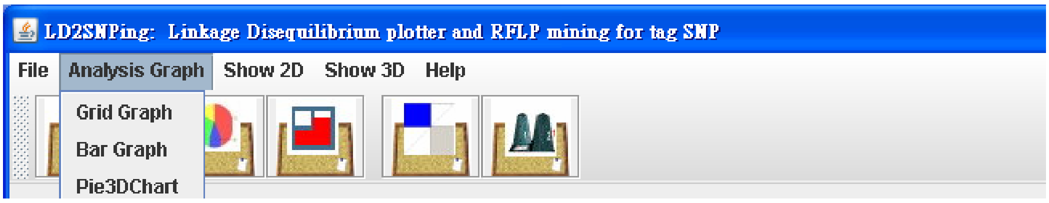

Fig. 39. Brief review for all functions in analysis graph. Grid, bar and pie3D graph are provided.

4.3.6.3.2. Analysis graph function- Grid graph

The steps to perform grid graph analysis in LD_2SNPing are shown in Fig. 40. The LD information from grid graph is in consistence with text data (Fig. 41).

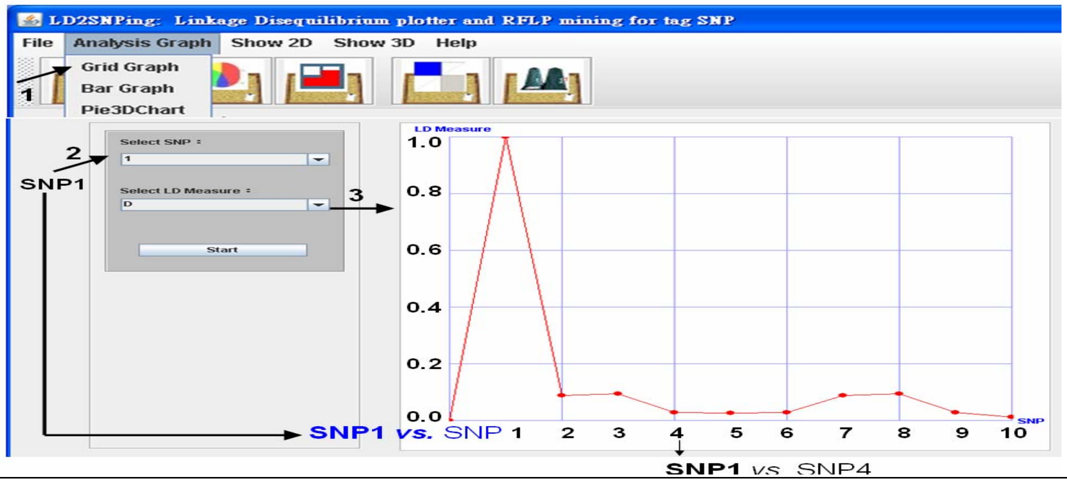

Fig. 40. Grid graph view. <u>SNP10-distance.xls</u> in *example file folder* of LD_2SNPing is used as an example. Clicking the arrow 1 to select the function of grid graph and then clicking the pull-down window to select the reference SNP (e.g., SNP1 indicated by arrow 2) to perform the pair-wised comparison to other SNPs. SNP1 vs. SNP1 shows the highest D value (pull-down selection indicated by arrow 3). Animation (Figs. 40 and 41 in an animation)

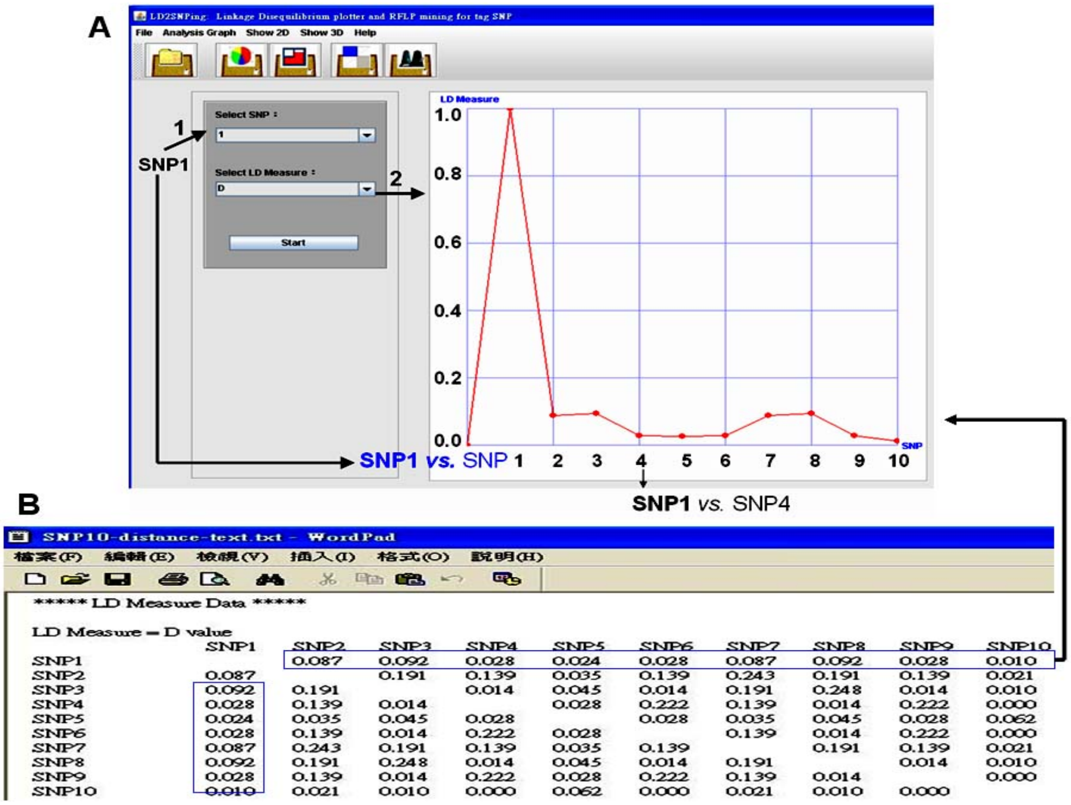

Fig. 41. Consistence for the LD information between (A) grid graph and (B) text data. Animation (Figs. 40 and 41 in an animation)

4.3.6.3.3. Analysis graph function- Bar graph

The steps to perform, save and open the bar graph analysis in LD$_2$SNPing are shown in Fig. 42.

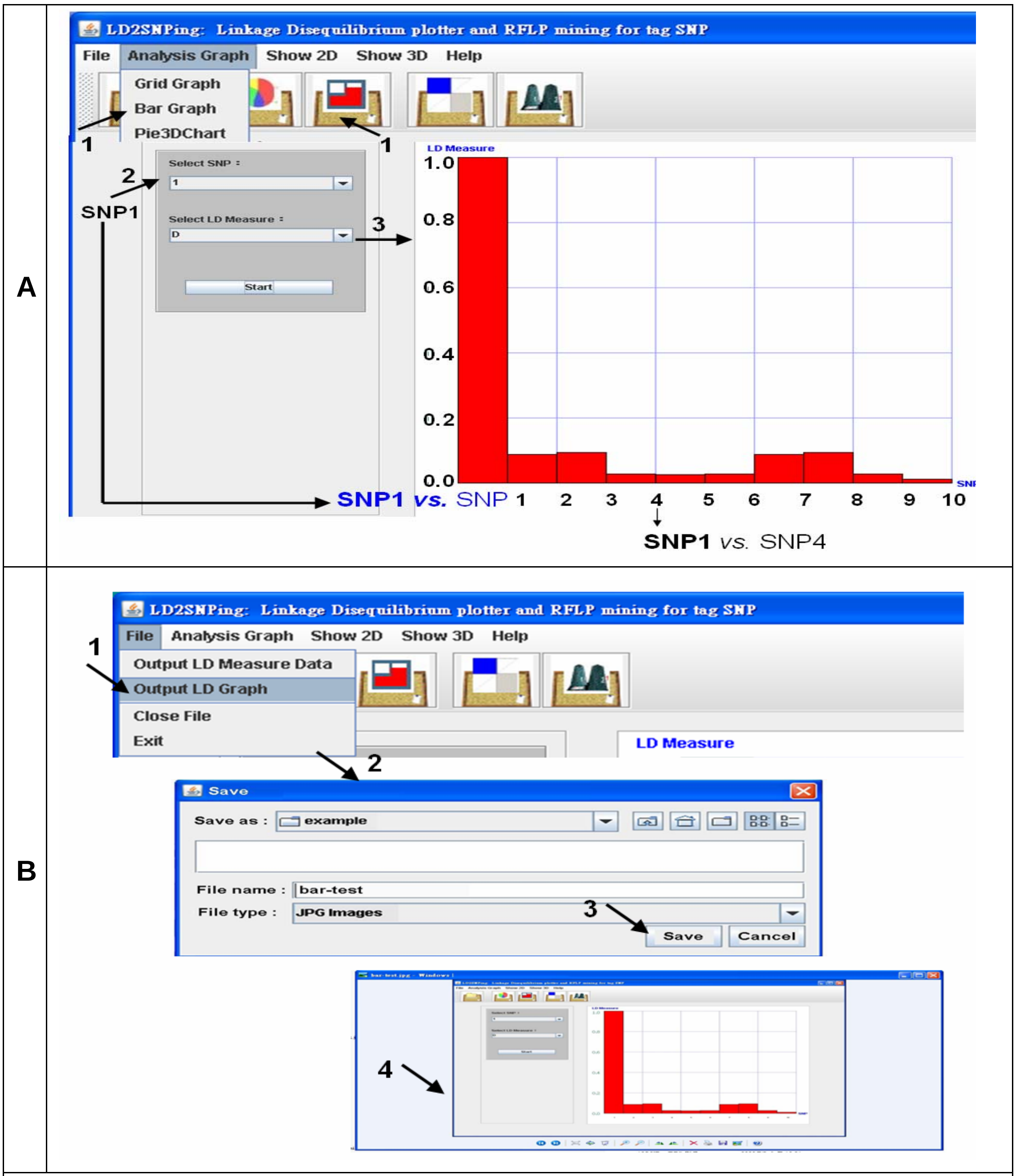

Fig. 42. To perform, save and open the bar graph analysis in LD$_2$SNPing. (A) To perform the bar graph analysis. (B) Output of bar graph analysis. Animation

4.3.6.3.4. Analysis graph function- Pie graph

LD$_2$SNPing also provides the pie3D to show the alleles distribution of selected SNP and file saving as shown in Fig. 43.

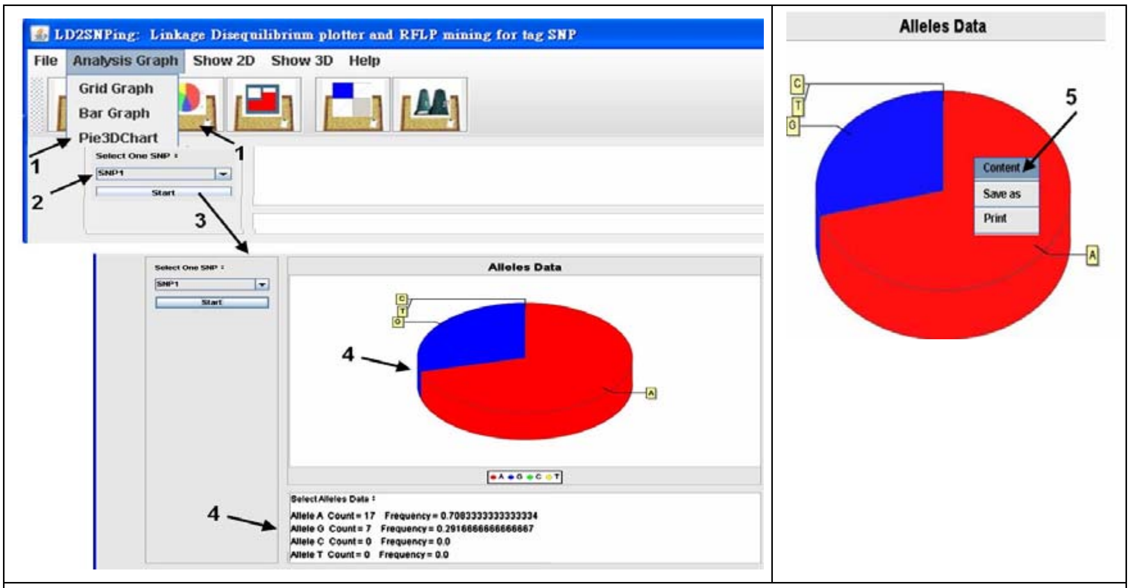

Fig. 43. The information of alleles. Step 1: Open the blank window of Pie3D chart. Step 2: Selecting single interested SNP from pull-down window as indicted by arrow 2. Step 3: Start to perform the function of pie3D chart for selected SNP. Step 4: All the nucleotide distribution in selected SNP is provided as indicated in arrow 4 either by figure or text. Step 5: Save and print the pie figure by clicking the right bottom of mouse. Animation

4.3.6.3.5. Return to 2D-LD plot

At anywhere, users can return to the first screen for 2D-LD visualization by clicking the “Show 2D” as indicated by arrow in Fig. 44.

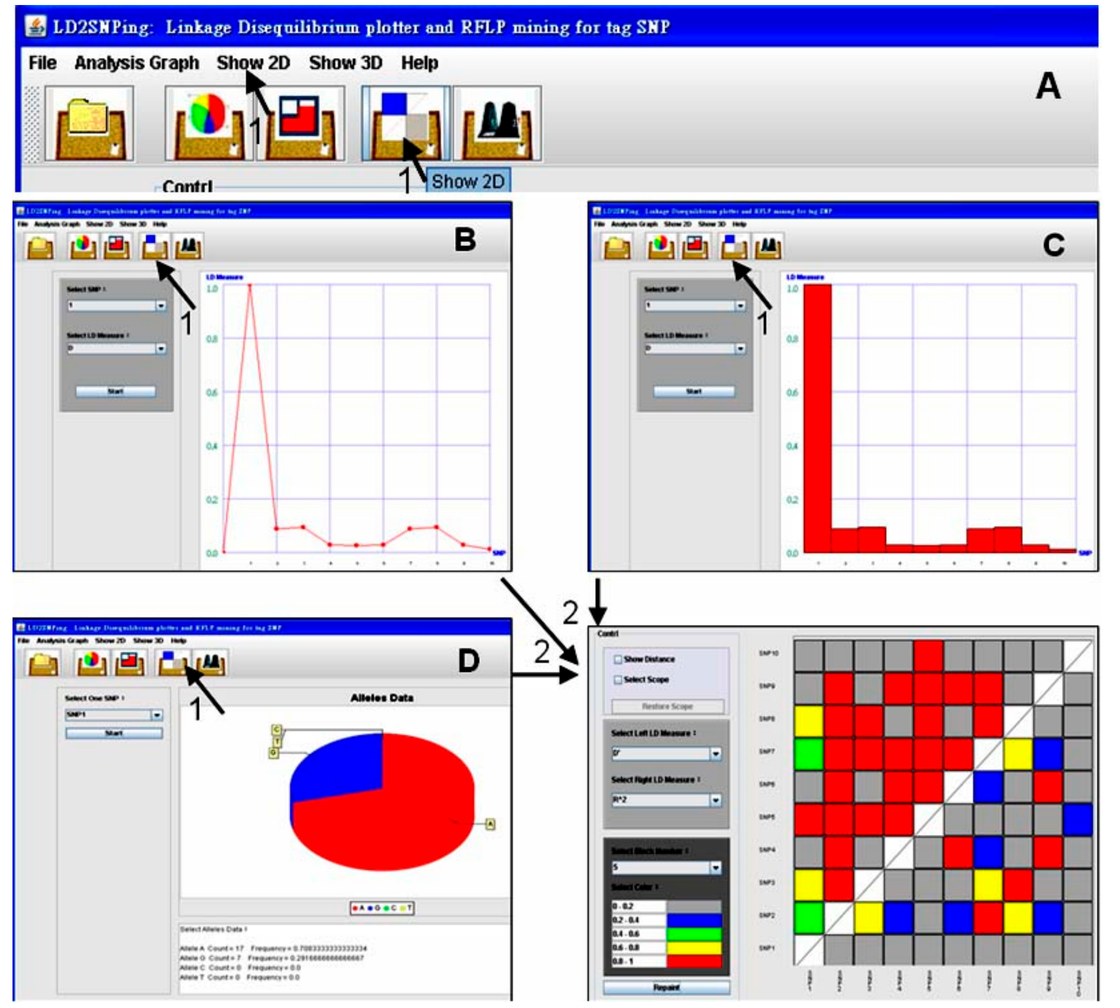

Fig. 44. Return to 2D-LD plot. Clicking the arrow 1 in Figs. 44A, 44B, 44C, and 44D can return to the original 2D-LD plot as indicated by arrow 2. Animation

4.3.7. LD in 3D visualization

4.3.7.1. The difference between 2-D and 3-D plotting

(1) The distance indicated by arrow is presented to the diagonal line with white patch in proportion to its height.

(2) Although the absolute values for all the SNP may not be able to read in 3-D plotting, the 3-D plotting provides the brief and clear view between the SNPs with the high and low values without checking the gradient color scale as provided in 2-D plotting.

4.3.7.2. Performing 3D-LD and changing the parameters in 3D-LD plot

$LD_2SNPing$ use Java 3D coded on the program construction. In Fig. 45, the steps for performing 3D-LD and changing the parameters in 3D-LD plot are shown.

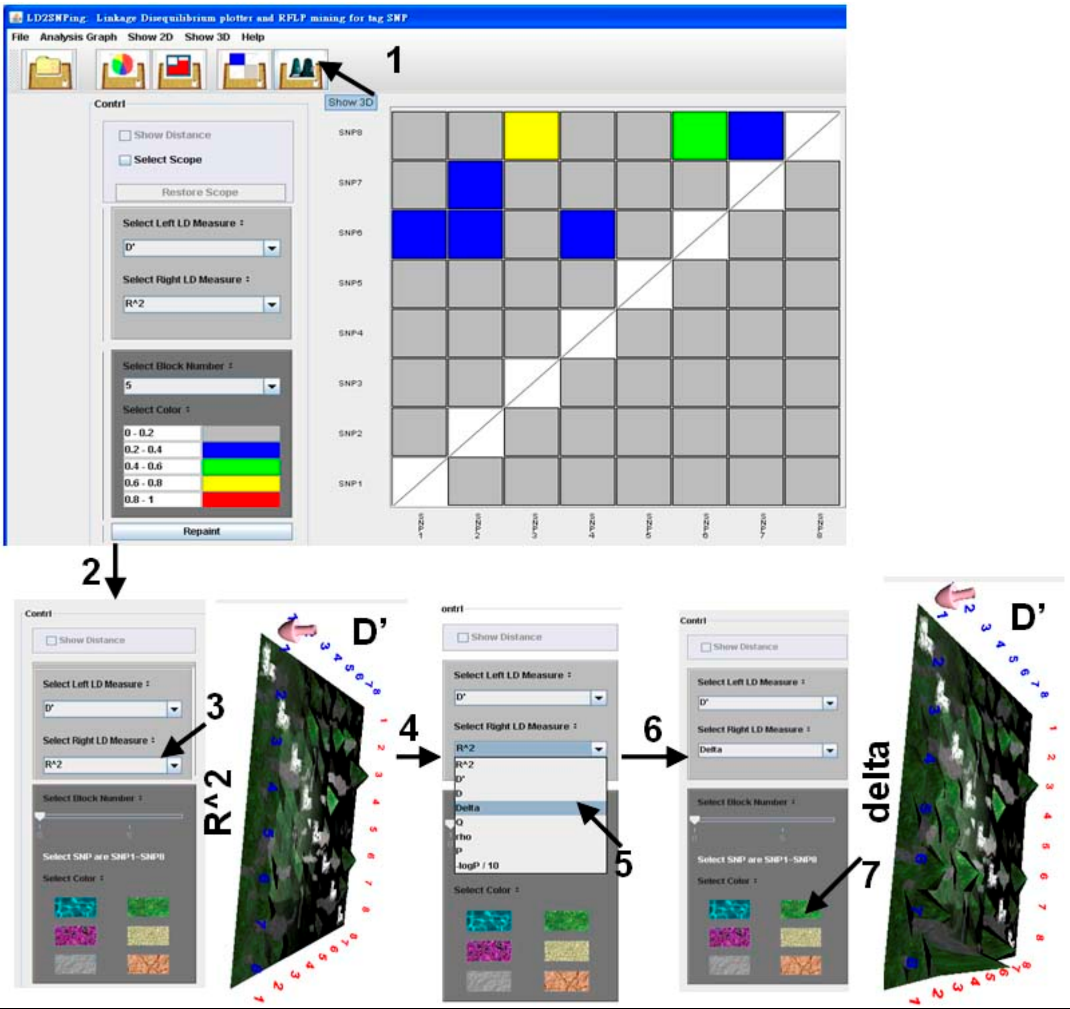

Fig. 45. Performing 3D-LD and changing the parameters in 3D-LD plot. From step 1 to step 7, users can visualize the 3D-LD plot, change the parameter for left and right side of pink pushpin, and change the color of 3D-LD plot. Animation

4.3.7.3. Representative view of 3D-LD plot

The relationship of visualization between 2D-LD and 3D-LD plots is described in Fig. 46. The distance between SNPs is visualized to both 2D-LD and 3D-LD plots if the distance information is available in SNP data set. The height of the diagonal line in 3D-LD plot is in proportion to its distance between SNPs in reference to the first SNP.

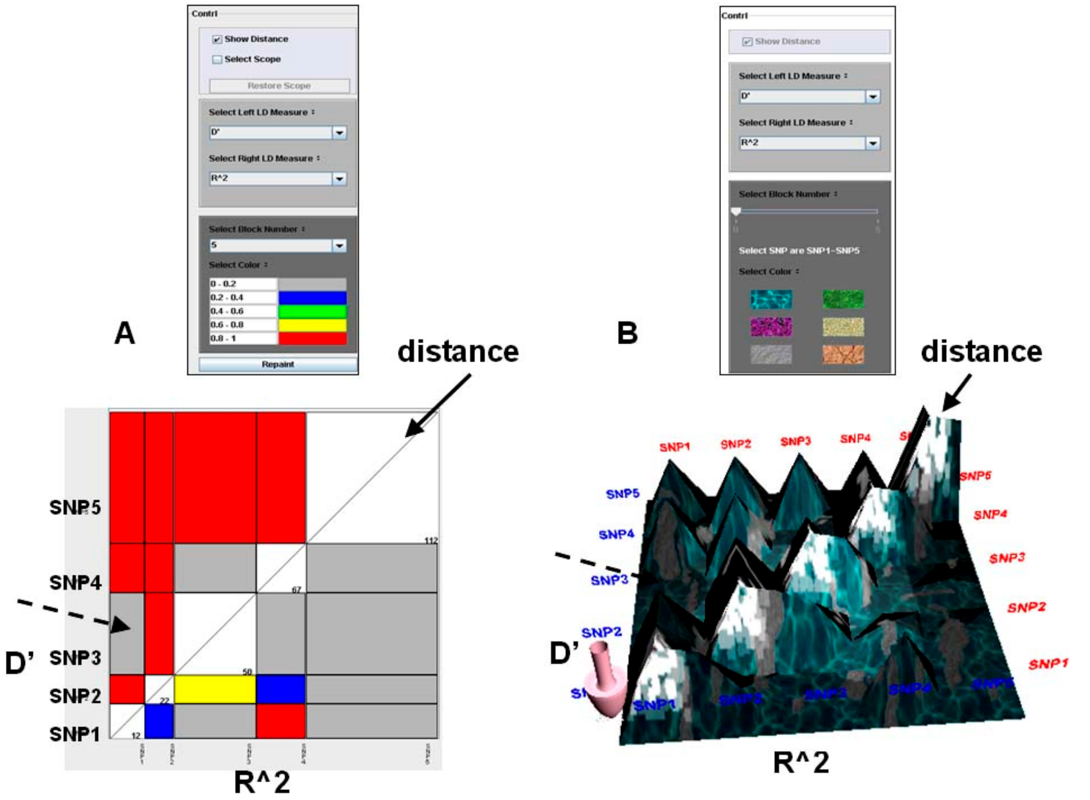

Fig. 46. Comparison of visualization between 2D-LD and 3D-LD. This file is available from *example file folder* of LD$_2$SNPing (e.g., test01.xls). (A) and (B) are the visualization for 2D-LD and 3D-LD, respectively. The distance indicated by arrow is presented to the diagonal line with white patch in proportion to its height. The scales for left and right LD measure are changeable as mentioned in 2D-LD plot. Broken arrow line in (A) indicates the lower *D'* value for SNP3 *vs.* SNP1. In consistence, it is clear that no mountain appears in SNP3 *vs.* SNP1 (B). Please see Fig. 47 for detail modification steps. Animation

4.3.7.4. Zoom-in, zoom-out and rotation of 3D-LD plot

LD$_2$SNPing can rotate the orientation of 3D-LD plot as well as zoom-in and zoom-out (Fig. 47). In order to avoid out of memory, it provides maximal 10 SNPs for 3D-LD visualization. Six different graphic screens are provided for personal preferences.

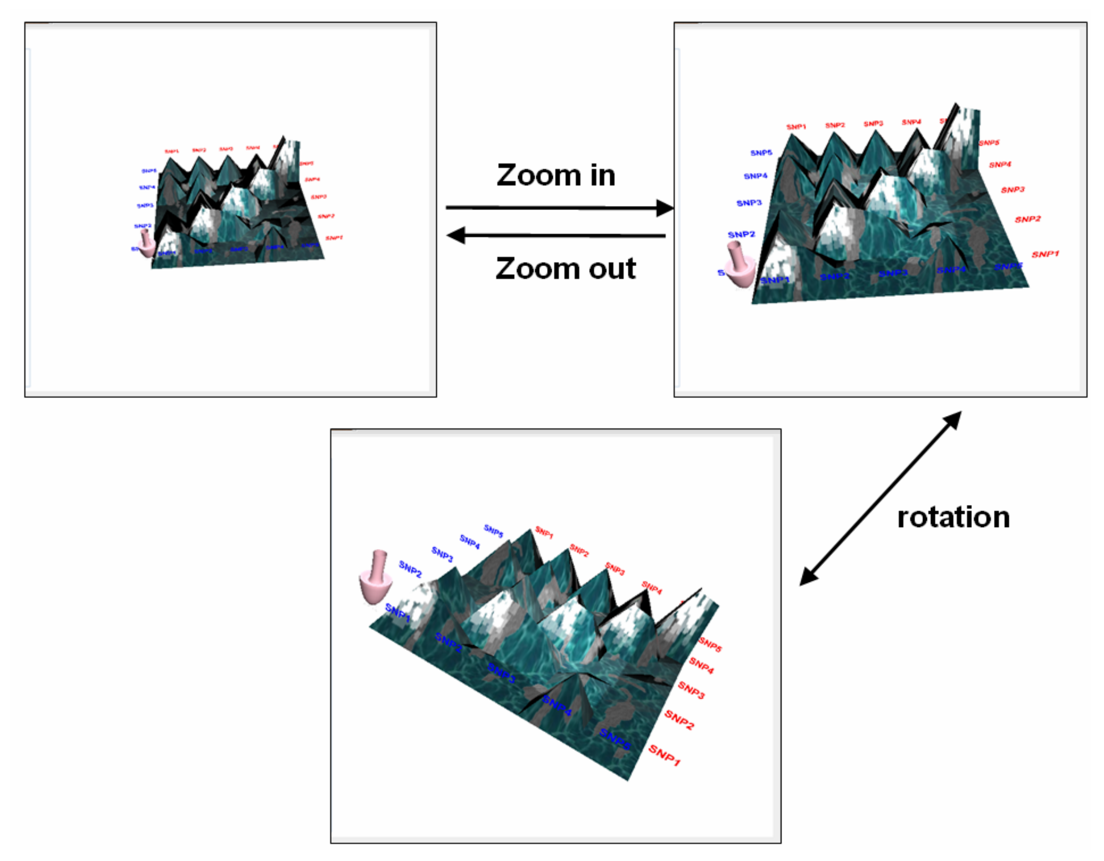

Fig. 47. Zoom-in, zoom-out and rotation of 3D-LD plot. This file is available from *example file folder* of LD$_2$SNPing (e.g., test01.xls). Mouse moving under consistent pressing the left bottom of mouse can rotate the orientation of 3D-LD plot. Rolling the wheel of the mouse also provides the zoom-in and zoom-out function. Animation

4.3.7.5. Changing color for 3D-LD plot

Six colors are available for selection in 3D-LD plotting as shown in Fig. 48.

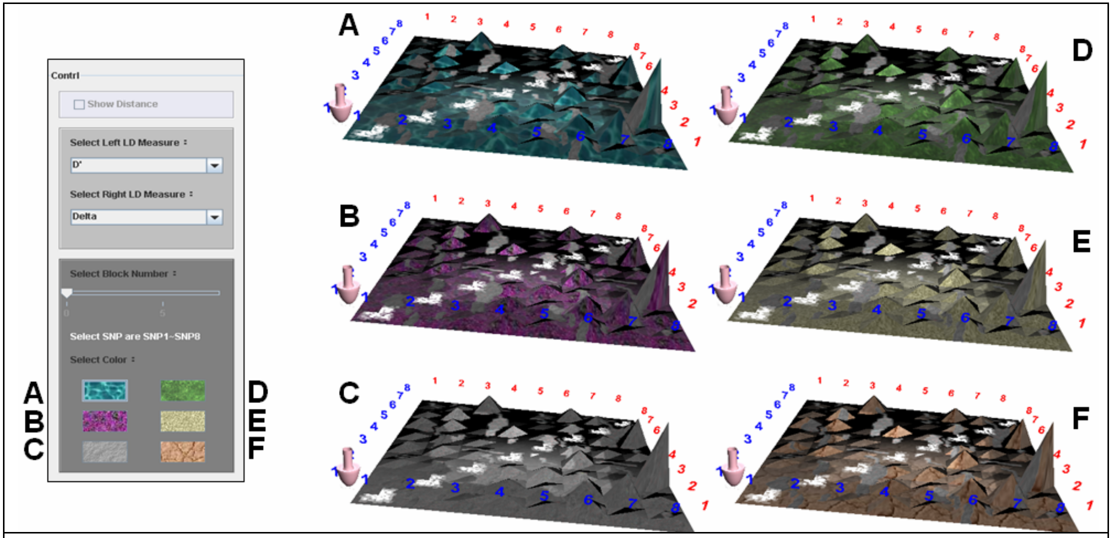

Fig. 48. Changing color for 3D-LD plot. Clicking anyone of the "select color" box indicated by A to F in left side allows the users to change the 3D color. This 2D-LD plot is drawn using *sample file 2* of $LD_2SNPing$. No distance information in original data set in the sample file 2 and therefore the diagonal line with white patch is flat. Animation

4.3.7.6. Selection of block number for 3D-LD plot

The current version of LD$_2$SNPing accepts 10 SNPs for maximal visualization. In the Fig. 49, SNP2-SNP21 and SNP10-SNP31 are screened out the non-SNP variant. Only the SNPs are provided.

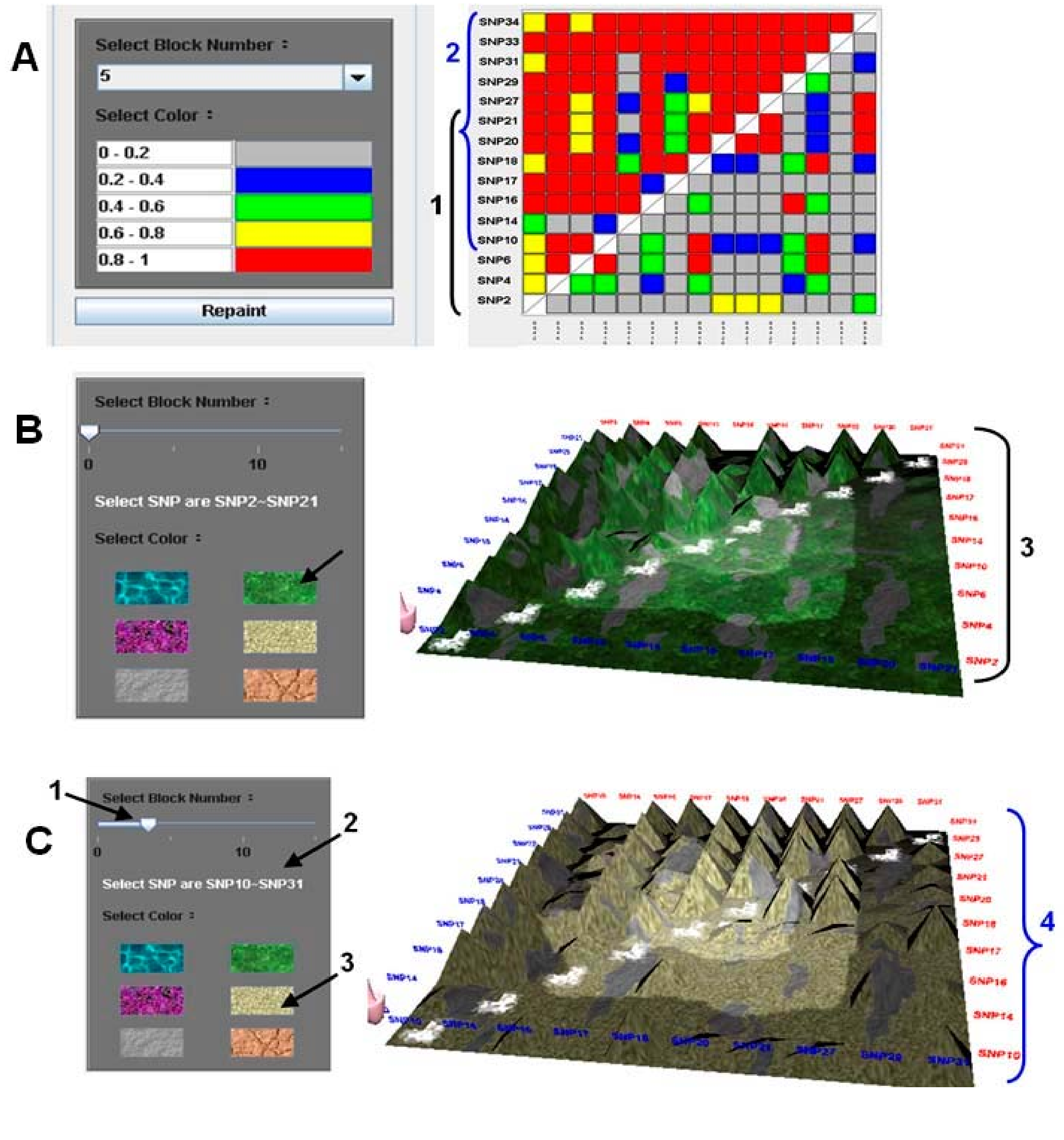

Fig. 49. Selecting 10 SNPs for 3D-LD plot. For some nucleotide variants less than 1% (not belong to the SNP) are not shown in both 2D-LD (Fig. 15) and 3D-LD plots (Fig. 49), respectively. The current version of LD$_2$SNPing accepts 10 SNPs for maximal visualization. (A) The 2D-LD plot is the same as in Fig. 15. 10 SNPs in brackets 1 (Fig. 49A) are SNPs 2, 4, 6, 10, 14, 16, 17, 18, 20, and 21, which is the same SNPs to bracket 4 (Fig. 49B). (B) Clicking the arrow can change the color. (C) Clicking the arrow 1 can change the selection of block number. The white arrow head is moved to right and the “select SNP” is changed to SNP10-SNP31. 10 SNPs in brackets 2 (Fig. 49A) are SNPs 10, 14, 16, 17, 18, 20, 21, 27, 29, and 31, which are the same SNPs to bracket 4 (Fig. 49C). Animation

5. Help

Anytime user can find the help before and after LD plotting (Fig. 50).

A

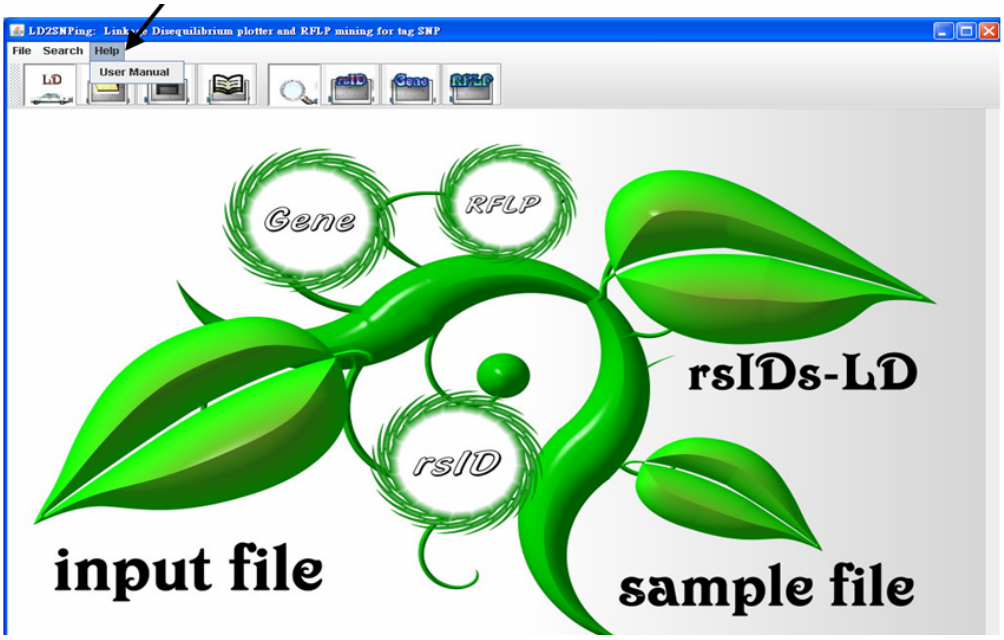

B

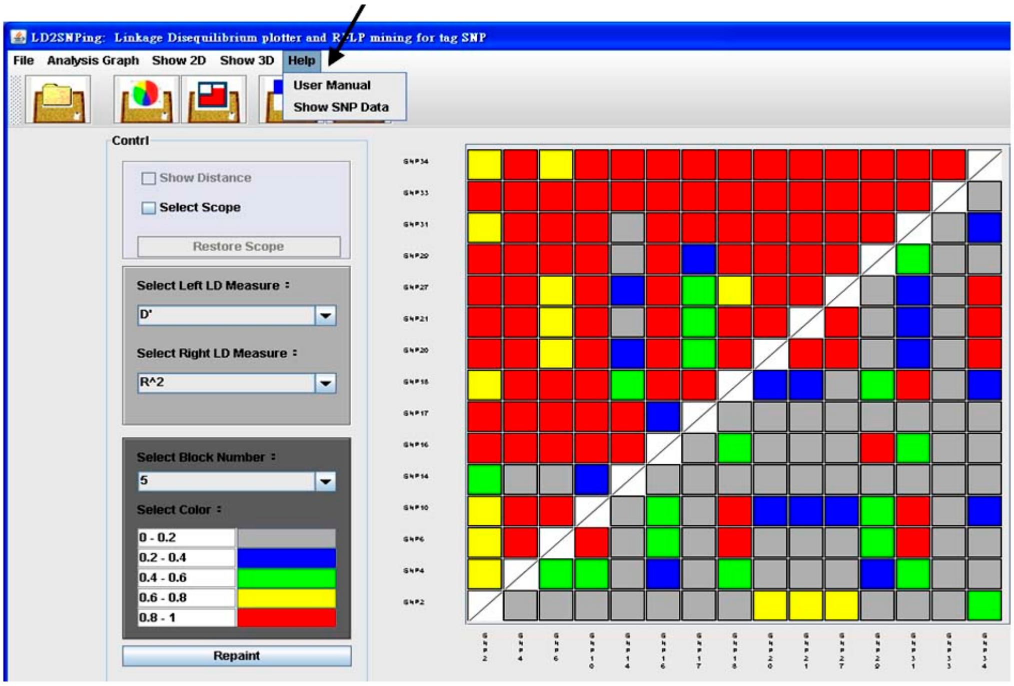

Fig. 50. Help before and after LD analysis. (A) Before and (B) after LD analysis. Help function is performed by clicking the help box as indicated by arrow lines. (B) It provides extra function to show SNP information for LD plotting such as missing data, MAF, genotype source.

6. Appendix

		SNP2		
		B	b	Total
SNP1	A	P_{AB}	P_{ab}	P_A
	a	P_{aB}	P_{ab}	P_a
	Total	P_B	P_b	

Table 1 – Formula for linkage disequilibrium measurement [ref. 1]

**Symbol**	**formula**
D (The difference in proportions)	$\frac{P_{AB}P_{ab} - P_{Ab}P_{aB}}{P_{AB}P_{ab} + P_{Ab}P_{aB}}$
D′ (The Lewontin’ s *D′*)	$\frac{P_{AB}P_{ab} - P_{Ab}P_{aB}}{\min(P_A P_B, P_a P_b)} (D < 0)$<br>$\frac{P_{AB}P_{ab} - P_{Ab}P_{aB}}{\min(P_A P_b, P_B P_a)} (D > 0)$
r^2 (The square of the standardized measures)	$\frac{(P_{AB}P_{ab} - P_{Ab}P_{aB})^2}{P_A P_a P_B P_b}$
δ (The Delvin’s population attributable factor)	$\frac{P_{AB}P_{ab} - P_{Ab}P_{aB}}{P_B P_{ab}}$
Q (The Yule’s Q)	$\frac{P_{AB}P_{ab} - P_{Ab}P_{aB}}{P_{AB}P_{ab} + P_{Ab}P_{aB}}$
ρ	$\frac{P_{AB}P_{ab} - P_{Ab}P_{aB}}{P_A P_b}$
P value of likelihood-ratio test (LD)	*See* note 1 later
P value of Chi-square test (LD)	*See* note 1 later
P value of Chi-square test (Hardy-Weinberg Equilibrium)	*See* note 2 later

Note 1- *P* values for Linkage Disequilibrium Test

Calculating Likelihood-ratio Test

1. **Number of Randomization**: The number of randomization test in permutation. (The default set no. in $LD_2SNPing$ is set at 1000; The default set no. in LDA is 100, 1000, or 10000.)
2. **Likelihood**:

   L_o：The likelihood under the assumption of no disequilibrium;

   L_e：The likelihood computed using the haplotype frequencies found by the EM algorithm
3. **Statistic S**:

$$S = 2.0 * \log_e \frac{L_e}{L_0} \quad \text{(Sobs \& Sran)}$$

4. **P value**:

$$P = \frac{\sum_{i=1}^{\text{Permutations}} (\text{if Sran} <= \text{Sobs}, \text{count} + 1)}{\text{Permutations}}$$

Chi-square Test

1. $\chi^2 = \dfrac{2nD^2}{P_A * P_a * P_B * P_b}$
2. **P value** is calculated from incomplete gamma function. (See Numerical Recipes in C, The 2nd edition. CAMBRIDGE UNIVERSITY PRESS)

Note 2. Hardy-Weinberg Equilibrium tests [ref. 9]

1. $P(N_{AB} = n_{AB} \mid N, n_A) = \dfrac{\theta^{n_{AB}/2} N!}{n_{AA}! n_{AB}! n_{BB}!} \times \dfrac{1}{C}$

   where $C = \sum \dfrac{\theta^{n^*_{AB}/2} N!}{n^*_{AA}! n^*_{AB}! n^*_{BB}!}$

   Define the quantity $\theta = p^2_{AA} / p_{AA} p_{BB}$ so that $\theta = 4$ when HWE holds.
2. $P_{HWE} = \sum_{n^*_{AB}} I[P(N_{AB} = n_{AB} \mid N, n_A) \geq P(N_{AB} = n^*_{AB} \mid N, n_A)] \times P(N_{AB} = n^*_{AB} \mid N, n_A)$

7. References:

1.LDA--a java-based linkage disequilibrium analyzer. Bioinformatics. 2003; 19(16):2147-8. http://www.chgb.org.cn/lda/lda.htm

2.JLIN: a java based linkage disequilibrium plotter. BMC Bioinformatics. 2006; 7:60. http://www.genepi.org.au/projects/jlin

3.Haploview: analysis and visualization of LD and haplotype maps. Bioinformatics. 2005; 21(2):263-5. http://www.broad.mit.edu/mpg/Haploview/

4.HelixTree. http://www.goldenhelix.com/SNP_Variation/HelixTree/index.html

5.HapMap. http://www.hapmap.org

6.dbSNP of NCBI. http://www.ncbi.nlm.nih.gov/SNP/

7.REBASE--enzymes and genes for DNA restriction and modification. Nucleic Acids Res. 2007;35(Database issue):D269-70. http://rebase.neb.com/rebase/rebase.html

8.SNP-RFLPing: restriction enzyme mining for SNPs in genomes. BMC Genomics. 2006; 7:30. http://bio.kuas.edu.tw/snp-rflp

9.A note on exact tests of Hardy-Weinberg equilibrium. American journal of human genetics 2005; 76:887-93.
